# Transcriptomic analysis of lignocellulosic biomass degradation by the anaerobic fungal isolate *Orpinomyces* sp. strain C1A

**DOI:** 10.1186/s13068-015-0390-0

**Published:** 2015-12-08

**Authors:** M. B. Couger, Noha H. Youssef, Christopher G. Struchtemeyer, Audra S. Liggenstoffer, Mostafa S. Elshahed

**Affiliations:** Department of Microbiology and Molecular Genetics, Oklahoma State University, Stillwater, OK USA; Department of Biology and Health Sciences, McNeese State University, Lake Charles, LA USA

**Keywords:** Anaerobic gut fungi, Lignocellulosic biomass, Cellulosome, Transcriptomics

## Abstract

**Background:**

Anaerobic fungi reside in the rumen and alimentary tract of herbivores where they play an important role in the digestion of ingested plant biomass. The anaerobic fungal isolate *Orpinomyces* sp. strain C1A is an efficient biomass degrader, capable of simultaneous saccharification and fermentation of the cellulosic and hemicellulosic fractions in multiple types of lignocellulosic biomass. To understand the mechanistic and regulatory basis of biomass deconstruction in anaerobic fungi, we analyzed the transcriptomic profiles of C1A when grown on four different types of lignocellulosic biomass (alfalfa, energy cane, corn stover, and sorghum) versus a soluble sugar monomer (glucose).

**Results:**

A total of 468.2 million reads (70.2 Gb) were generated and assembled into 27,506 distinct transcripts. CAZyme transcripts identified included 385, 246, and 44 transcripts belonging to 44, 13, and 8 different glycoside hydrolases (GH), carbohydrate esterases, and polysaccharide lyases families, respectively. Examination of CAZyme transcriptional patterns indicates that strain C1A constitutively transcribes a high baseline level of CAZyme transcripts on glucose. Although growth on lignocellulosic biomass substrates was associated with a significant increase in transcriptional levels in few GH families, including the highly transcribed GH1 β-glucosidase, GH6 cellobiohydrolase, and GH9 endoglucanase, the transcriptional levels of the majority of CAZyme families and transcripts were not significantly altered in glucose-grown versus lignocellulosic biomass-grown cultures. Further, strain C1A co-transcribes multiple functionally redundant enzymes for cellulose and hemicellulose saccharification that are mechanistically and structurally distinct. Analysis of fungal dockerin domain-containing transcripts strongly suggests that anaerobic fungal cellulosomes represent distinct catalytic units capable of independently attacking and converting intact plant fibers to sugar monomers.

**Conclusions:**

Collectively, these results demonstrate that strain C1A achieves fast, effective biomass degradation by the simultaneous employment of a wide array of constitutively-transcribed cellulosome-bound and free enzymes with considerable functional overlap. We argue that the utilization of this indiscriminate strategy could be justified by the evolutionary history of anaerobic fungi, as well as their functional role within their natural habitat in the herbivorous gut.

**Electronic supplementary material:**

The online version of this article (doi:10.1186/s13068-015-0390-0) contains supplementary material, which is available to authorized users.

## Background

Lignocellulosic biomass is a vast and underutilized resource for the production of biofuels. Compared to current schemes that rely on edible crops, lignocellulosic biomass utilization for sugar and biofuel production offers multiple advantages. It is abundant, renewable, and alleviates the moral stigma of using edible crops for industrial purposes. Further, the utilization of available lignocellulosic biomass overcomes the need for expanding farming acreage, and the subsequent increase in input of chemical fertilizers to the environment [1–3].

One of the most important procedures for the production of lignocellulosic biofuels involves the utilization of enzymes to extract sugar from plant polymers. The extracted sugars are then converted into biofuel using dedicated sugar-fermenting microorganisms [4]. However, the sugar extraction process from lignocellulosic biomass is far more complicated than sugar extraction from cereal grains (mainly corn in the US) [5]. This is due to the fact that the target substrates in lignocellulosic biomass (cellulose and hemicellulose) are structural components of plant cell walls, which are chemically bound to a variety of complex macromolecules (mainly lignin) [6]. Therefore, a combination of chemical pretreatments and the addition of exogenous enzyme cocktails are required for their effective mobilization and deconstruction [7, 8]. Enzymatic treatment of lignocellulosic biomass is a complex endeavor requiring multiple enzymes, a fact that significantly raises the cost of the process.

One alternative that circumvents the need for harsh pretreatments and exogenous enzyme amendments for the extraction of sugar monomers from lignocellulosic biomass is the use of specialized microbial cultures for biomass deconstruction [9–11]. Microbial strains capable of cellulose and/or hemicellulose degradation produce not only cellulolytic and xylanolytic enzymes targeting the backbone of these polymers, but also multiple accessory enzymes for removing side chains and breaking lignin–hemicellulose bonds [12–14]. Of special interest are lignocellulolytic microbes exhibiting anaerobic fermentative mode of metabolism since a significant fraction of the starting substrates could be recovered as a fermentation end product.

The anaerobic gut fungi (Phylum Neocallimastigomycota) are unique in combining the resilience and invasiveness of fungi with the metabolic capabilities of anaerobic fermentative prokaryotes [15]. Anaerobic fungi are inhabitants of the rumen and alimentary tract of herbivores where they play an important role in the metabolism of ingested plant material [16]. It has been established that in such habitats these organisms play a role akin to their aerobic counterparts in soils and streams. By attaching themselves to plant materials, they colonize and excrete extracellular enzymes that mobilize the structural plant polymers to be available to other microbes. Anaerobic fungi possess a powerful cellulolytic and hemicellulolytic enzymatic machinery [12] that aids in the required fast and efficient degradation of plant material in its relatively short residence time within the herbivorous gut [17]. Such capabilities have been demonstrated through experimental evaluation of anaerobic fungal isolates [18–21], biochemical characterization of anaerobic fungal enzymes [12], and recent genomic analysis of their lignocellulolytic repertoire [22].

We are currently exploring the utility of an anaerobic fungal isolate (*Orpinomyces* sp. strain C1A, henceforth referred to as strain C1A) for use in a consolidated bioprocessing framework for biofuel production. Developing an understanding of the genetic and regulatory mechanisms that enable efficient biomass degradation by strain C1A is central to gauging its potential as a sugar extraction platform in biofuel production schemes. Our previous efforts have documented the lignocellulosic biomass-degrading capabilities of C1A [22, 23] and the expansion of carbohydrate-active enzymes (CAZymes) in its genome [22]. However, key questions regarding strain C1A lignocellulolytic capabilities remain unanswered. For example, patterns of differential transcription of various CAZyme families, especially those mediating apparently similar enzymatic activities, when grown on different types of substrates are currently unclear. Similarly, the differential transcriptional patterns and putative contribution to biomass degradation of the large number of CAZyme genes identified in C1A genome have not been investigated in anaerobic fungi. Finally, the transcriptional profiles and differential transcriptional patterns of fungal dockerin-containing (putatively cellulosome-bound) have yet to be determined in anaerobic fungi.

Here we present a detailed comparative analysis of the transcriptomic profiles of C1A when grown on four different types of lignocellulosic biomass (alfalfa, energy cane, corn stover, and sorghum), versus a soluble sugar monomer (glucose). Our analysis aimed at addressing the patterns of regulation of lignocellulosic gene transcription in C1A, the contribution of various CAZyme gene families to biomass degradation in C1A, and the significance of gene expansion and duplication observed in the C1A genome on its lignocellulolytic capabilities.

## Results

### RNA-Seq output summary

A total of 468,159,494 (70.2 Gb) quality-filtered reads were used for transcriptome assembly and quantitative RNA-Seq analysis (Table 1). The number of reads generated for each growth condition ranged from 58.61 million (8.7 Gb) in alfalfa-grown cultures to 141.24 million (21.19 Gb) in sorghum-grown cultures (Table 1). This level corresponds to 88.73X-201.77X genomic coverage, and 426.73X-1115.07X predicted cDNA coverage. The generated assembly had an N50 of 1319 bp. A total of 27,506 distinct transcripts with predicted peptides were identified in the assembly.

**Table 1:** General statistics of RNA-Seq output

Condition	Total reads	Total bases	Genome coverage^a^	cDNA coverage^b^	Assembled transcript coverage^c^	*R* ^2^ value^c^
Glucose	81,468,482	12,220,272,300	121.59	590.27	349.15	0.89
Alfalfa	58,612,544	8,791,881,600	87.48	424.67	251.20	0.99
Energy cane	93,381,914	14,007,287,100	139.38	676.58	400.21	0.99
Corn stover	100,842,114	15,126,317,100	150.51	730.63	432.18	0.99
Sorghum	141,241,616	21,186,242,400	210.81	1023.34	605.32	0.99
Total reads/coverage	468,159,494	70,223,924,100	698.75	3391.97	2038.05	0.99

^a^Genome coverage based on an estimated 100.5 Mb genome size [18]

^b^cDNA coverage is based on a 20.76 % genome coding density [18]

^c^Assembled transcript coverage is based on the total assembled transcript size (35.0 Mb)

### Strain C1A CAZymes and potential lignocellulolytic capabilities

A total of 385, 246, and 44 distinct transcripts belonging to 44, 13, and 8 different GH, CE, and PL families, respectively, were identified in at least one condition (Table 2), with the majority being transcribed under all five growth conditions examined (Additional file 1: Figure S1). Collectively, the CAZyme transcripts identified demonstrate the capability of strain C1A to degrade cellulose (putative endoglucanases of GH5, GH9, GH45, GH48, and GH124; cellobiohydrolases of GH6 and GH48; β-glucosidases of GH1 and GH3), major types of hemicellulose including arabinoxylans/glucuronoarabinoxylans (putative xylanases of GH10 and GH11; β-xylosidases and α-l-arabinofuranosidases of GH39 and GH43; β-galactosidases of GH2; α-glucuronidases of GH67 and GH115), glucomannans/galactoglucomannans (putative mannanases and mannosidases of GH26; β-galactosidases of GH2), mixed glucans (putative β-(1–3, 1–4) endoglucanase of GH16; β-glucosidases of GH1 and GH3), and xyloglucans (putative xyloglucanases of GH67 and GH74; α-fucosidase of GH95). In addition to cellulose and hemicellulose, transcripts indicative of the capacity to degrade laminarin (putative 1,3-β-d-endoglucanase of GH55 and GH64; β-glucosidases of GH1 and GH3), starch (putative α-amylase of GH13 and GH119; α-amylase/amylopullulanase of GH57), pectin (putative polygalacturonase of family GH28; endo-β-1,4-galactanase of family GH53; α-l-rhamnosidase of GH78; unsaturated rhamnogalacturonyl hydrolase of GH105; pectate lyases of PL3, PL9, and PL10; pectin lyase of PL1; rhamnogalacturonan lyase of PL4 and PL11; oligogalacturonate lyase of PL22), chitin (putative chitinase of GH18 and GH51), and polygalactosamine (putative endo-α-1,4-polygalactosaminidase of GH114) were also identified (Table 2).

**Table 2:** Transcriptional levels of all CAZyme families when grown on glucose and lignocellulosic biomass substrates

Family	Number of transcripts	Normalized (FPKM) when grown on^a^					Fold change in expression compared to glucose^b^				Putative substrate
		Glucose	Alfalfa	Energy cane	Corn stover	Sorghum	Alfalfa	Energy cane	Corn stover	Sorghum	
*Glycosyl hydrolase families*											
GH1	9	1771.34	3969.55	2979.04	3698.85	3909.06	*1.16*	*0.75*	*1.06*	*1.14*	Cellulose
GH2	1	5.92	8.56	1.30	1.05	4.12	0.53	−***2.19***	−***2.50***	−0.52	Xylan accessory
GH3	17	714.37	673.63	1050.78	3505.03	1394.83	−0.08	0.56	*2.29*	*0.97*	Cellulose
GH4	3	24.53	6.31	12.16	17.96	11.59	−***1.96***	−***1.01***	−0.45	−***1.08***	Other: catabolism
GH5	36	875.19	785.76	950.37	1080.05	844.70	−0.16	0.12	0.30	−0.05	Cellulose
GH6	18	1799.30	3264.96	3861.61	6956.76	4372.47	*0.86*	*1.10*	*1.95*	*1.28*	Cellulose
GH8	2	117.56	32.19	32.93	13.32	111.82	−***1.87***	−***1.84***	−***3.14***	−0.07	Cellulose, hemicellulose
GH9	21	607.91	887.25	1416.88	2192.44	2208.01	0.55	*1.22*	*1.85*	*1.86*	Cellulose
GH10	28	2347.94	2686.49	1930.29	2488.99	3482.82	0.19	−0.28	0.08	0.57	Xylan
GH11	24	1488.52	1007.52	1918.48	1979.64	6240.67	−0.56	0.37	0.41	*2.07*	Xylan
GH13	17	2427.14	337.37	2709.48	2410.50	2781.37	−***2.85***	0.16	−0.01	0.20	Starch
GH16	10	3323.52	1589.54	1295.78	1372.04	1217.24	−1.06	−1.36	−1.28	−1.45	Mixed glucan
GH17	1	0.00	0.00	0.68	1.18	0.69	0.00	*19.38*	*20.17*	*19.40*	Unknown
GH18	12	214.01	319.16	561.21	530.93	330.65	*0.58*	*1.39*	*1.31*	*0.63*	Chitin
GH20	1	2.89	2.07	4.41	5.20	3.29	−0.48	0.61	*0.85*	0.19	Other: *N*-acetyl-β-d-hexosamines
GH25	3	9.17	86.84	52.25	9.90	19.69	*3.24*	*2.51*	0.11	1.10	Peptidoglycan
GH26	7	136.35	167.23	73.65	94.95	46.23	0.29	−***0.89***	−0.52	−***1.56***	Mannan
GH28	5	107.74	67.83	65.97	65.68	85.86	−***0.67***	−***0.71***	−***0.71***	−0.33	Pectin
GH30	3	41.17	31.90	9.74	19.92	20.84	−0.37	−***2.08***	−***1.05***	−***0.98***	Xylan accessory
GH31	17	1730.49	371.79	621.35	1815.81	646.34	−***2.22***	−***1.48***	0.07	−***1.42***	Xylan accessory
GH32	2	26.28	10.46	27.34	14.64	17.18	−***1.33***	0.06	−0.84	−0.61	Other: fructan
GH36	1	3.41	1.85	6.92	8.23	4.80	−0.88	*1.02*	*1.27*	0.49	Other: fructan
GH37	1	12.40	6.41	9.35	8.58	4.14	−0.95	−0.41	−0.53	−1.58	Other: trehalose
GH38	1	3.26	1.06	7.18	4.16	3.38	−***1.62***	*1.14*	0.35	0.05	Other: anabolic
GH39	9	115.54	90.68	93.64	155.76	129.98	−0.35	−0.30	0.43	0.17	Xylan
GH43	32	711.65	822.50	465.65	494.55	747.27	0.21	−0.61	−0.53	0.07	Xylan
GH45	16	2974.32	2653.90	1981.70	1653.87	2315.32	−0.16	−0.59	−***0.85***	−0.36	Cellulose
GH47	3	12.98	5.33	32.56	19.49	14.89	−***1.28***	*1.33*	0.59	0.20	Other: anabolic
GH48	17	3609.25	1928.40	2587.13	3989.36	2077.62	−***0.90***	−0.48	0.14	−***0.80***	Cellulose
GH53	2	24.78	31.13	17.52	27.93	17.37	0.33	−0.50	0.17	−0.51	Pectin
GH55	1	6.91	17.95	10.63	9.13	15.71	*1.38*	0.62	0.40	1.18	β-1,3, Glucan (laminarin)
GH57	7	156.86	540.18	751.41	414.40	377.49	*1.78*	*2.26*	*1.40*	*1.27*	Starch
GH64	2	5.43	17.40	6.22	3.17	2.99	*1.68*	0.20	−***0.78***	−***0.86***	β-1,3, Glucan (laminarin)
GH67	1	0.99	6.39	1.61	2.11	2.92	*2.69*	0.71	*1.10*	*1.57*	Xyloglucan
GH74	4	50.30	94.58	65.58	64.75	31.52	*0.91*	0.38	0.36	−0.67	Xyloglucan
GH78	1	15.39	18.06	4.18	2.60	3.97	0.23	−***1.88***	−***2.56***	−***1.96***	Pectin
GH95	1	35.79	39.09	21.54	16.34	37.22	0.13	−***0.73***	−***1.13***	0.06	Xyloglucan
GH97	2	7.13	1.86	4.94	1.01	5.06	−***1.94***	−0.53	−***2.82***	−0.49	Xyloglucan
GH105	1	31.80	47.57	22.45	14.56	30.55	0.58	−0.50	−***1.13***	−0.06	Pectin
GH109	3	6.74	10.53	23.54	19.34	14.64	*0.64*	*1.80*	*1.52*	*1.12*	Polygalactosamine
GH114	16	70.57	355.00	213.95	121.41	123.15	*2.33*	*1.60*	*0.78*	*0.80*	Other (polygalactosamine)
GH115	4	89.40	27.51	7.26	2.56	6.07	−1.70	−***3.62***	−***5.12***	−***3.88***	Xylan accessory
GH119	20	3049.91	946.89	966.78	820.70	519.50	−***1.69***	−***1.66***	−***1.89***	−***2.55***	Starch
GH124	3	70.07	89.06	117.98	178.06	103.34	0.35	0.75	*1.35*	0.56	Cellulose
*Polysaccharide lyase families*											
PL1	21	154.13	343.14	236.82	140.09	116.49	*1.15*	0.62	−0.14	−0.40	Pectate/pectin lyase
PL2	1	2.24	0.64	4.08	3.90	2.58	−***1.80***	*0.87*	*0.80*	0.21	Pectate lyase, exo-polygalacturonate lyase
PL3	9	46.40	177.00	46.85	75.80	29.12	*1.93*	0.01	0.71	−***0.67***	Pectate lyase
PL4	4	54.08	56.23	77.28	88.88	137.43	0.06	0.52	0.72	*1.35*	Rhamnogalacturonan lyase
PL9	1	9.09	28.83	6.25	3.16	7.53	*1.67*	−0.54	−***1.52***	−0.27	Pectate lyase, exo-polygalacturonate lyase
PL10	1	8.71	15.27	19.57	21.08	11.62	*0.81*	*1.17*	*1.27*	0.42	Pectate lyase
PL11	1	0.96	0.34	1.61	1.59	1.31	−***1.51***	0.74	0.73	0.45	Rhamnogalacturonan lyase
PL22	5	73.10	42.63	32.88	17.93	29.22	−***0.78***	−***1.15***	−***2.03***	−***1.32***	Oligogalacturonate lyase
*Carboxyl esterase families*											
CE1	67	1064.51	1482.74	1331.78	824.56	2043.05	0.48	0.32	−0.37	*0.94*	
CE2	4	18.08	59.94	61.97	84.97	41.02	*1.73*	*1.78*	*2.23*	*1.18*	
CE3	9	190.73	200.04	258.23	170.58	311.61	0.07	0.44	−0.16	0.71	
CE4	66	5195.95	3586.97	4918.19	5519.76	4410.55	−0.53	−0.08	0.09	−0.24	
CE6	10	950.25	208.31	165.10	142.38	367.79	−***2.19***	−***2.52***	−***2.74***	−***1.37***	
CE7	4	428.18	226.89	367.54	378.99	358.44	−***0.92***	−0.22	−0.18	−0.26	
CE8	5	25.32	48.98	84.27	53.33	44.48	*0.95*	*1.73*	*1.07*	*0.81*	
CE9	3	0.74	4.64	3.44	1.35	1.33	*2.64*	*2.21*	*0.86*	*0.84*	
CE10	58	559.63	576.67	758.11	433.52	332.26	0.04	0.44	−0.37	−***0.75***	
CE12	4	133.25	137.86	137.67	201.83	175.85	0.05	0.05	0.60	0.40	
CE14	1	1.17	1.91	5.03	2.62	2.64	0.71	*2.10*	*1.16*	*1.18*	
CE15	2	259.98	238.38	105.97	41.58	240.61	−0.13	−***1.29***	−***2.64***	−0.11	
CE16	12	125.10	160.06	118.64	47.91	156.38	0.36	−0.08	−***1.38***	0.32	
CEX	7	906.91	381.49	426.72	481.87	779.19	−***1.25***	−***1.09***	−***0.91***	−0.22	

^a^Corrected FPKM values normalized by the library size, as calculated using the estimateDispersions function in the R package DESeq [44]

^b^Fold change is shown as Log_2_ expression levels compared to glucose. Italics represents significantly over-expressed (a differential expression *p* value <0.01 as calculated by the nbinomTest function in the R package DESeq), bold italics represents significantly under-expressed (a differential expression *p* value <0.01 as calculated by the nbinomTest function in the R package DESeq [44]

### Transcriptional patterns of CAZymes in strain C1A at the family and transcript levels

**Fig. 1:**
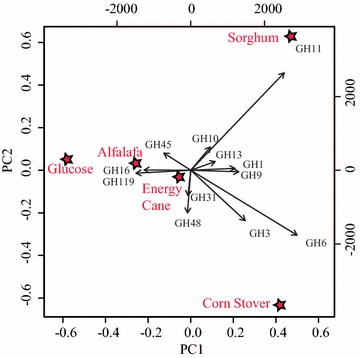
Principal component analysis (PCA) of normalized transcription levels of GH families. Normalized FPKM values of GH families under different growth conditions were used as input. *Stars* represent growth substrates and arrows represent GH families. Growth substrates with similar transcriptional profiles are closer together in the ordination plot than substrates with different transcriptional profiles. The direction of arrows in the biplot is indicative of the respective maximal transcription, while the length of the *arrows* is indicative of the differential transcription

We analyzed the transcriptional patterns of CAZymes in strain C1A at the family and transcript levels. When grown on glucose, strain C1A constitutively transcribes a relatively high baseline level of CAZyme (GHs, CEs, but not PLs) transcripts that include a wide range of cellulolytic, hemicellulolytic, amylolytic, and accessory enzymes (Table 2). Indeed, many of the CAZyme families were transcribed at levels comparable to, or even exceeding, those of key glycolytic enzymes such as pyruvate kinase (normalized FPKM of 115.2), Fructose-bisphosphate aldolase (normalized FPKM value 1563.5), and even in few cases (e.g., GH45 endoglucanase, GH48 cellobiohydrolase, and GH119 α-amylase) glyceraldehyde-3-P dehydrogenase (normalized FPKM of 2970.4) (Table 2, Additional file 1: Tables S1 and S2). Growth on lignocellulosic biomass was associated with few distinct changes in transcriptional levels of several GH families (Fig. 1; Table 2). In total, 6 GH families (GH1, GH6, GH18, GH57, GH109, and GH114) were significantly (*p* value <0.01) upregulated, while one (GH119) was significantly (*p* value <0.01) downregulated across all four lignocellulosic biomass growth conditions. In addition, few families (GH9, GH25, GH55, GH67, and GH124) showed increased (higher normalized FPKM values) or decreased (lower normalized FPKM values, GH4, GH8, GH28, GH30, GH37, GH45, GH97, and GH115) transcriptional levels in all examined growth conditions, although this change was statistically significant (*p* value <0.01) only in some, but not all, growth conditions examined.

Within highly transcribed GH families putatively involved in cellulose degradation (GH1, GH3, GH5, GH6, GH9, GH45, and GH48, defined using a normalized FPKM cutoff value > pyruvate kinase, the glycolytic gene with the lowest transcriptional level under all growth conditions), only one putative cellobiohydrolase (GH6) and one putative β-glucosidase (GH1) families were significantly upregulated in all plant biomass conditions compared to glucose. One putative endoglucanase family (GH9) showed higher transcriptional levels on all plant biomass conditions, although this upregulation was significant (*p* value <0.01) only in three (energy cane, corn stover, and sorghum) out of four examined growth conditions. One the other hand, GH48 cellobiohydrolases were significantly downregulated in alfalfa- and sorghum-grown cultures compared to glucose (Fig. 1; Table 2).

While few, yet distinct, differential regulation patterns were observed in cellulolytic GH families, no clear family wide up- or downregulation patterns were observed in xylanolytic families. Transcriptional levels of the GH10 putative xylanases and GH39 and GH43 putative xylosidases did not show any statistically significant difference when comparing all four plant biomass conditions, compared to glucose (Fig. 1; Table 2). Within GH11 xylanases, significant upregulation was observed only in sorghum-grown cultures compared to glucose-grown cultures (Fig. 1; Table 2). Collectively, these results suggest that strain C1A constitutively transcribes high level of lignocellulosic enzyme transcripts, even in the absence of lignocellulosic substrates, with growth on lignocellulosic biomass with the substrate eliciting few distinct changes in transcriptional patterns of specific GH families (Fig. 1; Table 1). This overall pattern of transcriptional change, or lack thereof, is quite distinct from the scheme utilized by aerobic lignocellulolytic fungi (e.g., *Aspergillus niger* and *Trichoderma reesei* [24, 25]), where growth on lignocellulosic biomass causes a drastic induction of cellulolytic and lignocellulolytic enzymes from low, almost undetectable transcriptional levels on glucose. However, this pattern is broadly similar to transcriptomic response observed in anaerobic lignocellulolytic bacteria (e.g., *Clostridium phytofermentans*, *C. cellulolyticum*, *C. thermocellum* [26–28]), which grow and express their CAZymes on glucose as well as lignocellulolytic biomass.

On a single-transcript level, 39 (energy cane) to 48 (alfalfa) GH transcripts were significantly (*p* < 0.01) upregulated in biomass-grown versus glucose-grown cultures, while a broadly comparable number of transcripts (53 sorghum–66 corn stover) were significantly downregulated. The majority of transcripts (192 in corn stover and energy cane, and 210 in alfalfa and sorghum), however, did not show a significant change in transcription levels (*p* > 0.1) (Table 3, Additional file 1: Table S1). A similar pattern was also observed for CE and PL families as well (Table 3, Additional file 1: Table S1).

**Table 3:** Transcriptional patterns of C1A CAZymes on various substrates

Transcripts	Number of transcripts when grown on			
	Alfalfa	Energy cane	Corn stover	Sorghum
*Glycosyl hydrolases*				
Significantly upregulated^a^	48	39	43	46
Upregulated^b^	37	51	44	36
Downregulated^c^	36	42	40	40
Significantly downregulated^d^	54	61	66	53
No change^e^	210	192	192	210
*Polysaccharide lyases*				
Significantly upregulated^a^	10	5	3	4
Upregulated^b^	6	2	4	2
Downregulated^c^	2	4	3	4
Significantly downregulated^d^	3	1	5	5
No change^e^	19	28	26	25
*Carbohydrate esterases*				
Significantly upregulated^a^	55	44	47	45
Upregulated^b^	18	29	24	17
Downregulated^c^	28	26	23	29
Significantly downregulated^d^	35	31	40	22
No change^e^	114	119	116	137

^a^Significantly upregulated refers to the number of transcripts with a differential expression *p* value <0.01 as calculated by the nbinomTest function in the R package DESeq [44]

^b^Upregulated refers to the number of transcripts with a differential expression *p* value between 0.01 and 0.1 as calculated by the nbinomTest function in the R package DESeq [44]

^c^Downregulated refers to the number of transcripts with a differential expression *p* value <0.01 as calculated by the nbinomTest function in the R package DESeq [44]

^d^Significantly downregulated refers to the number of transcripts with a differential expression *p* value between 0.01 and 0.1 as calculated by the nbinomTest function in the R package DESeq [44]

^e^No change refers to the number of transcripts with a differential expression *p* value >0.1 as calculated by the nbinomTest function in the R package DESeq [44]

We also correlated transcriptional levels of various GH families with the composition (cellulose and hemicellulose content, Additional file 1: Table S3) of plant materials examined as growth substrates in this study. Transcriptional levels of some cellulolytic CAZyme families, e.g., GH5, GH6, GH9, GH48, and GH124, were positively correlated (Pearson correlation coefficients of 0.42, 0.81, 0.71, 0.58, and 0.62, respectively) with the substrates’ cellulose content (i.e., overall normalized FPKM of the family was higher in plants with higher cellulose content). However, no such correlation was observed for GH8 or GH45 (Pearson correlation coefficients of 0.06 and −0.36, respectively). On the other hand, no clear correlation was observed between transcriptional levels of xylanase CAZyme families (GH10 and GH11) and hemicellulose content (Pearson correlation coefficient of −0.32 and −0.19, respectively). GH39 xylosidase showed a positive correlation with hemicellulose content (Pearson correlation coefficient of 0.60), while GH43 xylosidase showed a strong negative correlation with hemicellulose content (Pearson correlation coefficient of −0.93).

### Strain C1A employs multiple functionally redundant but structurally and mechanistically distinct processes for biomass degradation

**Fig. 2:**
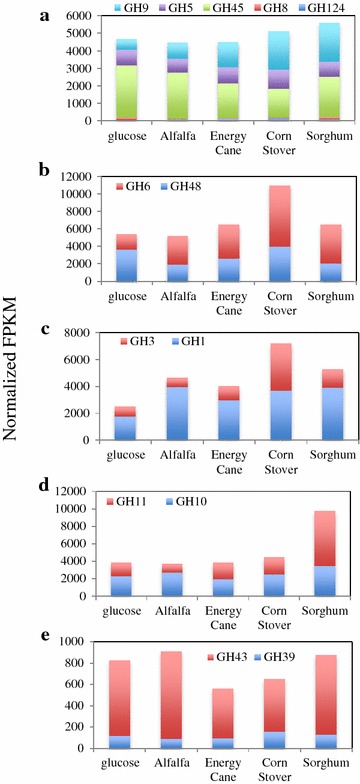
Relative contribution of various GH families putatively mediating key enzymatic activities required for cellulose and xylan degradation under different growth conditions. **a** Endoglucanases, **b** Cellobiohdrolases, **c** β-glucosidases, **d** Xylanases, **e** β-Xylosidases

To examine the relative contribution of various CAZyme families to biomass degradation under different growth conditions, we quantified the relative transcriptional levels of families putatively mediating the deconstruction of various plant polymers as a fraction of an overall specific activity. Our results (Fig. 2, Additional file 1: Table S4) demonstrate that strain C1A co-transcribes multiple functionally redundant enzymes (i.e., mediating the exact same chemical reaction and targeting the same substrate) that are, nevertheless, mechanistically and structurally distinct. While the identification of many of these genes in anaerobic fungi has been previously documented [22, 29], their differential transcriptional patterns and relative contribution to biomass degradation under various growth conditions have not been previously studied. For example, transcripts of putative endoglucanases belonging to five distinct families were identified, three of which [the (α/β)_8_ TIM barrel retaining GH5, the (α/α)_6_ barrel inverting GH9, and the β barrel inverting GH45) represented >15 % of overall endoglucanases under all growth conditions (Fig. 2a]. A similar high level of co-transcription of the inverting α/β barrel GH6 putative cellobiohydrolase acting on the non-reducing end of cellulose molecules and the retaining (α/β)_8_ TIM barrel putative cellobiohydrolase acting on the reducing end of the cellulose molecule was observed (Fig. 2b). Finally, a high co-transcriptional level of GH1 and GH3 putative β-glucosidases was also observed (Fig. 2c). Within putative xylanolytic enzymes, a similar phenomenon is observed between the retaining (α/β)_8_ TIM barrel GH10 putative xylanase and the retaining β-jelly roll GH11 (Fig. 2d), and the same dynamic was observed between putative xylosidases (GH39 and GH43) mediating depolymerization of xylooligomers (Fig. 2e).

**Fig. 3:**
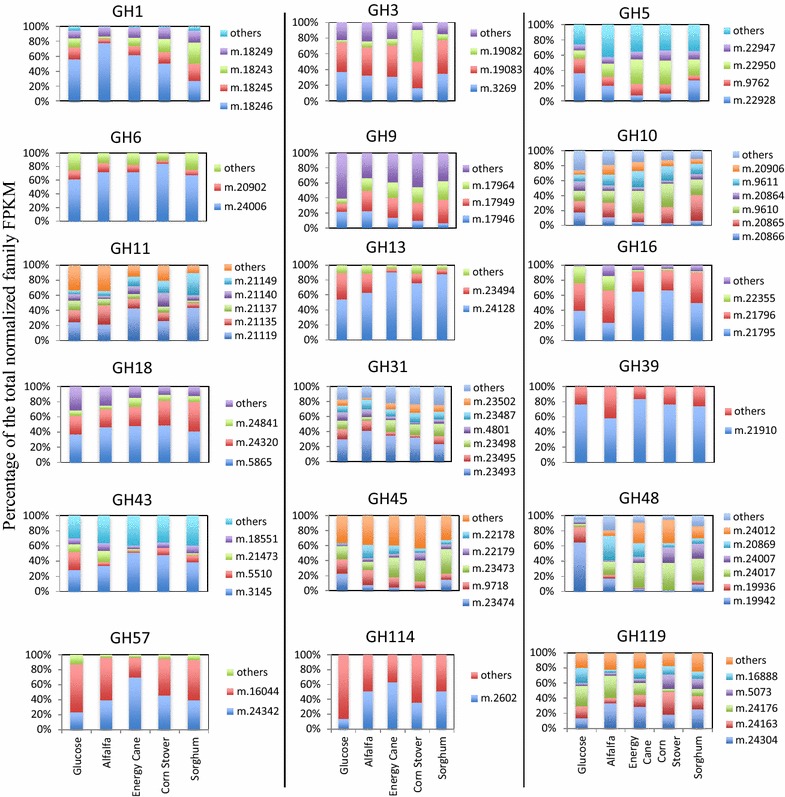
Relative contribution of dominant transcripts within various GH families under different growth conditions. Only families with overall transcriptional level under all growth conditions above 1 % that of a suite of glycolytic genes (pyruvate kinase, glyceraldehyde-3-phosphate dehydrogenase, and fructose-1,6-bisphosphate aldolase) were studied. Within these, genes were selected that represented 10 % or more of the overall moralized FPKM under any growth condition. “Others” denotes all additional transcripts that never exceeded >10 % of overall moralized FPKM under any growth condition

Interestingly, distinct shifts in the relative transcript abundances of GH families as a fraction of an overall specific activity were frequently observed (Fig. 2). Within glucose-grown cultures, the majority of putative endoglucanases belonged to GH45 (65 % of putative endoglucanases normalized FPKM in glucose-grown cultures). However, when grown on plant biomass, the relative abundance of GH45 decreased, with a concomitant increase in the relative abundance of GH9 putative endoglucanases (Fig. 3a). Similarly, growth on plant biomass was invariably associated with an increase in the relative contribution of GH6 and a reciprocal decrease in the relative contribution of GH48 to the overall cellobiohydrolase activity (Fig. 3b).

### A limited number of lignocellulolytic transcripts are highly transcribed under all growth conditions

**Fig. 4:**
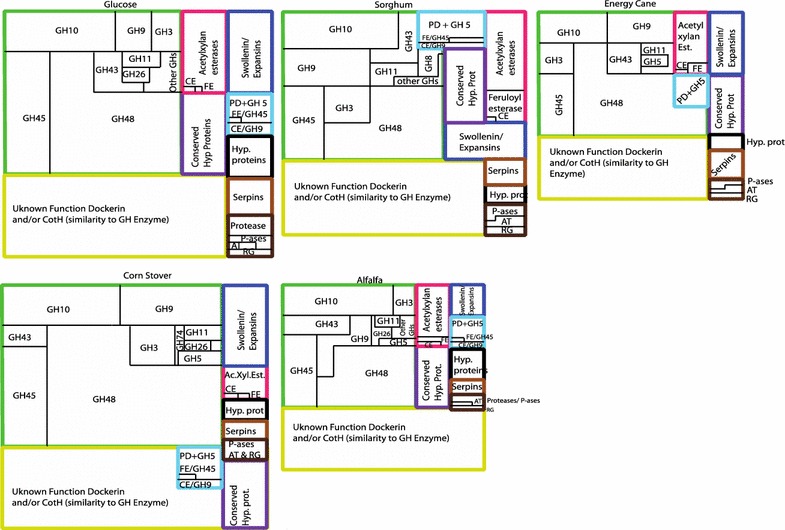
FDD-containing, putatively cellulosomal transcripts. *Each square* depicts transcriptional patterns under a specific growth condition as shown *above* the *squares*. The size of *each square*, and sections within, is proportional to the transcriptional level (normalized FPKM values). The sections are color coded by their predicted activity as follows: *green* GH families; *dark blue* swollenin/expansin accessory enzymes; *pink* acetylxylan esterases, carboxyl esterases (CE), and feruloyl esterases (FE); *black* hypothetical proteins; *purple* conserved hypothetical proteins; *brown* protease inhibitors (serpins); *dark brown* serine and threonine protein phosphatases (P-ases), alkyl transferases (AT), proteases, and rhamnogalacturonases (RG); *light blue* dual-activity enzymes including polysaccharide deacetylases (PD)/GH5 proteins, feruloyl esterases (FE)/GH45 proteins, and carboxyl esterases (CE)/GH9 proteins

Within a single CAZyme gene family, often a large number of distinct transcripts were identified, and this was especially true for families with a high overall transcriptional activity (Table 2). Indeed, a broad positive correlation between the total FPKM level of a specific GH family and the number of transcripts identified belonging to this family was observed (Additional file 1: Figure S2). To further zoom in on the putative variations in the contribution of specific transcripts belonging to a certain GH family to biomass degradation, we examined the transcriptional levels of all individual transcripts within key GH families. Out of the large number of transcripts identified in each family (Additional file 1: Table S1), a fairly limited (1–6) number of transcripts were dominant (i.e., represent >10 % of the total normalized family FPKM under at least one growth condition) in all instances (Fig. 4). Transcriptional patterns of dominant transcripts under different growth conditions varied across different CAZyme families. In some families (e.g., GH6, GH13, and GH39), a single transcript represented the majority (>60 %) of all FPKM levels across all growth conditions. In other instances, few (2–3) transcripts consistently represented the majority of family transcripts, with their relative abundance patterns remaining fairly stable across various growth conditions (e.g., GH18, GH43, and GH57). Within the remaining families, a significant shift in the relative transcriptional level, and hence putative contribution, was observed between different growth conditions. For example, specific transcripts in GH5 (m.22928), GH13 (m.23494), GH43 (m.5510), GH45 (m.23474), and GH48 (m.19942) appear to be highly transcribed in glucose-grown cultures, but their relative importance diminishes in lignocellulosic biomass-grown cultures. Conversely, some transcripts appear to be prominent and differentially upregulated in lignocellulosic biomass-grown cultures, while their contribution to the overall activity dwindles in glucose-grown cultures (e.g., m.17949 and m.17964 in GH9, m.20865 in GH10, m.21149 in GH11, and m.23473 in GH45). Collectively, the results demonstrate that while some families show differential transcriptional patterns in response to growth conditions, a few stable “core” of transcripts, especially within highly transcribed CAZyme families in strain C1A, appears to be consistently predominant.

### Fungal dockerin domain (FDD)-containing transcripts

Anaerobic fungi produce cellulosomes with surface-attached structures where multiple enzymes act synergistically toward the degradation of lignocellulosic biomass. As previously described, cellulosome-bound genes in anaerobic fungi usually harbor a fungal dockerin domain (FDD) that is similar in structure to carbohydrate-binding module family 10 (CBM10) [12]. By determining FDD occurrence in all transcripts, a total of 278, 283, 292, 288, and 291 were putatively identified as “cellulosome-bound transcripts” in glucose-, alfalfa-, energy cane-, corn stover-, and sorghum-grown C1A cultures, respectively (Additional file 1: Table S5), with the absolute majority of transcripts identified in all examined growth conditions. Cellulosome-bound transcripts were affiliated with 4 broad major categories: biomass-degrading CAZymes and accessory enzymes; hypothetical and conserved hypothetical proteins; proteases, phosphohydrolases, and protease inhibitors (serpins); and the enigmatic CotH family protein transcripts previously observed in fungal and bacterial cellulosomes and previously implicated as a structural component of the cellulosome [30]. Analysis of the transcriptional patterns of FDD transcripts under different growth conditions indicated that the relative contribution of the four major categories described above to the overall cellulosome composition did not vary significantly when C1A was grown on glucose versus plant biomass (likelihood ratio *χ*^2^ = 59.88, *p* value = 0.055).

Examination of FDD CAZyme and accessory transcripts (Additional file 1: Table S5) suggests the involvement of the cellulosome in all stages of cellulose (putative endoglucanases, cellobiohydrolases, and β-glucosidases), arabinoglucoxylan (putative xylanases, xylosidases, arabinofuranosidases, acetylxylan esterase, and feruloyl esterases), xyloglucan (xyloglucanases), and glucomannan (putative mannanases/mannosidases) degradation. Within a specific GH family, the relative contributions of FDD transcripts to the overall family transcriptional level varied (Table 4). Based on the number of transcripts and transcriptional activity, FDD transcripts represent the absolute majority of transcriptional activity in GH48 putative cellobiohydrolases, the majority in GH5 putative endoglucanases, roughly half the transcriptional activity in GH9 putative endoglucanases, GH10 putative xylanases, and GH43 putative β-xylosidases, and a small fraction of the transcriptional activities of GH11 putative xylanases and GH45 putative endoglucanases (Table 4). Interestingly, overall expression of GH and accessory enzyme transcripts was significantly downregulated in three (alfalfa, energy cane, and sorghum) growth conditions (Additional file 1: Table S6), mainly due to the significant downregulation of GH48, a major component of the cellulosome, under these growth conditions (Table 2, Additional file 1: Table S6). Other notable contributions of the putatively cellulosome-bound, FDD-harboring transcripts to biomass degradation include the prevalence of carbohydrate esterases (3.5–5.7 % of overall FDD transcripts, depending on the growth condition) and feruloyl esterases (up to 3.8 % of overall FDD transcripts) within all FDD-harboring transcripts (Table 4), suggesting an important role of the cellulosome in the mobilization and debranching of hemicellulose backbones. In addition to CAZyme families responsible for cell wall decomposition, an important accessory transcript belonging to the swollenin/expansin enzyme family was identified as cellulosome bound. This enzyme family enables plant cell lengthening through non-catalytic disruption of hydrogen bonds in plant cell walls [31]. Homologs of this enzyme family have also been shown to enhance cell wall decomposition when utilized by microorganisms [32]. Out of the five swollenin/expansin transcripts identified, four contained an FDD and represented 89–97 % of total normalized FPKM activity, depending on the growth condition, of total swollenin transcripts identified in C1A transcriptome (Table 4). Although swollenin and GH45 are structurally related [33], the predominant cellulosomal transcriptional pattern of the non-enzymatic swollenin is in contrast to that observed mostly free extracellular patterns of GH45 transcripts. The predominance of this non-catalytic homolog in the cellulosome emphasizes their important role in cell wall weakening as an additional mechanism to enhance plant biomass degradation efficiency by cellulosomal catalytic enzymes.

**Table 4:** Glycosyl hydrolase (GH) transcripts in C1A cellulosome under different growth conditions

CAZyme family	Grown on	Number of genes		Total expression level (corrected FKPM)^a^		Cellulosomal expression as the percentage of overall expression
		Cellulosomal transcripts	Non-cellulosomal transcripts	Cellulosomal transcripts	Non-cellulosomal transcripts	
GH2	Glucose	1	1	5.92	0	100
	Alfalfa	1	1	8.56	0	100
	Energy cane	1	1	1.3	0	100
	Corn stover	1	1	1.05	0	100
	Sorghum	1	1	4.12	0	100
GH3	Glucose	2	17	314.9	399.47	44.08
	Alfalfa	2	17	242.54	431.09	36.01
	Energy cane	2	17	351.98	698.8	33.5
	Corn stover	2	16	652.1	2852.93	18.6
	Sorghum	2	17	446.42	948.41	32.01
GH5	Glucose	17	30	726.01	149.18	82.95
	Alfalfa	16	34	483.4	302.36	61.52
	Energy cane	15	34	607.37	343	63.91
	Corn stover	16	34	804.65	275.4	74.5
	Sorghum	19	34	556.98	287.72	65.94
GH6	Glucose	5	17	238.21	1561.09	13.24
	Alfalfa	6	18	281.87	2983.09	8.63
	Energy cane	6	18	495.5	3366.11	12.83
	Corn stover	6	18	615.54	6341.22	8.85
	Sorghum	6	18	473.62	3898.85	10.83
GH8	Glucose	1	2	113.21	4.35	96.3
	Alfalfa	1	2	30.34	1.85	94.25
	Energy cane	1	2	30.81	2.12	93.56
	Corn stover	1	2	13	0.32	97.6
	Sorghum	1		99.22	12.6	88.73
GH9	Glucose	9	21	340.63	267.28	56.03
	Alfalfa	9	21	529.3	357.95	59.66
	Energy cane	9	20	686.42	730.46	48.45
	Corn stover	9	20	1124.22	1068.22	51.28
	Sorghum	9	20	931.39	1276.62	42.18
GH10	Glucose	9	28	1339.11	1008.83	57.03
	Alfalfa	9	28	1121.04	1565.45	41.73
	Energy cane	9	28	927.74	1002.55	48.06
	Corn stover	9	28	1300.71	1188.28	52.26
	Sorghum	9	28	1167.53	2315.29	33.52
GH11	Glucose	5	23	194.91	1293.61	13.09
	Alfalfa	5	23	96.62	910.9	9.59
	Energy cane	5	24	110.25	1808.23	5.75
	Corn stover	5	23	171.18	1808.46	8.65
	Sorghum	5	24	175.12	6065.55	2.81
GH26	Glucose	5	6	136	0.35	99.74
	Alfalfa	4	7	127.33	39.9	76.14
	Energy cane	4	7	56.67	16.98	76.94
	Corn stover	4	7	88	6.95	92.68
	Sorghum	4	7	35.69	10.54	77.19
GH43	Glucose	4	31	318.72	392.93	44.79
	Alfalfa	4	31	433.95	388.55	52.76
	Energy cane	4	32	251.13	214.52	53.93
	Corn stover	4	31	285.36	209.19	57.7
	Sorghum	4	32	298.46	448.81	39.94
GH45	Glucose	6	16	602.8	2371.52	20.27
	Alfalfa	6	16	377.34	2276.56	14.22
	Energy cane	6	16	322.7	1659	16.28
	Corn stover	6	16	420.71	1233.16	25.44
	Sorghum	8	16	343.55	1971.77	14.84
GH48	Glucose	15	17	3094.01	515.24	85.72
	Alfalfa	14	16	1715.18	213.22	88.94
	Energy cane	14	16	2345.99	241.14	90.68
	Corn stover	14	16	3980	9.36	99.77
	Sorghum	16	16	1622.43	455.19	78.09
GH74	Glucose	3	4	50	0.3	99.4
	Alfalfa	3	4	87.66	6.92	92.68
	Energy cane	3	4	53.55	12.03	81.66
	Corn stover	3	4	59.07	5.68	91.23
	Sorghum	3	4	23.3	8.22	73.92
GH95	Glucose	1	1	35.79	0	100
	Alfalfa	1	1	39.09	0	100
	Energy cane	1	1	21.54	0	100
	Corn stover	1	1	16.34	0	100
	Sorghum	1	1	37.22	0	100
GH115	Glucose	1	4	89	0.4	99.55
	Alfalfa	1	4	21.09	6.42	76.66
	Energy cane	1	4	6.09	1.17	83.83
	Corn stover	1	4	2.55	0.01	99.72
	Sorghum	1	4	4.41	1.66	72.7
GH124	Glucose	1	3	35.19	34.88	50.22
	Alfalfa	1	3	41.2	47.86	46.26
	Energy cane	1	3	56.3	61.68	47.72
	Corn stover	1	3	98.51	79.55	55.33
	Sorghum	1	3	43.06	60.28	41.67

^a^Corrected FPKM values normalized by the library size, as calculated using the estimateDispersions function in the R package DESeq [44]

## Discussion

In this study, we analyzed transcriptional patterns in strain C1A when grown on plant biomass as well as soluble (glucose) substrates. Collectively, our results suggest that strain C1A constitutively transcribes a wide array of FDD-containing (i.e., putatively cellulosome-bound) and free extracellular lignocellulolytic enzymes under all examined conditions. The results also highlight the simultaneous involvement of multiple functionally redundant CAZymes in plant biomass degradation, arguably as a tool to improve the speed and extent of biomass degradation by anaerobic fungi within its natural habitat (the herbivorous gut). Finally, the results provide an in-depth evaluation of the contribution of free versus FDD-containing (i.e., putatively cellulosome-bound) enzymes in biomass degradation in strain C1A.

Our results demonstrate that strain C1A constitutively transcribes a wide array of transcripts encoding lignocellulolytic enzymes (Table 2, Additional file 1: Tables S1, S2, Figure S2). Microorganisms growing on lignocellulosic biomass invariably spend a large fraction of their carbon and energy reserves on the synthesis and export of lignocellulolytic enzymes (CAZymes). Therefore, regulation of the biosynthesis of such enzymes is key for optimal ecological fitness and resource allocation. Within model lignocellulolytic aerobic fungi, e.g., *A. niger* and *T. reesei*, growth on lignocellulosic biomass causes a drastic induction of cellulolytic and lignocellulolytic enzymes from almost undetectable transcriptional levels on glucose-grown cultures, to ≈12–20 % of the overall mRNA [24, 25]. This induction pattern is associated with a drastic change in the relative composition of the CAZyme transcriptome from a glucoamylase-dominated profile when grown on glucose or other soluble substrates to an endoglucanase-, cellobiohydrolase-, xylanase-, arabinofuranosidase-, acetylxylan esterase-, and polysaccharide monooxygenase-dominated profile when grown on lignocellulosic biomass [24, 25]. On the other hand, multiple anaerobic prokaryotes (e.g., *Clostridium cellulolyticum*, *C. phytofermentans*, and *C. thermocellum*) possess constitutively expressed CAZymes and high overall transcriptional levels of lignocellulolytic enzymes are observed in glucose-grown cultures [26–28]. Indeed, it is postulated that glucose sensing appears to act as a priming mechanism that stimulates biosynthesis of a wide range of CAZymes [26–28]. Our results suggest that anaerobic fungi employ a model similar to anaerobic bacteria as opposed to aerobic fungi. This conclusion is in accordance with our understanding of the ecological niche and life cycle of anaerobic fungi within its restricted habitat in the herbivorous gut. In such an environment, the life cycle of anaerobic fungi alternates between metabolically dormant spores and hyphae germinating from spores when ingested plant biomass is encountered in the gut. Fungal germination and growth is hence invariably linked to the availability of ingested plant biomass. Therefore, spore germination, hyphal growth, and production of lignocellulolytic enzymes in anaerobic fungi are tightly linked, and it is inconceivable to envision a situation in which anaerobic fungi grow solely on a soluble substrate within their natural habitat. Therefore we argue that, due to their ecological niche, their role as initial colonizers of plant biomass, and their sole dependence on plant biomass as a substrate within their natural habitat, the need for development of sophisticated mechanisms for regulating the expression of CAZyme genes is non-existent in anaerobic fungi. This is drastically different from what is encountered by aerobic lignocellulolytic fungi in their natural environments, where gradients in environmental conditions (temperature, pH, moisture), substrate availability (by season) and type (plant biomass vs sugars), and the relatively large residence time and degradation rates necessitate the development of regulatory processes for enzymatic biosynthesis. Nevertheless, despite this constitutive pattern of CAZyme gene transcription in anaerobic fungi it appears that growth on plant biomass triggers a distinct response in CAZyme GH families and individual transcripts (Table 2; Figs. 2, 3). The rationale behind these family and transcript level shifts, observed mainly within GH families and transcripts involved mainly in various aspects of cellulose degradation, remains unclear.

Another interesting characteristic in lignocellulosic biomass degradation by strain C1A is the simultaneous engagement of a large number of functionally redundant enzymes in the degradation of a single polymer (e.g., cellulose or arabinoxylan). We argue that this strategy is employed by C1A to increase the efficacy and speed of the degradation process, and hence maximize the extent of plant biomass degraded within its relatively short residence time in the herbivorous gut. Further, the complementary nature of this strategy is further accentuated by variations in the location of the enzymes (cellulosomal vs free extracellular), the nature of the substrate targeted (chain length and side chains preferences), and the target position (e.g., reducing vs non-reducing end) within the substrate. Transcripts encoding most enzymatic activities required for the degradation of cellulose and hemicellulose are well represented in both putatively cellulosomal and non-cellulosomal fractions, allowing for the simultaneous degradation of these polymers at two distinct locations. Strain C1A simultaneously transcribes high levels of GH10 and GH11 family transcripts. GH10 enzymes are known to have broader substrate specificity, with the capability to attack xylan backbones with a high degree of substitutions and smaller xylo-oligosaccharides [34]. Therefore, such a pattern of high co-transcription allows for the instant and sustained breakdown of xylan backbone polymer regardless of their length and progress in side chain removal by accessory enzymes. Finally, the co-transcription of GH6 and GH48 cellobiohydrolases by C1A allows for the simultaneous targeting of reducing ends of both celluloses and cellooligosaccharides in plant biomass to improve speed and efficiency of cellulose degradation.

Third, our results highlight the importance of anaerobic fungal cellulosomes for biomass degradation. While broad upregulation in FDD transcripts was observed in plant biomass-grown versus glucose-grown cultures, no drastic changes in membership (presence/absence) of specific transcripts or composition (relative levels of specific transcripts) were observed. The results suggest that cellulosome structure does not vary considerably depending on the growth substrate, as previously suggested. Further, FDD transcripts identified strongly suggest that cellulosomes represent distinct catalytic units capable of independently attacking and converting intact plant fibers to sugar monomers. A large number of highly transcribed transcripts are involved in the initial disruption of plant fiber architecture through non-catalytic hydrolysis of hydrogen bonds (swollenin), mobilization of target plant polymers (feruloyl esterases), side chain removal (acetylxylan esterase, polysaccharide deacetylase), and degradation of plant polymers into sugar monomers (endoglucanases, cellobiohydrolases, β-glucosidases; xylanases and xylosidases).

## Conclusions

Our work demonstrates that strain C1A constitutively transcribes a wide array of lignocellulolytic enzymes under different growth conditions. Although many of these enzymes are functionally redundant, differences in location (cellulosomal vs free extracellular), substrate preference (polymer length and substitution patterns), and target position within the substrate (e.g., reducing vs non-reducing end) allow for fast and efficient utilization of target substrates in the relatively short time frame of availability within the herbivorous gut. The utilization of this indiscriminate strategy as an ecological and evolutionary necessity, as well as capability of anaerobic fungi to utilize a broad range of plant biomass including lignocellulosic biomass substrates, renders anaerobic fungi appealing, yet understudied, candidates for utilization in biomass conversion to sugars and biofuels.

## Methods

### *Orpinomyces* sp. strain C1A

Strain C1A was isolated from the feces of an Angus steer in our laboratory on a cellobiose–switchgrass medium as described previously [22]. Strain C1A is maintained by biweekly subculture on a cellobiose–rumen fluid medium as described previously [35].

### Plant biomass

Samples of mature Sorghum (*Sorghum bicolor*) and mature energy cane (*Saccharum officinarum* var. Ho02) were obtained from Oklahoma State University experimental plots in Stillwater, OK. Dried alfalfa (*Medicago sativa*) was obtained from a local farm and ranch supplier. Samples of corn stover from *Zea mays* were obtained from the Industrial Agricultural Products Center at the University of Nebraska in Lincoln. The composition of all substrates is listed in Additional file 1: Table S5.

### Experimental setup

All transcriptomic experiments were conducted in a rumen fluid-free basal medium containing (g L^−1^) 0.5 g yeast extract, 0.47 g sodium butyrate, 2.4 g sodium acetate, 0.8 g sodium propionate, 2 g tryptone, 2 ml hemin solution (5 g L^−1^ in 1 M NaOH), 9.3 ml fatty acid solution (composition ml L^−1^: 11.7 ml isobutyric acid, 11.7 ml valeric acid, 11. 7 ml isovaleric acid, and 11.7 ml methylbutyric acid), 150 ml mineral solution I (3 g L^−1^ K_2_HPO_4_), 150 ml mineral solution II (composition g L^−1^: 3 g KH_2_PO_4_, 6 g (NH_4_)_2_SO_4_, 6 g NaCL, 0.6 g MgSO_4_·7H_2_O, 0.6 g CaCl_2_·2H_2_O), 10 ml Balch Vitamin solution (composition mg L^−1^: 2 mg biotin, 2 mg folic acid, 10 mg pyridoxine–HCl, 5 mg thiamine–HCl, 5 mg riboflavin, 5 mg nicotinic acid, 5 mg DL calcium pantothenate, 0.1 mg vitamin B12, 5 mg PABA, 5 mg lipoic acid), and 1 ml Wolin’s metal solution (composition g L^−1^: 0.5 g EDTA, 3 g MgSO_4_·7H_2_O, 0.5 g MnSO_4_·H_2_O, 1 g NaCl, 0.1 g CaCl_2_·2H_2_O, 0.1 g FeSO_4_·7H_2_O, 0.1 g ZnSO_4_·7H_2_O, 0.01 g CuSO_4_·7H_2_O, 0.01 g AlK(SO_4_), 0.01 g Na_2_MoO_4_·2H_2_O, 0.01 g boric acid, 0.005 g Na_2_SeO_4_, 0.003 g NiCl_2_·6H_2_O, 0.1 g CoCl_2_·6H_2_O). After the medium was prepared, the pH was adjusted to 6.6. The medium was then dispensed under strictly anaerobic conditions as previously described [36, 37]. After the medium was dispensed, sodium carbonate (6 g L^−1^) was added and the bottles were stoppered, sealed, and autoclaved at 121 °C for 20 min. After autoclaving, the bottles were cooled to room temperature. Bottles that were amended with plant materials were moved to an anaerobic glove bag (Coy Laboratory Products Grass Lake, MI), where the appropriate type of plant biomass (10 g L^−1^) was added. The bottles were then stoppered, sealed, and removed from the glove bag, and the headspace was replaced by repeated vacuuming and repressurization with 100 % CO_2_ (insert Balch reference). Bottles that contained glucose were amended with 3.75 g L^−1^ of an anaerobic, sterile stock solution. All experiments that were conducted with plant biomass and glucose were performed in duplicate. The inoculum source for these experiments consisted of strain C1A that was grown in a rumen fluid-free cellobiose medium (same composition as above with the addition of 10 g L^−1^ cellobiose as the carbon source) until late log/early stationary phase. The inoculum was then centrifuged and resuspended in 20 ml of basal media with no carbon source. The experiment was started by adding this slurry of basal medium and fungal biomass (approximately 48 mg) into the appropriate bottles described above.

### RNA extraction and sequencing

RNA extraction was conducted on late log phase cultures after 48 h of inoculation. Fungal biomass was harvested by vacuum filtration and ground into fine particles with a pestle under liquid nitrogen as previously described [35]. Total cellular RNA was extracted from ground fungal biomass using Epicentre MasterPure Yeast RNA Purification kit (Epicentre, Madison, WI, USA), stored in the provided RNase-free TE buffer, and quantified using Qubit fluorometer (Life Technologies, Carlsbad, CA, USA).

RNA-Seq analysis [38] was conducted using the HiSeq 2000 platform with 125 × 2 paired-end read chemistry at the University of Georgia Genomics Facility (Athens, GA, USA). Biological replicate sequencing libraries for all conditions (glucose, corn stover, sorghum, energy cane, and alfalfa) were created with poly-A tailed mRNA enrichment using the standard Illumina TruSeq mRNA RNA-Seq protocol (http://www.utsouthwestern.edu/labs/next-generation-sequencing-core/assets/truseq-stranded-mrna-sample-prep-guide.pdf). The sequencing libraries had an average insert size of approximately of ~300 bp.

### Transcriptome assembly and RNA-Seq quantification

To represent all biological isoforms present in various growth conditions, the generated Illumina sequencing RNA-Seq [38] reads were assembled [39] by the de novo transcriptomic assembly program Trinity [40] using previously established protocols [41]. All settings for Inchworm, Chrysalis, and Butterfly steps were implemented according to the recommended protocol for fungal genomes, with the exception of the absence of the “–jaccard_clip” flag due to the low gene density of anaerobic fungal genomes. The assembly process was conducted on the Oklahoma State University High Performance Computing Cluster using a dual Intel Xeon E5-2620 “Sandy Bridge” hex core 2.0 GHz CPU node with 256 GB of RAM (https://hpcc.okstate.edu/content/cowboy-overview). Quantitative levels for all assembled transcripts were generated by mapping all generated sequencing reads to the assembled transcripts using the short read alignment mapping program Bowtie2 [42]. The quantitative program RSEM [43] was used to calculate all quantitative values in Fragments Per Kilobase of transcript per Million mapped reads (FPKM). To assess variability between biological replicates, the coefficient of determination *R*^2^ was calculated between biological replicate pairs using RSEM-generated FPKM values. All FPKM values were normalized to the library size using the R package DESeq [44]. The obtained p-values were used to assess the significance of transcripts’ up- and downregulation as shown in Tables 2, 3, and 4. All normalized FPKM values shown are averages of two biological replicates. Total normalized FPKM values of different GH families when Orpinomyces C1A was grown on different substrates were used in principal component analysis (PCA) using the R statistical package Labdsv [45], and the results were visualized in a biplot (Table 5).

**Table 5:** Transcriptional patterns of C1A cellulosomal genes on various substrates

Transcripts	Number of transcripts when grown on			
	Alfalfa	Energy cane	Corn stover	Sorghum
*Cellulosomal transcripts*				
Significantly upregulated^a^	52	53	56	47
Upregulated^b^	24	23	31	28
Downregulated^c^	32	29	34	22
Significantly downregulated^d^	47	49	54	41
No change^e^	141	143	121	158

^a^Significantly upregulated refers to the number of transcripts with a differential expression *p* value <0.01 as calculated by the nbinomTest function in the R package DESeq [44]

^b^Upregulated refers to the number of transcripts with a differential expression *p* value between 0.01 and 0.1 as calculated by the nbinomTest function in the R package DESeQ [44]

^c^Downregulated refers to the number of transcripts with a differential expression *p* value <0.01 as calculated by the nbinomTest function in the R package DESeq [44]

^d^Significantly downregulated refers to the number of transcripts with a differential expression *p* value between 0.01 and 0.1 as calculated by the nbinomTest function in the R package DESeq [44]

^e^No change refers to the number of transcripts with a differential expression *p* value >0.1 as calculated by the nbinomTest function in the R package DESeq [44]

### Transcript functional annotation and CAZyme identification

Transcript annotation of all genes was conducted using a combination of homology comparison to public databases, protein domain identification, and peptide secretion signal prediction. Predicted protein sequences from the assembled transcripts were generated using the Transdecoder software portion in the Trinity package [40]. Transcripts that were present in at least one condition with an FPKM ≥1 and contained a predicted peptide coding regions were used in subsequent analysis. Predicted peptides were compared to public databases to identify the phylogeny using NCBI Blast C++ [46], where an *e*-value of *e*^−5^ or less was used as a cutoff for Blast classification. Signal peptide prediction was conducted using signalP 4.0 [47] using the recommended settings and eukaryotic training set. Protein domain identification [48] was achieved using the hmmscan portion of the HMMER software package [49]. An *e*-value of *e*^−4^ was used as a cutoff for significance for domain assignment. All predicted peptide sequences were profiled against the PFAM 27.0 database [48] for general functional domain assignment. To specifically identify peptide sequences that are putative carbohydrate-active enzymes (CAZymes), all sequences were profiled against the Database for automated carbohydrate-active enzyme annotation (dbCAN) [50]. Sequences identified were further classified through manual curation and structural comparisons. Putative cellulosomal localization of transcripts was identified by the presence of the CBM_10 (Dockerin) domain that has previously been established as the enzyme attachment component to cellulosome in anaerobic fungi [51].

Differential transcriptional patterns between different conditions were analyzed by comparing Log_2_ [FPKM_biomass_/FPKM_glucose_] values. For inter-condition comparisons, a threshold of log_2_ ratio >1 and log_2_ ratio <−1 (corresponding to twofold over- or under-expression, respectively) was used to designate a specific transcript as significantly over- or under-expressed, respectively. Significance of transcripts’ up- and downregulation computed by this method was in general agreement with significantly different values (p-values) determined as described in supplementary document (Additional file 1: Table S6). To study the effect of plant biomass on the cellulosome composition, we utilized likelihood ratio Chi-squared test to examine the significant difference between the relative abundances of various protein categories in glucose-grown versus plant biomass-grown cultures.

### Sequence availability and accession numbers

Raw sequencing reads from each condition and the assembled transcript sequences will be available at GenBank under the accession number SRX1030108 and at MGRAST under the accession number 4667732.3. Raw and normalized transcriptional levels of all transcripts are available as a (Additional file 2).

## Additional files

10.1186/s13068-015-0390-0 FPKM values for all *Orpinomyces* sp. strain C1A transcripts grown on glucose, alfalfa, every cane, corn stover, and sorghum. For each substrate, two normalized FPKM values from two separate growth experiments as well as the average FPKM values are shown.
10.1186/s13068-015-0390-0 FPKM levels of all transcripts on the different substrates tested. Two FPKM values are shown for each transcript corresponding to two separate growth experiments. Average FPKM values used for all analyses are also shown.

## Abbreviations

C1A: *Orpinomyces* sp. strain C1A
FDD: fungal dockerin domain
GH: glycoside hydrolases
CE: carbohydrate esterases
PL: polysaccharide lyases
CAZymes: carbohydrate-active enzymes
FPKM: fragments per kilobase of transcript per million mapped reads
