# Supplementary material for: Transcriptomic analysis of lignocellulosic biomass degradation by the anaerobic fungal isolate Orpinomyces sp. strain C1A
Source: Biotechnol Biofuels. 2015 Dec 8;8:208. doi: 10.1186/s13068-015-0390-0 (PMC4672494; doi:10.1186/s13068-015-0390-0)
Supplement: Supplementary file 2 — 10.1186/s13068-015-0390-0 FPKM levels of all transcripts on the different substrates tested. Two FPKM values are shown for each transcript corresponding to two separate growth experiments. Average FPKM values used for all analyses are also shown. [file 13068_2015_390_MOESM2_ESM.docx]

Additional file 2

**Transcriptional analysis of lignocellulosic biomass degradation by the anaerobic fungal isolate *Orpinomyces* sp. strain C1A**

**Authors:** MB Couger, Noha H. Youssef, Christopher G. Struchtemeyer, Audra S. Liggenstoffer, and Mostafa Elshahed

**Figures legends.**

**Figure S1.** Venn diagram depicting shared CAZyme transcripts when grown under various growth conditions.

**Figure S2.** Correlation between number of transcripts and overall transcriptional levels in glycoside hydrolase families when C1A was grown on glucose (A), alfalfa (B), energy cane (C), corn stover (D), and sorghum (E). Pearson correlation coefficients are shown above each panel.

**Figure S3.** Venn diagram depicting shared fungal dockerin domains (putatively cellulosomal) transcripts when grown under various growth conditions.

Figure S1

Figure S2


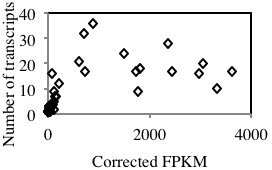

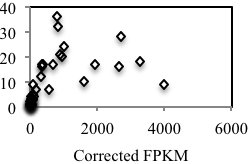

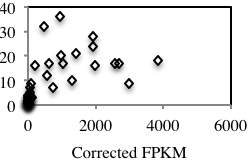

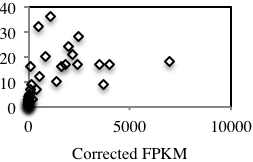

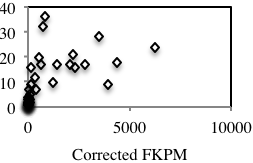


Pearson correlation coeff=0.6

Pearson correlation coeff=0.54

Pearson correlation coeff=0.61

Pearson correlation coeff=0.56

Pearson correlation coeff=0.61

Figure S3

**Table S1. Transcription levels of all GH and PL transcripts when grown on glucose (Glu) and lignocellulosic biomass (Alfalfa, Alf; Energy Cane, EC; Corn Stover, CS; Sorghum, Sor) substrates.**

| GH family | Transcript ID | Activity | Substrate | Phylogney | Bacterial/ HGT | Cellusomal | Average Absolute FKPM (corrected) ^a^ | | | | | Log_2_ transcription level (log_2_(ratio FKPM biomass: FKPM glucose)^b^ | | | |
| --- | --- | --- | --- | --- | --- | --- | --- | --- | --- | --- | --- | --- | --- | --- | --- |
|  |  |  |  |  |  |  | Glu | Alf | EC | CS | Sor | Alf | EC | CS | Sor |
| GH1 | m.18246 | beta-glucosidase | Cellulose | Eucalyptus grandis | NO | No | 1276.54 | 3164.05 | 1734.39 | 1718.37 | 997.83 | 0.87 | 0.00 | -0.01 | -0.80 |
|  | m.18245 | beta-glucosidase | Cellulose | Phytophthora parasitica INRA-310 | NO | No | 315.30 | 230.15 | 356.00 | 520.91 | 859.63 | -0.63 | 0.00 | 0.55 | 1.27 |
|  | m.18243 | beta-glucosidase | Cellulose | Phytophthora parasitica INRA-310 | NO | No | 232.37 | 95.84 | 309.98 | 604.74 | 1041.58 | -1.69 | 0.00 | 0.96 | 1.75 |
|  | m.18249 | beta-glucosidase | Cellulose | Eucalyptus grandis | NO | No | 205.29 | 476.04 | 384.48 | 502.72 | 589.98 | 0.31 | 0.00 | 0.39 | 0.62 |
|  | m.18239 | beta-glucosidase | Cellulose | Eucalyptus grandis | NO | No | 65.48 | 15.78 | 23.88 | 40.16 | 84.83 | -0.60 | 0.00 | 0.75 | 1.83 |
|  | m.18244 | beta-glucosidase | Cellulose | Phytophthora parasitica INRA-310 | NO | No | 27.93 | 8.72 | 15.51 | 19.07 | 60.06 | -0.83 | 0.00 | 0.30 | 1.95 |
|  | m.18241 | beta-glucosidase | Cellulose | Eucalyptus grandis | NO | No | 21.19 | 9.22 | 8.73 | 12.65 | 57.68 | 0.08 | 0.00 | 0.53 | 2.72 |
|  |  |  |  |  |  |  |  |  |  |  |  |  |  |  |  |
| GH2 | m.9329 | beta-galactosidase | Hemicellulose: xylan, xyloglucan, and galactoglucomannans, accessory | Ruminococcus flavefaciens | Yes | Yes | 7.13 | 8.31 | 1.24 | 0.98 | 3.89 | 2.74 | 0.00 | -0.34 | 1.64 |
|  |  |  |  |  |  |  |  |  |  |  |  |  |  |  |  |
| GH3 | m.19083 | beta-glucosidase | Cellulose | Mucor circinelloides f. circinelloides 1006PhL | NO | No | 321.40 | 230.70 | 395.27 | 1051.75 | 560.54 | -0.78 | 0.00 | 1.41 | 0.50 |
|  | m.3269 | beta-glucosidase | Cellulose | Rhizoctonia solani 123E | NO | Yes | 318.49 | 217.39 | 307.62 | 537.78 | 458.72 | -0.50 | 0.00 | 0.81 | 0.58 |
|  | m.21455 | Endoglucanase | Cellulose | Trametes versicolor FP-101664 SS1 | NO | No | 76.05 | 25.25 | 25.98 | 72.97 | 27.21 | -0.04 | 0.00 | 1.49 | 0.07 |
|  | m.19082 | beta-glucosidase | Cellulose | Mucor circinelloides f. circinelloides 1006PhL | NO | No | 39.10 | 49.30 | 86.44 | 1316.62 | 103.74 | -0.81 | 0.00 | 3.93 | 0.26 |
|  | m.24748 | beta-glucosidase | Cellulose | Roseburia intestinalis L1-82 | Yes | No | 32.57 | 59.43 | 55.03 | 75.64 | 28.54 | 0.11 | 0.00 | 0.46 | -0.95 |
|  | m.3264 | beta-glucosidase | Cellulose | Rhizoctonia solani 123E | NO | Yes | 28.62 | 27.97 | 44.62 | 19.40 | 11.37 | -0.67 | 0.00 | -1.20 | -1.97 |
|  | m.24594 | Endoglucanase | Cellulose | Rhizoctonia solani 123E | NO | No | 22.15 | 4.99 | 56.01 | 107.37 | 106.58 | -3.49 | 0.00 | 0.94 | 0.93 |
|  | m.20918 | beta-glucosidase | Cellulose | Roseburia intestinalis L1-82 | Yes | No | 9.09 | 8.08 | 0.70 | 4.28 | 1.24 | 3.53 | 0.00 | 2.61 | 0.83 |
|  | m.17079 | beta-glucosidase | Cellulose | Coprococcus comes ATCC 27758 | Yes | No | 5.10 | 3.83 | 0.15 | 0.14 | 0.27 | 4.72 | 0.00 | -0.09 | 0.89 |
|  | m.8381 | beta-glucosidase | Cellulose | Mucor circinelloides f. circinelloides 1006PhL | NO | No | 2.62 | 1.69 | 4.14 | 13.85 | 11.31 | -1.29 | 0.00 | 1.74 | 1.45 |
|  | m.5128 | beta-glucosidase | Cellulose | Butyrivibrio sp. XPD2006 | Yes | No | 2.11 | 2.11 | 2.58 | 2.10 | 1.41 | -0.29 | 0.00 | -0.30 | -0.87 |
|  | m.20060 | beta-glucosidase | Cellulose | Coprococcus comes ATCC 27758 | Yes | No | 1.67 | 2.08 | 0.14 | 3.99 | 0.52 | 3.85 | 0.00 | 4.79 | 1.84 |
|  | m.5129 | beta-glucosidase | Cellulose | Roseburia intestinalis L1-82 | Yes | No | 0.97 | 0.61 | 1.55 | 2.45 | 1.14 | -1.36 | 0.00 | 0.66 | -0.45 |
|  | m.20920 | beta-glucosidase | Cellulose | Roseburia intestinalis L1-82 | Yes | No | 0.26 | 1.79 | 2.78 | 0.00 | 0.32 | -0.63 | 0.00 | -21.41 | -3.10 |
|  | m.20059 | beta-glucosidase | Cellulose | Coprococcus comes ATCC 27758 | Yes | No | 0.23 | 1.93 | 1.40 | 2.26 | 1.01 | 0.46 | 0.00 | 0.69 | -0.46 |
|  | m.8557 | beta-glucosidase | Cellulose | Coprococcus comes ATCC 27758 | Yes | No | 0.15 | 1.50 | 1.74 | 1.43 | 0.85 | -0.21 | 0.00 | -0.28 | -1.04 |
|  | m.20917 | beta-glucosidase | Cellulose | Roseburia intestinalis L1-82 | Yes | No | 0.13 | 22.90 | 15.69 | 4.68 | 5.18 | 0.55 | 0.00 | -1.75 | -1.60 |
|  |  |  |  |  |  |  |  |  |  |  |  |  |  |  |  |
| GH4 | m.12199 | alpha Glucosidase | Hemicellulose: xylan | Trichoplax adhaerens | NO | NO | 21.87 | 2.57 | 5.99 | 9.78 | 4.90 | -1.22 | 0.00 | 0.71 | -0.29 |
|  | m.2527 | mitochondrial Enzyme | Other: Anabolic | Neocallimastix frontalis | NO | NO | 5.97 | 3.74 | 5.48 | 6.67 | 6.07 | -0.55 | 0.00 | 0.28 | 0.15 |
|  | m.15079 | 3-hydroxybutyryl-CoA dehydrogenase | Other: Anabolic | Lysinibacillus sphaericus C3-41 | Yes | NO | 2.04 | 0.01 | 0.07 | 0.00 | 0.01 | -3.69 | 0.00 | -16.15 | -2.32 |
|  |  |  |  |  |  |  |  |  |  |  |  |  |  |  |  |
| GH5 | m.22928 | endoglucanase | Cellulose | Clostridiaceae bacterium AN-C16-KBRB | Yes | Yes | 329.99 | 154.95 | 69.94 | 103.71 | 215.47 | 1.15 | 0.00 | 0.57 | 1.62 |
|  | m.9762 | endoglucanase | Cellulose | Cystobacter fuscus | Yes | Yes | 256.63 | 97.09 | 130.86 | 113.40 | 50.71 | -0.43 | 0.00 | -0.21 | -1.37 |
|  | m.22950 | endoglucanase | Cellulose | Paenibacillus curdlanolyticus | Yes | Yes | 120.31 | 127.52 | 297.28 | 309.42 | 174.92 | -1.22 | 0.00 | 0.06 | -0.77 |
|  | m.22947 | endoglucanase | Cellulose | Paenibacillus curdlanolyticus | Yes | No | 91.32 | 66.89 | 88.84 | 136.14 | 84.38 | -0.41 | 0.00 | 0.62 | -0.07 |
|  | m.3144 | endoglucanse | Cellulose | Acetivibrio cellulolyticus | Yes | No | 47.71 | 60.67 | 75.80 | 96.82 | 47.69 | -0.32 | 0.00 | 0.35 | -0.67 |
|  | m.2165 | beta mannadiosadse | Hemicellulose: mannans | Mucor circinelloides f. circinelloides 1006PhL | NO | No | 37.72 | 5.09 | 0.07 | 0.12 | 0.05 | 6.26 | 0.00 | 0.90 | -0.47 |
|  | m.22205 | endoglucanase | Cellulose | Ralstonia syzygii R24 | Yes | Yes | 35.10 | 7.02 | 9.81 | 4.47 | 13.57 | -0.48 | 0.00 | -1.13 | 0.47 |
|  | m.22932 | lichenase | Cellulose | Clostridium sp. KNHs205 | Yes | No | 25.40 | 39.99 | 18.95 | 19.63 | 10.20 | 1.08 | 0.00 | 0.05 | -0.89 |
|  | m.22929 | endoglucanase | Cellulose | Ruminococcus sp. CAG:379 | Yes | No | 23.08 | 44.23 | 22.68 | 21.55 | 10.85 | 0.96 | 0.00 | -0.07 | -1.06 |
|  | m.22959 | Triple Activity (endoglucanase, cellobiohydrolase and xylanase) | Cellulose | Ruminococcus flavefaciens | Yes | Yes | 20.00 | 4.95 | 32.73 | 29.38 | 0.00 | -2.73 | 0.00 | -0.16 | -24.96 |
|  | m.12565 | BetaMannanse | Hemicellulose: mannans | Rhizomucor miehei | NO | No | 18.49 | 12.44 | 11.67 | 14.06 | 10.35 | 0.09 | 0.00 | 0.27 | -0.17 |
|  | m.22216 | Endoglucanase | Cellulose | Ralstonia syzygii R24 | Yes | Yes | 10.96 | 11.21 | 4.82 | 7.24 | 9.01 | 1.22 | 0.00 | 0.58 | 0.90 |
|  | m.22213 | Endoglucanase | Cellulose | Ralstonia syzygii R24 | Yes | No | 7.86 | 0.38 | 0.86 | 2.09 | 4.68 | -1.18 | 0.00 | 1.29 | 2.45 |
|  | m.22953 | Triple Activity (endoglucanase, cellobiohydrolase and xylanase) | Cellulose | Ruminococcus flavefaciens | Yes | Yes | 7.01 | 36.83 | 8.01 | 38.09 | 19.56 | 2.20 | 0.00 | 2.25 | 1.29 |
|  | m.12584 | beta-mannanase | Hemicellulose: mannans | Dothistroma septosporum NZE10 | NO | Yes | 6.08 | 9.38 | 4.61 | 6.98 | 4.76 | 1.03 | 0.00 | 0.60 | 0.05 |
|  | m.22935 | Triple Activity (endoglucanase, cellobiohydrolase and xylanase) | Cellulose | Ruminococcus flavefaciens | Yes | Yes | 5.35 | 0.51 | 8.59 | 11.59 | 0.00 | -4.09 | 0.00 | 0.43 | -23.03 |
|  | m.20241 | Endoglucanase | Cellulose | Ruminococcus champanellensis 18P13 | Yes | No | 4.90 | 42.27 | 66.44 | 4.98 | 35.28 | -0.65 | 0.00 | -3.74 | -0.91 |
|  | m.22939 | endo-1 4-beta-xylanase | Hemicellulose: mannans | Paenibacillus sp. ICGEB2008 | Yes | Yes | 4.84 | 16.22 | 13.64 | 18.37 | 15.35 | 0.25 | 0.00 | 0.43 | 0.17 |
|  | m.12594 | beta-mannanase | Hemicellulose: mannans | Dothistroma septosporum NZE10 | NO | Yes | 4.02 | 3.06 | 1.80 | 4.49 | 3.41 | 0.76 | 0.00 | 1.32 | 0.92 |
|  | m.28243 | Endoglucanase | Cellulose | Lachnospiraceae bacterium A4 | Yes | No | 3.19 | 1.21 | 2.17 | 3.10 | 1.45 | -0.84 | 0.00 | 0.51 | -0.58 |
|  | m.1853 | Endoglucanase | Cellulose | Eubacterium siraeum V10Sc8a | Yes | Yes | 1.91 | 4.55 | 3.59 | 3.13 | 2.14 | 0.34 | 0.00 | -0.20 | -0.75 |
|  | m.22963 | Endoglucanase | Cellulose | Firmicutes bacterium CAG:882 | Yes | No | 1.15 | 0.79 | 1.71 | 3.41 | 2.70 | -1.11 | 0.00 | 0.99 | 0.66 |
|  | m.15645 | Endoglucanase | Cellulose | Lachnospiraceae bacterium A4 | Yes | No | 1.00 | 0.73 | 0.92 | 0.44 | 0.96 | -0.34 | 0.00 | -1.05 | 0.06 |
|  | m.20243 | Endoglucanase | Cellulose | Clostridiaceae bacterium AN-C16-KBRB | Yes | No | 0.88 | 1.96 | 0.52 | 0.75 | 0.39 | 1.93 | 0.00 | 0.53 | -0.42 |
|  | m.22960 | Triple Activity (endoglucanase, cellobiohydrolase and xylanase) | Cellulose | Ruminococcus flavefaciens | Yes | Yes | 0.66 | 2.27 | 1.92 | 6.39 | 7.75 | 0.24 | 0.00 | 1.74 | 2.01 |
|  | m.20242 | EndoGlucanse | Cellulose | Clostridium papyrosolvens | Yes | No | 0.56 | 1.54 | 1.28 | 0.54 | 0.58 | 0.26 | 0.00 | -1.26 | -1.15 |
|  | m.4936 | Endoglucanase | Cellulose | Eubacterium sp. CAG:156 | Yes | No | 0.45 | 4.58 | 0.76 | 0.54 | 0.66 | 2.58 | 0.00 | -0.50 | -0.21 |
|  | m.13330 | Endoglucanase | Cellulose | Ruminococcus sp. CAG:379 | Yes | No | 0.40 | 2.78 | 3.07 | 0.04 | 0.00 | -0.14 | 0.00 | -6.44 | -21.55 |
|  | m.22936 | Triple Activity (endoglucanase, cellobiohydrolase and xylanase) | Cellulose | Ruminococcus flavefaciens | Yes | Yes | 0.37 | 5.18 | 3.52 | 13.15 | 10.23 | 0.56 | 0.00 | 1.90 | 1.54 |
|  | m.12573 | beta-mannanase | Hemicellulose: mannans | Dothistroma septosporum NZE10 | NO | Yes | 0.34 | 0.16 | 1.38 | 0.15 | 0.50 | -3.12 | 0.00 | -3.23 | -1.46 |
|  | m.22210 | Endoglucanase | Cellulose | Ralstonia syzygii R24 | Yes | No | 0.02 | 0.18 | 0.62 | 1.19 | 2.15 | -1.77 | 0.00 | 0.95 | 1.80 |
|  | m.12576 | beta-mannanase | Hemicellulose: mannans | Dothistroma septosporum NZE10 | NO | Yes | 0.00 | 0.52 | 0.91 | 0.16 | 0.17 | -0.82 | 0.00 | -2.49 | -2.38 |
|  | m.22941 | Endoglucanase | Cellulose | Paenibacillus curdlanolyticus | Yes | Yes | 0.00 | 0.00 | 0.00 | 0.00 | 38.71 | 0.00 | 0.00 | 0.00 | 25.21 |
|  | m.22948 | endoglucanase | Cellulose | Paenibacillus sp. ICGEB2008 | Yes | Yes | 0.00 | 0.00 | 0.00 | 0.00 | 5.85 | 0.00 | 0.00 | 0.00 | 22.48 |
|  | m.22957 | Triple Activity (endoglucanase, cellobiohydrolase and xylanase) | Cellulose | Ruminococcus flavefaciens | Yes | Yes | 0.00 | 5.60 | 8.91 | 13.25 | 9.72 | -0.67 | 0.00 | 0.57 | 0.13 |
|  | m.22961 | Triple Activity (endoglucanase, cellobiohydrolase and xylanase) | Cellulose | Ruminococcus flavefaciens | Yes | Yes | 0.00 | 2.13 | 4.45 | 4.96 | 4.39 | -1.07 | 0.00 | 0.16 | -0.02 |
|  |  |  |  |  |  |  |  |  |  |  |  |  |  |  |  |
| GH6 | m.24006 | Cellobiohydrolase | Cellulose | Sorangium cellulosum | NO | No | 1307.21 | 2296.19 | 2655.30 | 5375.00 | 2767.60 | -0.21 | 0.00 | 1.02 | 0.06 |
|  | m.20902 | Cellobiohydrolase | Cellulose | Lachnobacterium bovis | Yes | No | 273.36 | 419.47 | 378.05 | 319.45 | 353.36 | 0.15 | 0.00 | -0.24 | -0.10 |
|  | m.24197 | Cellobiohydrolase | Cellulose | Sorangium cellulosum | Yes | Yes | 147.72 | 116.21 | 289.79 | 369.43 | 380.96 | -1.32 | 0.00 | 0.35 | 0.39 |
|  | m.14690 | Cellobiohydrolase | Cellulose | uncultured bacterium | Yes | No | 123.32 | 60.74 | 24.90 | 23.88 | 151.89 | 1.29 | 0.00 | -0.06 | 2.61 |
|  | m.20891 | Cellobiohydrolase | Cellulose | Paenibacillus mucilaginosus K02 | Yes | Yes | 82.63 | 44.38 | 143.86 | 89.25 | 51.49 | -1.70 | 0.00 | -0.69 | -1.48 |
|  | m.5221 | Cellobiohydrolase | Cellulose | Sorangium cellulosum | NO | No | 59.64 | 57.08 | 36.59 | 19.29 | 43.99 | 0.64 | 0.00 | -0.92 | 0.27 |
|  | m.6308 | Cellobiohydrolase | Cellulose | Pestalotiopsis fici W106-1 | NO | No | 46.74 | 1.18 | 21.57 | 42.16 | 2.23 | -4.19 | 0.00 | 0.97 | -3.28 |
|  | m.14691 | Cellobiohydrolase | Cellulose | uncultured bacterium | Yes | No | 24.43 | 30.89 | 17.09 | 23.33 | 169.15 | 0.85 | 0.00 | 0.45 | 3.31 |
|  | m.4741 | Cellobiohydrolase | Cellulose | Streptomyces sp. NRRL WC-3641 | Yes | No | 23.71 | 4.15 | 4.70 | 1.35 | 7.81 | -0.18 | 0.00 | -1.80 | 0.73 |
|  | m.6307 | Cellobiohydrolase | Cellulose | Ralstonia solanacearum | Yes | No | 23.13 | 0.41 | 19.53 | 39.54 | 2.15 | -5.59 | 0.00 | 1.02 | -3.18 |
|  | m.20871 | Cellobiohydrolase | Cellulose | Streptomyces sp. NRRL WC-3641 | Yes | Yes | 22.40 | 75.87 | 28.91 | 39.16 | 34.31 | 1.39 | 0.00 | 0.44 | 0.25 |
|  | m.20867 | Cellobiohydrolase | Cellulose | Stachybotrys chlorohalonata IBT 40285 | NO | No | 11.70 | 18.33 | 8.38 | 9.17 | 106.47 | 1.13 | 0.00 | 0.13 | 3.67 |
|  | m.22723 | Cellobiohydrolase | Cellulose | Aureobasidium subglaciale EXF-2481 | NO | No | 7.26 | 5.79 | 21.65 | 43.83 | 26.96 | -1.90 | 0.00 | 1.02 | 0.32 |
|  | m.5222 | Cellobiohydrolase | Cellulose | Streptomyces sp. CNT302 | Yes | Yes | 6.70 | 17.71 | 21.46 | 8.57 | 21.08 | -0.28 | 0.00 | -1.32 | -0.03 |
|  | m.20877 | Cellobiohydrolase | Cellulose | Streptomyces sp. NRRL WC-3641 | Yes | Yes | 3.84 | 28.01 | 9.76 | 6.59 | 5.21 | 1.52 | 0.00 | -0.57 | -0.90 |
|  | m.15300 | Cellobiohydrolase | Cellulose | Streptomyces sp. NRRL S-37 | Yes | No | 0.85 | 7.07 | 0.12 | 0.30 | 3.17 | 5.85 | 0.00 | 1.29 | 4.69 |
|  | m.5647 | Cellobiohydrolase | Cellulose | Aureobasidium pullulans EXF-150 | NO | No | 0.26 | 0.40 | 2.89 | 1.10 | 0.75 | -2.87 | 0.00 | -1.39 | -1.95 |
|  | m.20910 | Cellobiohydrolase | Cellulose | Streptomyces sp. NRRL WC-3641 | Yes | Yes | 0.00 | 2.41 | 3.77 | 9.94 | 5.20 | -0.65 | 0.00 | 1.40 | 0.47 |
|  |  |  |  |  |  |  |  |  |  |  |  |  |  |  |  |
| GH8 | m.4480 | endo-1 4-beta-xylanase | Hemicellulose: xylan | Fibrobacter succinogenes subsp. succinogenes S85 | Yes | Yes | 124.33 | 30.48 | 30.70 | 11.76 | 105.13 | -0.01 | 0.00 | -1.38 | 1.78 |
|  | m.27973 | Endoglucanase | Cellulose | Hahella chejuensis KCTC 2396 | Yes | No | 16.01 | 0.48 | 0.49 | 0.68 | 0.10 | -0.02 | 0.00 | 0.48 | -2.24 |
|  |  |  |  |  |  |  |  |  |  |  |  |  |  |  |  |
| GH9 | m.17946 | endoglucanase | Cellulose | Chlamydomonas reinhardtii | NO | Yes | 164.54 | 197.86 | 185.26 | 187.06 | 135.59 | 0.09 | 0.00 | 0.01 | -0.45 |
|  | m.17949 | endoglucanase | Cellulose | Chlamydomonas reinhardtii | NO | Yes | 74.88 | 235.86 | 348.29 | 487.68 | 646.08 | -0.56 | 0.00 | 0.49 | 0.89 |
|  | m.21525 | endoglucanase | Cellulose | Streptomyces achromogenes | Yes | Yes | 63.64 | 44.85 | 87.41 | 181.92 | 99.47 | -0.96 | 0.00 | 1.06 | 0.19 |
|  | m.21523 | Endoglucanase | Cellulose | Lentzea albidocapillata | Yes | No | 58.99 | 26.50 | 83.24 | 156.25 | 118.17 | -1.65 | 0.00 | 0.91 | 0.51 |
|  | m.17965 | Endoglucanase | Cellulose | Paenibacillus massiliensis | Yes | No | 46.45 | 9.29 | 18.14 | 51.70 | 91.56 | -0.97 | 0.00 | 1.51 | 2.34 |
|  | m.21540 | Endoglucanase | Cellulose | Agarivorans albus | Yes | No | 43.05 | 39.26 | 18.33 | 6.27 | 23.47 | 1.10 | 0.00 | -1.55 | 0.36 |
|  | m.17964 | Endoglucanase | Cellulose | Agarivorans albus | Yes | No | 42.15 | 145.63 | 282.37 | 415.17 | 519.45 | -0.96 | 0.00 | 0.56 | 0.88 |
|  | m.21531 | endoglucanase | Cellulose | Streptomyces achromogenes | Yes | Yes | 35.36 | 14.22 | 14.91 | 22.45 | 40.32 | -0.07 | 0.00 | 0.59 | 1.44 |
|  | m.21546 | Endoglucanase | Cellulose | Streptomyces sp. ATexAB-D23 | Yes | No | 35.28 | 36.12 | 85.35 | 137.82 | 105.82 | -1.24 | 0.00 | 0.69 | 0.31 |
|  | m.21547 | endoglucanase | Cellulose | Streptomyces sp. NRRL S-384 | Yes | Yes | 33.06 | 38.85 | 47.95 | 40.00 | 56.22 | -0.30 | 0.00 | -0.26 | 0.23 |
|  | m.17959 | Endoglucanase | Other | Batrachochytrium dendrobatidis JAM81 | NO | NO | 32.38 | 7.36 | 12.12 | 3.28 | 8.59 | -0.72 | 0.00 | -1.89 | -0.50 |
|  | m.21535 | Endoglucanase | Cellulose | Paenibacillus sp. FSL H7-689 | Yes | No | 28.64 | 12.75 | 54.86 | 95.67 | 77.27 | -2.11 | 0.00 | 0.80 | 0.49 |
|  | m.21532 | Endoglucanase | Cellulose | Zootermopsis nevadensis | NO | No | 18.09 | 13.67 | 52.40 | 97.38 | 85.65 | -1.94 | 0.00 | 0.89 | 0.71 |
|  | m.21545 | Endoglucanase | Cellulose | Streptomyces achromogenes | Yes | No | 17.89 | 20.48 | 29.32 | 51.43 | 40.02 | -0.52 | 0.00 | 0.81 | 0.45 |
|  | m.19665 | Endoglucanase | Cellulose | Clostridium thermocellum | Yes | No | 17.71 | 12.50 | 8.83 | 4.76 | 3.98 | 0.50 | 0.00 | -0.89 | -1.15 |
|  | m.21534 | Endoglucanase | Cellulose | Streptomyces sp. NRRL S-384 | Yes | No | 11.14 | 9.15 | 18.79 | 38.88 | 30.72 | -1.04 | 0.00 | 1.05 | 0.71 |
|  | m.21541 | endoglucanase | Cellulose | Streptomyces sp. NRRL S-384 | Yes | Yes | 4.33 | 3.49 | 3.98 | 39.00 | 0.92 | -0.19 | 0.00 | 3.29 | -2.11 |
|  | m.19664 | Endoglucanase | Cellulose | Caldicellulosiruptor bescii DSM 6725 | Yes | No | 3.98 | 1.35 | 0.21 | 1.40 | 3.70 | 2.68 | 0.00 | 2.73 | 4.13 |
|  | m.17966 | Endoglucanase | Cellulose | Kitasatospora setae | Yes | No | 1.86 | 0.54 | 0.00 | 0.00 | 0.00 | 19.04 | 0.00 | 0.00 | 0.00 |
|  | m.6489 | Endoglucanase | Cellulose | Physcomitrella patens | NO | No | 1.03 | 0.44 | 0.37 | 1.97 | 2.30 | 0.26 | 0.00 | 2.42 | 2.64 |
|  | m.4754 | Endoglucanase | Cellulose | Agarivorans albus | Yes | No | 0.67 | 0.56 | 0.36 | 0.10 | 2.04 | 0.66 | 0.00 | -1.81 | 2.51 |
|  |  |  |  |  |  |  |  |  |  |  |  |  |  |  |  |
| GH10 | m.20866 | endo-1 4-beta-xylanase | Hemicellulose: xylan | Aspergillus nidulans | NO | Yes | 554.48 | 286.11 | 78.30 | 76.20 | 208.62 | 1.87 | 0.00 | -0.04 | 1.41 |
|  | m.20865 | endo-1,4-beta-xylanase | Hemicellulose: xylan | Streptomyces sp. AA0539 | Yes | No | 452.89 | 492.35 | 231.06 | 485.81 | 1137.05 | 1.09 | 0.00 | 1.07 | 2.30 |
|  | m.9610 | endo-1 4-beta-xylanase | Hemicellulose: xylan | Cellulosilyticum ruminicola | Yes | Yes | 376.26 | 426.49 | 543.85 | 723.71 | 680.87 | -0.35 | 0.00 | 0.41 | 0.32 |
|  | m.20864 | endo-1,4-beta-xylanase | Hemicellulose: xylan | Clostridium straminisolvens JCM 21531 | Yes | No | 359.41 | 180.22 | 85.48 | 94.90 | 247.92 | 1.08 | 0.00 | 0.15 | 1.54 |
|  | m.9611 | endo-1,4-beta-xylanase | Hemicellulose: xylan | Saccharomonospora saliphila | NO | No | 229.27 | 393.57 | 400.46 | 454.40 | 432.00 | -0.03 | 0.00 | 0.18 | 0.11 |
|  | m.20906 | endo-1 4-beta-xylanase | Hemicellulose: xylan | Butyrivibrio proteoclasticus B316 | Yes | Yes | 180.42 | 320.01 | 221.58 | 180.60 | 222.57 | 0.53 | 0.00 | -0.30 | 0.01 |
|  | m.5263 | endo-1 4-beta-xylanase | Hemicellulose: xylan | Bacillus coagulans | Yes | Yes | 163.94 | 50.19 | 26.62 | 46.07 | 64.84 | 0.91 | 0.00 | 0.79 | 1.28 |
|  | m.25369 | Uknown Maybe Cellulase Xylanase | Hemicellulose: xylan | Eubacterium cellulosolvens | Yes | Yes | 163.39 | 21.10 | 7.46 | 26.01 | 6.20 | 1.50 | 0.00 | 1.80 | -0.27 |
|  | m.4223 | endo-1,4-beta-xylanase | Hemicellulose: xylan | Butyrivibrio proteoclasticus B316 | Yes | No | 83.21 | 59.34 | 55.98 | 27.98 | 95.30 | 0.08 | 0.00 | -1.00 | 0.77 |
|  | m.10067 | endo-1,4-beta-xylanase | Hemicellulose: xylan | Streptomyces sp. 351MFTsu5.1 | Yes | No | 78.42 | 74.04 | 16.08 | 3.23 | 3.36 | 2.20 | 0.00 | -2.31 | -2.26 |
|  | m.10245 | endo-1,4-beta-xylanase | Hemicellulose: xylan | Halomonas boliviensis | Yes | No | 44.38 | 11.32 | 14.00 | 30.60 | 44.39 | -0.31 | 0.00 | 1.13 | 1.66 |
|  | m.4221 | endo-1 4-beta-xylanase | Hemicellulose: xylan | Butyrivibrio proteoclasticus B316 | Yes | Yes | 27.18 | 16.08 | 31.75 | 27.11 | 25.90 | -0.98 | 0.00 | -0.23 | -0.29 |
|  | m.11945 | endo-1,4-beta-xylanase | Hemicellulose: xylan | Saccharomonospora paurometabolica | NO | No | 19.59 | 17.90 | 14.08 | 8.20 | 7.72 | 0.35 | 0.00 | -0.78 | -0.87 |
|  | m.26315 | EndoXylanase | Hemicellulose: xylan | Eubacterium cellulosolvens | Yes | No | 19.47 | 14.82 | 12.64 | 7.53 | 4.63 | 0.23 | 0.00 | -0.75 | -1.45 |
|  | m.5759 | endo-1,4-beta-xylanase | Hemicellulose: xylan | Thermopolyspora flexuosa | Yes | No | 16.93 | 6.35 | 1.58 | 0.45 | 7.33 | 2.01 | 0.00 | -1.82 | 2.22 |
|  | m.17819 | endo-1,4-beta-xylanase | Hemicellulose: xylan | Butyrivibrio proteoclasticus B316 | Yes | No | 16.72 | 3.34 | 5.77 | 3.16 | 2.30 | -0.79 | 0.00 | -0.87 | -1.33 |
|  | m.13022 | endo-1,4-beta-xylanase | Hemicellulose: xylan | Butyrivibrio proteoclasticus B316 | Yes | No | 15.61 | 71.66 | 9.57 | 32.95 | 28.53 | 2.90 | 0.00 | 1.78 | 1.58 |
|  | m.10244 | endo-1,4-beta-xylanase | Hemicellulose: xylan | Streptomyces sp. NRRL F-2305 | Yes | No | 15.49 | 11.22 | 29.86 | 18.52 | 19.95 | -1.41 | 0.00 | -0.69 | -0.58 |
|  | m.5379 | endo-1 4-beta-xylanase | Hemicellulose: xylan | Meiothermus cerbereus | Yes | Yes | 11.35 | 4.91 | 14.90 | 24.17 | 19.28 | -1.60 | 0.00 | 0.70 | 0.37 |
|  | m.5227 | endo-1,4-beta-xylanase | Hemicellulose: xylan | Meiothermus ruber DSM 1279 | Yes | No | 7.46 | 81.28 | 5.32 | 0.46 | 0.22 | 3.93 | 0.00 | -3.52 | -4.63 |
|  | m.15925 | endo-1,4-beta-xylanase | Hemicellulose: xylan | Meiothermus cerbereus | Yes | No | 4.25 | 18.19 | 10.19 | 4.06 | 2.58 | 0.84 | 0.00 | -1.33 | -1.98 |
|  | m.13024 | endo-1,4-beta-xylanase | Hemicellulose: xylan | Butyrivibrio proteoclasticus B316 | Yes | No | 2.87 | 13.64 | 2.75 | 20.35 | 15.96 | 2.31 | 0.00 | 2.89 | 2.54 |
|  | m.17820 | Endo Xylanse | Hemicellulose: xylan | Fomitiporia mediterranea MF3/22 | NO | No | 2.56 | 1.33 | 7.32 | 6.91 | 4.39 | -2.46 | 0.00 | -0.08 | -0.74 |
|  | m.5228 | endo-1,4-beta-xylanase | Hemicellulose: xylan | Micromonospora chokoriensis | Yes | No | 1.52 | 15.48 | 1.70 | 0.25 | 0.19 | 3.19 | 0.00 | -2.75 | -3.13 |
|  | m.20872 | endo-1 4-beta-xylanase | Hemicellulose: xylan | Butyrivibrio proteoclasticus B316 | Yes | Yes | 0.83 | 0.36 | 0.10 | 0.49 | 1.42 | 1.82 | 0.00 | 2.25 | 3.80 |
|  | m.12909 | endo-1 4-beta-xylanase | Hemicellulose: xylan | Clostridium sp. CAG:62 | Yes | Yes | 0.78 | 6.19 | 3.81 | 0.54 | 0.36 | 0.70 | 0.00 | -2.82 | -3.42 |
|  | m.8463 | endo-1,4-beta-xylanase | Hemicellulose: xylan | Meiothermus rufus | Yes | No | 0.53 | 19.37 | 2.99 | 0.88 | 0.75 | 2.69 | 0.00 | -1.76 | -2.00 |
|  | m.13105 | endo-1,4-beta-xylanase | Hemicellulose: xylan | Meiothermus ruber | Yes | No | 0.25 | 2.62 | 2.73 | 0.79 | 0.99 | -0.06 | 0.00 | -1.79 | -1.46 |
|  |  |  |  |  |  |  |  |  |  |  |  |  |  |  |  |
| GH11 | m.21119 | endo-1,4-beta-xylanase | Hemicellulose: xylan | Fibrobacter succinogenes | Yes | No | 550.68 | 211.55 | 778.46 | 474.92 | 2562.79 | -1.88 | 0.00 | -0.71 | 1.72 |
|  | m.21135 | endo-1,4-beta-xylanase | Hemicellulose: xylan | Fibrobacter succinogenes | Yes | No | 261.55 | 247.86 | 231.83 | 202.96 | 357.54 | 0.10 | 0.00 | -0.19 | 0.63 |
|  | m.21137 | endo-1,4-beta-xylanase | Hemicellulose: xylan | Fibrobacter succinogenes | Yes | No | 188.57 | 85.83 | 120.06 | 150.71 | 204.24 | -0.48 | 0.00 | 0.33 | 0.77 |
|  | m.21128 | endo-1 4-beta-xylanase | Hemicellulose: xylan | Fibrobacter succinogenes | Yes | Yes | 150.94 | 89.57 | 104.06 | 135.72 | 168.93 | -0.22 | 0.00 | 0.38 | 0.70 |
|  | m.21140 | endo-1,4-beta-xylanase | Hemicellulose: xylan | Fibrobacter succinogenes | Yes | No | 126.77 | 36.13 | 187.09 | 322.59 | 414.97 | -2.37 | 0.00 | 0.79 | 1.15 |
|  | m.21151 | endo-1,4-beta-xylanase | Hemicellulose: xylan | Fibrobacter succinogenes | Yes | No | 99.91 | 67.41 | 63.16 | 90.97 | 130.37 | 0.09 | 0.00 | 0.53 | 1.05 |
|  | m.21149 | endo-1,4-beta-xylanase | Hemicellulose: xylan | Fibrobacter succinogenes | Yes | No | 81.68 | 64.20 | 230.24 | 287.27 | 1672.44 | -1.84 | 0.00 | 0.32 | 2.86 |
|  | m.21126 | endo-1,4-beta-xylanase | Hemicellulose: xylan | Fibrobacter succinogenes | Yes | No | 56.06 | 20.27 | 9.98 | 51.96 | 63.99 | 1.02 | 0.00 | 2.38 | 2.68 |
|  | m.21124 | endo-1,4-beta-xylanase | Hemicellulose: xylan | Fibrobacter succinogenes | Yes | No | 45.18 | 12.39 | 30.87 | 47.71 | 107.15 | -1.32 | 0.00 | 0.63 | 1.80 |
|  | m.20630 | endo-1 4-beta-xylanase | Hemicellulose: xylan | Fibrobacter succinogenes | Yes | Yes | 39.01 | 0.94 | 2.30 | 2.05 | 3.12 | -1.29 | 0.00 | -0.17 | 0.44 |
|  | m.25297 | endo-1,4-beta-xylanase | Hemicellulose: xylan | Fibrobacter succinogenes | Yes | No | 32.90 | 102.22 | 11.20 | 9.70 | 19.23 | 3.19 | 0.00 | -0.21 | 0.78 |
|  | m.20631 | endo-1,4-beta-xylanase | Hemicellulose: xylan | Fibrobacter succinogenes | Yes | No | 24.50 | 0.54 | 0.78 | 2.27 | 2.54 | -0.53 | 0.00 | 1.54 | 1.70 |
|  | m.21144 | endo-1,4-beta-xylanase | Hemicellulose: xylan | Fibrobacter succinogenes | Yes | No | 14.87 | 9.24 | 15.86 | 13.13 | 15.94 | -0.78 | 0.00 | -0.27 | 0.01 |
|  | m.20626 | endo-1 4-beta-xylanase | Hemicellulose: xylan | Fibrobacter succinogenes | Yes | Yes | 14.87 | 0.75 | 1.41 | 2.05 | 2.91 | -0.91 | 0.00 | 0.54 | 1.04 |
|  | m.17862 | endo-1,4-beta-xylanase | Hemicellulose: xylan | Fibrobacter succinogenes | Yes | No | 14.43 | 10.03 | 6.23 | 2.89 | 26.03 | 0.69 | 0.00 | -1.11 | 2.06 |
|  | m.21148 | endo-1,4-beta-xylanase | Hemicellulose: xylan | Fibrobacter succinogenes | Yes | No | 6.69 | 4.53 | 7.80 | 7.06 | 16.59 | -0.78 | 0.00 | -0.14 | 1.09 |
|  | m.21122 | endo-1 4-beta-xylanase | Hemicellulose: xylan | Fibrobacter succinogenes | Yes | Yes | 6.64 | 3.56 | 0.90 | 1.47 | 4.97 | 1.98 | 0.00 | 0.71 | 2.46 |
|  | m.9977 | endo-1,4-beta-xylanase | Hemicellulose: xylan | uncultured microorganism | Yes | No | 4.17 | 10.70 | 8.72 | 13.80 | 82.16 | 0.29 | 0.00 | 0.66 | 3.24 |
|  | m.21130 | endo-1 4-beta-xylanase | Hemicellulose: xylan | Fibrobacter succinogenes | Yes | Yes | 2.92 | 2.48 | 1.85 | 5.02 | 4.20 | 0.42 | 0.00 | 1.44 | 1.18 |
|  | m.9099 | endo-1,4-beta-xylanase | Hemicellulose: xylan | Fibrobacter succinogenes | Yes | No | 1.05 | 0.81 | 4.54 | 3.66 | 4.56 | -2.48 | 0.00 | -0.31 | 0.01 |
|  | m.21134 | endo-1,4-beta-xylanase | Hemicellulose: xylan | Fibrobacter succinogenes | Yes | No | 0.61 | 0.47 | 0.24 | 1.31 | 0.36 | 0.95 | 0.00 | 2.44 | 0.55 |
|  | m.27491 | endo-1,4-beta-xylanase | Hemicellulose: xylan | Fibrobacter succinogenes | Yes | No | 0.25 | 3.99 | 4.41 | 2.47 | 2.65 | -0.15 | 0.00 | -0.84 | -0.74 |
|  | m.4414 | Unknown | Hemicellulose: xylan | Unknown | Uknown | No | 0.17 | 0.69 | 1.39 | 0.92 | 0.85 | -1.01 | 0.00 | -0.60 | -0.71 |
|  | m.21143 | endo-1,4-beta-xylanase | Hemicellulose: xylan | Fibrobacter succinogenes | Yes | No | 0.00 | 0.00 | 0.78 | 0.00 | 1.15 | -19.57 | 0.00 | -19.57 | 0.56 |
|  | m.22844 | endo-β-N-acetylglucosaminidase | Others (sulfate oligos) | None | None | NO | 43.24 | 2.18 | 7.60 | 0.53 | 0.78 | -1.80 | 0.00 | -3.85 | -3.29 |
|  |  |  |  |  |  |  |  |  |  |  |  |  |  |  |  |
| GH13 | m.24128 | alpha amylase | Starch | Streptococcus gallolyticus subsp. gallolyticus ATCC 43143 | Yes | NO | 1792.79 | 208.84 | 2319.81 | 1691.49 | 2326.23 | -3.47 | 0.00 | -0.46 | 0.00 |
|  | m.23494 | alpha amylase | Starch | Clostridium sp. CAG:411 | Yes | NO | 841.53 | 83.59 | 133.60 | 283.73 | 156.74 | -0.68 | 0.00 | 1.09 | 0.23 |
|  | m.23479 | alpha amylase | Starch | Clostridium sp. CAG:411 | Yes | NO | 158.79 | 4.57 | 40.45 | 25.44 | 44.00 | -3.15 | 0.00 | -0.67 | 0.12 |
|  | m.24240 | alpha amylase | Starch | Streptococcus gallolyticus | Yes | NO | 90.55 | 5.29 | 52.35 | 128.54 | 52.51 | -3.31 | 0.00 | 1.30 | 0.00 |
|  | m.14138 | alpha amylase | Starch | Streptococcus gallolyticus | Yes | NO | 19.77 | 2.72 | 7.85 | 12.75 | 13.49 | -1.53 | 0.00 | 0.70 | 0.78 |
|  | m.24668 | alpha amylase | Starch | Fomitiporia mediterranea MF3/22 | NO | NO | 15.28 | 1.33 | 15.39 | 17.57 | 12.63 | -3.54 | 0.00 | 0.19 | -0.28 |
|  | m.6193 | Chitanse | Starch | Bacillus Sp. Kr-8104 | Yes | NO | 3.59 | 0.15 | 2.40 | 16.89 | 8.42 | -3.98 | 0.00 | 2.82 | 1.81 |
|  | m.13415 | 1,4-alpha-glucan-branching enzyme | Starch | Agaricus bisporus var. bisporus H97 | NO | NO | 2.74 | 0.46 | 4.36 | 7.97 | 6.34 | -3.24 | 0.00 | 0.87 | 0.54 |
|  | m.14140 | alpha-amylase | Starch | Streptococcus dysgalactiae subsp. equisimilis ATCC 12394 | Yes | NO | 2.70 | 0.48 | 2.16 | 4.18 | 3.07 | -2.19 | 0.00 | 0.95 | 0.50 |
|  | m.14137 | alpha amylase | Starch | Streptococcus dysgalactiae subsp. equisimilis AC-2713 | Yes | NO | 2.53 | 0.24 | 0.79 | 8.77 | 8.79 | -1.70 | 0.00 | 3.48 | 3.48 |
|  | m.23496 | alpha amylase | Starch | Clostridium sp. CAG:411 | Yes | NO | 2.10 | 0.00 | 0.58 | 19.38 | 4.67 | -19.15 | 0.00 | 5.06 | 3.01 |
|  | m.9658 | alpha-amylase | Starch | Streptococcus dysgalactiae subsp. equisimilis ATCC 12394 | Yes | NO | 2.02 | 6.09 | 2.22 | 1.43 | 0.94 | 1.46 | 0.00 | -0.64 | -1.24 |
|  | m.9659 | alpha-amylase | Starch | Streptococcus dysgalactiae subsp. equisimilis ATCC 12394 | Yes | NO | 1.14 | 3.46 | 0.88 | 0.92 | 1.13 | 1.97 | 0.00 | 0.06 | 0.36 |
|  | m.13416 | 1,4-alpha-glucan-branching enzyme | Starch | Pyrenophora teres f. teres 0-1 | NO | NO | 1.08 | 0.83 | 2.49 | 9.61 | 6.48 | -1.59 | 0.00 | 1.95 | 1.38 |
|  | m.4078 | pullulanase | Starch | Haloplasma contractile SSD-17B | Yes | NO | 0.66 | 1.15 | 3.09 | 3.56 | 2.01 | -1.43 | 0.00 | 0.20 | -0.62 |
|  | m.8579 | alpha amylase | Starch | Paenibacillus sp. HW567 | Yes | NO | 0.00 | 8.18 | 0.74 | 0.15 | 0.05 | 3.47 | 0.00 | -2.32 | -3.90 |
|  | m.8580 | alpha amylase | Starch | Clostridium sp. CAG:411 | Yes | NO | 0.00 | 4.90 | 0.29 | 0.05 | 0.00 | 4.10 | 0.00 | -2.65 | -18.13 |
|  |  |  |  |  |  |  |  |  |  |  |  |  |  |  |  |
| GH16 | m.21795 | lichenase | Hemicellulose: Mixed-linkage (1→3),(1→4)-β-d-glucan | Lachnospiraceae bacterium AC2029 | Yes | NO | 1578.22 | 375.73 | 793.55 | 842.24 | 572.04 | -1.08 | 0.00 | 0.09 | -0.47 |
|  | m.21796 | lichenase | Hemicellulose: Mixed-linkage (1→3),(1→4)-β-d-glucan | Lachnospiraceae bacterium AC2029 | Yes | NO | 1513.99 | 670.16 | 321.46 | 334.62 | 464.51 | 1.06 | 0.00 | 0.06 | 0.53 |
|  | m.22355 | glucan endo-1,3-beta-D-glucosidase | Hemicellulose: Mixed-linkage (1→3),(1→4)-β-d-glucan | Maricaulis maris MCS10 | Yes | NO | 754.19 | 293.85 | 35.86 | 27.02 | 15.54 | 3.03 | 0.00 | -0.41 | -1.21 |
|  | m.5901 | xyloglucanase | Hemicellulose: xyloglucan | Chitinophaga pinensis DSM 2588 | Yes | No | 35.09 | 46.49 | 12.15 | 9.72 | 17.40 | 1.94 | 0.00 | -0.32 | 0.52 |
|  | m.20984 | glucan endo-1,3-beta-D-glucosidase | Hemicellulose: Mixed-linkage (1→3),(1→4)-β-d-glucan | Maricaulis maris MCS10 | Yes | NO | 13.68 | 18.25 | 12.77 | 22.58 | 12.62 | 0.52 | 0.00 | 0.82 | -0.02 |
|  | m.25351 | glucan endo-1,3-beta-D-glucosidase | Hemicellulose: Mixed-linkage (1→3),(1→4)-β-d-glucan | Chlorella variabilis | NO | NO | 10.01 | 90.22 | 27.62 | 14.71 | 11.13 | 1.71 | 0.00 | -0.91 | -1.31 |
|  | m.4601 | xyloglucanase | Hemicellulose: xyloglucan | Solanum lycopersicum | NO | No | 8.99 | 68.85 | 25.50 | 13.05 | 55.76 | 1.43 | 0.00 | -0.97 | 1.13 |
|  | m.20983 | glucan endo-1,3-beta-D-glucosidase | Hemicellulose: Mixed-linkage (1→3),(1→4)-β-d-glucan | Pedosphaera parvula | Yes | NO | 2.06 | 0.42 | 1.16 | 2.32 | 1.61 | -1.48 | 0.00 | 1.00 | 0.47 |
|  | m.22354 | glucan endo-1,3-beta-D-glucosidase | Hemicellulose: Mixed-linkage (1→3),(1→4)-β-d-glucan | Chlorella variabilis | NO | NO | 1.33 | 5.35 | 1.52 | 3.01 | 2.25 | 1.81 | 0.00 | 0.98 | 0.56 |
|  | m.27788 | glucan endo-1,3-beta-D-glucosidase | Hemicellulose: Mixed-linkage (1→3),(1→4)-β-d-glucan | Deinococcus gobiensis I-0 | Yes | NO | 1.11 | 7.55 | 1.65 | 0.88 | 1.04 | 2.19 | 0.00 | -0.91 | -0.67 |
|  |  |  |  |  |  |  |  |  |  |  |  |  |  |  |  |
| GH17 | m.28469 | Uknown | Uknown | Ectocarpus siliculosus | NO | NO | 0.00 | 0.00 | 0.65 | 1.09 | 0.66 | -19.30 | 0.00 | 0.76 | 0.04 |
|  |  |  |  |  |  |  |  |  |  |  |  |  |  |  |  |
| GH18 | m.5865 | Chitanse | Chitin | Paenibacillus elgii | Yes | NO | 102.78 | 146.42 | 255.38 | 238.80 | 128.24 | -0.80 | 0.00 | -0.10 | -0.99 |
|  | m.24320 | Chitinase | Chitin | Metarhizium acridum CQMa 102 | NO | NO | 67.08 | 75.69 | 136.47 | 158.19 | 122.46 | -0.85 | 0.00 | 0.21 | -0.16 |
|  | m.25521 | Chitanse | Chitin | Bacillus Circulans Wl-12 | Yes | NO | 35.79 | 3.34 | 10.63 | 15.06 | 5.42 | -1.67 | 0.00 | 0.50 | -0.97 |
|  | m.24841 | Chitanse | Chitin | Desmospora sp. 8437 | Yes | NO | 15.58 | 14.88 | 60.75 | 37.11 | 24.22 | -2.03 | 0.00 | -0.71 | -1.33 |
|  | m.592 | Chitanse | Chitin | Collimonas fungivorans Ter331 | Yes | NO | 10.50 | 29.26 | 20.59 | 15.56 | 8.96 | 0.51 | 0.00 | -0.40 | -1.20 |
|  | m.22734 | chitanse | Chitin | Trichomonas vaginalis G3 | NO | NO | 9.01 | 17.43 | 7.95 | 2.69 | 2.12 | 1.13 | 0.00 | -1.57 | -1.90 |
|  | m.9162 | Chitanse | Chitin | Bacillus circulans | Yes | NO | 3.26 | 12.07 | 13.44 | 9.82 | 6.92 | -0.15 | 0.00 | -0.45 | -0.96 |
|  | m.26008 | Chitanse | Chitin | Mucor circinelloides f. circinelloides 1006PhL | NO | NO | 2.39 | 8.09 | 11.92 | 3.70 | 3.83 | -0.56 | 0.00 | -1.69 | -1.64 |
|  | m.10222 | Chitanse | Chitin | Desmospora sp. 8437 | Yes | NO | 1.78 | 5.99 | 5.77 | 2.38 | 7.46 | 0.05 | 0.00 | -1.28 | 0.37 |
|  | m.9160 | Chitanse | Chitin | Neofusicoccum parvum UCRNP2 | NO | NO | 0.49 | 3.55 | 3.15 | 0.54 | 1.35 | 0.17 | 0.00 | -2.54 | -1.22 |
|  | m.28319 | Chitanse | Chitin | uncultured bacterium | Yes | NO | 0.25 | 0.14 | 1.52 | 0.66 | 0.61 | -3.45 | 0.00 | -1.20 | -1.31 |
|  | m.593 | Chitnase | Chitin | None | None | NO | 0.20 | 2.47 | 6.08 | 3.83 | 3.22 | -1.30 | 0.00 | -0.67 | -0.92 |
|  |  |  |  |  |  |  |  |  |  |  |  |  |  |  |  |
| GH20 | m.26951 | hexoamidase | Other: N-acetyl-β-D-hexosaminides | Mucor circinelloides f. circinelloides 1006PhL | NO | NO | 3.44 | 2.08 | 4.19 | 4.79 | 3.15 | -1.01 | 0.00 | 0.19 | -0.41 |
|  |  |  |  |  |  |  |  |  |  |  |  |  |  |  |  |
| GH25 | m.17967 | lysozyme | Peptidoglycan | Helcococcus kunzii | Yes | NO | 5.52 | 48.48 | 30.16 | 5.74 | 10.31 | 0.68 | 0.00 | -2.39 | -1.55 |
|  | m.17968 | lysozyme | Peptidoglycan | Helcococcus kunzii | Yes | NO | 2.81 | 15.01 | 10.21 | 2.64 | 6.27 | 0.56 | 0.00 | -1.95 | -0.70 |
|  | m.18592 | lysozyme | Peptidoglycan | Ruminococcus albus 7 | Yes | NO | 1.49 | 22.14 | 8.51 | 0.91 | 1.90 | 1.38 | 0.00 | -3.23 | -2.16 |
|  | m.8068 | lysozyme | Peptidoglycan | Salmonella enterica | Yes | NO | 1.06 | 2.56 | 0.57 | 0.00 | 0.23 | 2.17 | 0.00 | -19.12 | -1.29 |
|  |  |  |  |  |  |  |  |  |  |  |  |  |  |  |  |
| GH26 | m.20858 | endo-1,4-beta-mannosidase | Hemicellulose: Mannan | Paenibacillus sp. Br | Yes | Yes | 101.92 | 56.15 | 10.29 | 23.33 | 6.74 | 2.45 | 0.00 | 1.18 | -0.61 |
|  | m.19363 | beta-mannanase | Hemicellulose: Mannan | Fibrobacter succinogenes subsp. succinogenes S85 | Yes | Yes | 31.10 | 27.08 | 22.15 | 24.15 | 9.76 | 0.29 | 0.00 | 0.12 | -1.18 |
|  | m.20862 | endo-1,4-beta-mannosidase | Hemicellulose: Mannan | Paenibacillus sp. Br | Yes | Yes | 16.62 | 39.17 | 15.20 | 15.87 | 14.54 | 1.37 | 0.00 | 0.06 | -0.06 |
|  | m.20860 | endo-1,4-beta-mannosidase | Hemicellulose: Mannan | Ruminococcus flavefaciens | Yes | No | 10.90 | 36.11 | 10.89 | 9.52 | 5.27 | 1.73 | 0.00 | -0.19 | -1.05 |
|  | m.19362 | beta-mannanase | Hemicellulose: Mannan | Fibrobacter succinogenes subsp. succinogenes S85 | Yes | Yes | 3.74 | 5.86 | 4.99 | 6.41 | 5.24 | 0.23 | 0.00 | 0.36 | 0.07 |
|  | m.4105 | endo-1,4-beta-mannosidase | Hemicellulose: Mannan | Paenibacillus sp. Br | Yes | No | 1.92 | 0.96 | 2.48 | 2.21 | 1.22 | -1.37 | 0.00 | -0.16 | -1.03 |
|  | m.19364 | beta-mannanase-like protein | Hemicellulose: Mannan | Fibrobacter succinogenes subsp. succinogenes S85 | Yes | Yes | 0.00 | 0.80 | 3.97 | 5.71 | 1.12 | -2.31 | 0.00 | 0.52 | -1.83 |
|  |  |  |  |  |  |  |  |  |  |  |  |  |  |  |  |
| GH28 | m.17022 | maltose O-acetyltransferase | Pectin | Algoriphagus sp. PR1 | Yes | NO | 107.00 | 44.25 | 58.86 | 27.95 | 71.86 | -0.41 | 0.00 | -1.07 | 0.29 |
|  | m.17025 | Pectate lyase | Pectin | Streptomyces bingchenggensis BCW-1 | Yes | NO | 16.11 | 18.62 | 0.74 | 31.12 | 6.13 | 4.65 | 0.00 | 5.39 | 3.04 |
|  | m.28083 | polygalacturonase Pectinase | Pectin | Prevotella bergensis | Yes | NO | 9.18 | 1.48 | 1.72 | 0.54 | 2.43 | -0.22 | 0.00 | -1.66 | 0.50 |
|  | m.27777 | Pectate lyase | Pectin | Arthrobacter chlorophenolicus A6 | Yes | NO | 0.96 | 2.50 | 1.49 | 1.00 | 0.82 | 0.75 | 0.00 | -0.58 | -0.86 |
|  | m.19463 | acetaldehyde dehydrogenase | Pectin | Paenisporosarcina sp. TG-14 | Yes | NO | 0.08 | 0.30 | 0.00 | 0.00 | 0.05 | 18.22 | 0.00 | 0.00 | 15.48 |
|  |  |  |  |  |  |  |  |  |  |  |  |  |  |  |  |
| GH30 | m.8583 | alpha-L-arabinofuranosidase | Hemicellulose: xylan accessory | Roseburia sp. CAG:100 | Yes | No | 31.50 | 16.19 | 0.72 | 1.40 | 2.59 | 4.50 | 0.00 | 0.97 | 1.85 |
|  | m.5892 | alpha-L-arabinofuranosidase | Hemicellulose: xylan accessory | Roseburia sp. CAG:100 | Yes | No | 18.28 | 15.14 | 8.52 | 16.93 | 17.10 | 0.83 | 0.00 | 0.99 | 1.01 |
|  | m.8586 | alpha-L-arabinofuranosidase | Hemicellulose: xylan accessory | Roseburia sp. CAG:100 | Yes | No | 1.00 | 0.96 | 0.25 | 0.53 | 0.93 | 1.93 | 0.00 | 1.09 | 1.90 |
|  |  |  |  |  |  |  |  |  |  |  |  |  |  |  |  |
| GH31 | m.23493 | AlphaGlucosidase | Starch | Schizosaccharomyces pombe | NO | No | 637.05 | 152.48 | 204.05 | 543.30 | 140.80 | -0.42 | 0.00 | 1.41 | -0.54 |
|  | m.23495 | alpha-glucosidase | Starch | Amphimedon queenslandica | NO | NO | 281.35 | 50.01 | 28.49 | 45.60 | 67.00 | 0.81 | 0.00 | 0.68 | 1.23 |
|  | m.23498 | alpha-glucosidase | Starch | Amphimedon queenslandica | NO | NO | 267.39 | 15.14 | 92.57 | 246.81 | 104.25 | -2.61 | 0.00 | 1.41 | 0.17 |
|  | m.4801 | Alpha Glucosidase | Starch | Trichoplax adhaerens | NO | NO | 230.17 | 37.53 | 31.26 | 39.73 | 32.22 | 0.26 | 0.00 | 0.35 | 0.04 |
|  | m.23487 | alpha-glucosidase | Starch | Amphimedon queenslandica | NO | NO | 196.96 | 47.53 | 55.61 | 208.54 | 62.63 | -0.23 | 0.00 | 1.91 | 0.17 |
|  | m.23502 | Alpha Glucosidase | Starch | Trichoplax adhaerens | NO | NO | 161.96 | 9.10 | 49.60 | 185.02 | 54.37 | -2.45 | 0.00 | 1.90 | 0.13 |
|  | m.23484 | alpha-glucosidase | Starch | Amphimedon queenslandica | NO | NO | 105.02 | 22.57 | 21.02 | 41.93 | 33.87 | 0.10 | 0.00 | 1.00 | 0.69 |
|  | m.23491 | alpha-glucosidase | Starch | Amphimedon queenslandica | NO | NO | 78.78 | 3.09 | 14.93 | 148.44 | 27.99 | -2.27 | 0.00 | 3.31 | 0.91 |
|  | m.23500 | alpha-glucosidase | Starch | Amphimedon queenslandica | NO | NO | 78.06 | 11.87 | 38.15 | 126.61 | 20.54 | -1.68 | 0.00 | 1.73 | -0.89 |
|  | m.23503 | alpha-glucosidase | Starch | Amphimedon queenslandica | NO | NO | 55.03 | 6.44 | 10.41 | 45.17 | 33.37 | -0.69 | 0.00 | 2.12 | 1.68 |
|  | m.23489 | alpha-glucosidase | Starch | Amphimedon queenslandica | NO | NO | 20.58 | 0.41 | 3.30 | 0.45 | 0.23 | -3.02 | 0.00 | -2.87 | -3.82 |
|  | m.11813 | glucan 1,3-alpha-glucosidase | Starch | Batrachochytrium dendrobatidis JAM81 | NO | NO | 12.21 | 5.72 | 28.54 | 30.54 | 25.86 | -2.32 | 0.00 | 0.10 | -0.14 |
|  | m.26057 | Unkown | Starch | Debaryomyces hansenii CBS767 | NO | NO | 11.33 | 1.10 | 3.65 | 0.62 | 0.47 | -1.73 | 0.00 | -2.55 | -2.95 |
|  | m.287 | Alpha Glucosidase | Starch | Trichoplax adhaerens | NO | NO | 1.44 | 1.08 | 5.05 | 6.83 | 3.44 | -2.22 | 0.00 | 0.44 | -0.55 |
|  | m.8666 | Alpha-glucosidase | Starch | Pedosphaera parvula | Yes | NO | 0.57 | 1.46 | 3.13 | 2.77 | 1.59 | -1.10 | 0.00 | -0.18 | -0.98 |
|  | m.8665 | alpha-glucosidase | Starch | Amphimedon queenslandica | NO | NO | 0.30 | 0.65 | 1.89 | 1.47 | 1.05 | -1.55 | 0.00 | -0.36 | -0.85 |
|  | m.23480 | alpha-glucosidase | Starch | Amphimedon queenslandica | NO | NO | 0.08 | 0.06 | 0.00 | 0.03 | 3.54 | 15.76 | 0.00 | 14.72 | 21.75 |
|  |  |  |  |  |  |  |  |  |  |  |  |  |  |  |  |
| GH32 | m.19593 | Fructan Hydrolase | Other: Fructan | Atopobium parvulum DSM 20469 |  | NO | 30.92 | 8.47 | 26.22 | 13.47 | 16.32 | -1.63 | 0.00 | -0.96 | -0.68 |
|  | m.8100 | Fructan Hydrolase | Other: Fructan | Streptococcus equinus | Yes | NO | 0.37 | 1.72 | 0.00 | 0.00 | 0.00 | 20.71 | 0.00 | 0.00 | 0.00 |
|  |  |  |  |  |  |  |  |  |  |  |  |  |  |  |  |
| GH36 | m.25889 | raffinose synthase | Other: Fructan | Leptosphaeria maculans JN3 | NO | NO | 4.04 | 1.85 | 6.60 | 7.58 | 4.58 | -1.84 | 0.00 | 0.20 | -0.53 |
|  |  |  |  |  |  |  |  |  |  |  |  |  |  |  |  |
| GH37 | m.5183 | trehalase | Other: Trehalose | Batrachochytrium dendrobatidis JAM81 | NO | NO | 14.47 | 6.51 | 8.86 | 7.90 | 3.96 | -0.44 | 0.00 | -0.17 | -1.16 |
|  |  |  |  |  |  |  |  |  |  |  |  |  |  |  |  |
| GH38 | m.26988 | Alpha Mannosidase | Hemicellulose: mannan | Mucor circinelloides f. circinelloides 1006PhL | NO | No | 3.84 | 1.05 | 6.85 | 3.84 | 3.24 | -2.71 | 0.00 | -0.84 | -1.08 |
|  |  |  |  |  |  |  |  |  |  |  |  |  |  |  |  |
| GH39 | m.21910 | Beta-xylosidase | Hemicellulose: xylan | Clostridium saccharoperbutylacetonicum N1-4(HMT) | Yes | No | 108.04 | 50.90 | 74.65 | 109.63 | 91.25 | -0.55 | 0.00 | 0.55 | 0.29 |
|  | m.21913 | Beta-xylosidase | Hemicellulose: xylan | Clostridium saccharoperbutylacetonicum N1-4(HMT) | Yes | No | 14.81 | 9.86 | 1.28 | 12.67 | 11.19 | 2.95 | 0.00 | 3.31 | 3.13 |
|  | m.21914 | Beta-xylosidase | Hemicellulose: xylan | Clostridium saccharoperbutylacetonicum N1-4(HMT) | Yes | No | 5.84 | 7.21 | 4.16 | 11.64 | 11.37 | 0.79 | 0.00 | 1.48 | 1.45 |
|  | m.21917 | Beta-xylosidase | Hemicellulose: xylan | Clostridium saccharoperbutylacetonicum N1-4(HMT) | Yes | No | 5.45 | 3.59 | 2.05 | 2.90 | 2.21 | 0.81 | 0.00 | 0.50 | 0.11 |
|  | m.21918 | Beta-xylosidase | Hemicellulose: xylan | Clostridium saccharoperbutylacetonicum N1-4(HMT) | Yes | No | 2.80 | 1.35 | 1.53 | 2.45 | 2.74 | -0.18 | 0.00 | 0.68 | 0.84 |
|  | m.21916 | Beta-xylosidase | Hemicellulose: xylan | Clostridium saccharoperbutylacetonicum N1-4(HMT) | Yes | No | 2.61 | 8.77 | 0.17 | 0.00 | 0.00 | 5.73 | 0.00 | -17.33 | -17.33 |
|  | m.21909 | Beta-xylosidase | Hemicellulose: xylan | Clostridium saccharoperbutylacetonicum N1-4(HMT) | Yes | No | 1.91 | 0.36 | 2.64 | 3.67 | 3.49 | -2.88 | 0.00 | 0.48 | 0.40 |
|  | m.5857 | Beta-xylosidase | Hemicellulose: xylan | Sorangium cellulosum So ce56 | Yes | No | 1.22 | 2.15 | 1.56 | 0.46 | 0.50 | 0.47 | 0.00 | -1.76 | -1.62 |
|  | m.21912 | Beta-xylosidase | Hemicellulose: xylan | Clostridium saccharoperbutylacetonicum N1-4(HMT) | Yes | No | 0.00 | 3.54 | 1.11 | 0.00 | 0.00 | 1.68 | 0.00 | -20.08 | -20.08 |
|  |  |  |  |  |  |  |  |  |  |  |  |  |  |  |  |
| GH43 | m.3145 | alpha-N-arabinofuranosidase | Hemicellulose: xylan | Clostridium lentocellum DSM 5427 | Yes | Yes | 224.58 | 271.01 | 227.58 | 218.79 | 269.46 | 0.25 | 0.00 | -0.06 | 0.24 |
|  | m.5510 | Beta-xylosidase | Hemicellulose: xylan | Ruminococcus champanellensis 18P13 | Yes | No | 212.68 | 35.32 | 10.64 | 42.41 | 69.25 | 1.73 | 0.00 | 2.00 | 2.70 |
|  | m.21473 | xylosidase/arabinofuranosidase | Hemicellulose: xylan | Butyrivibrio sp. VCB2006 | Yes | Yes | 84.64 | 125.02 | 9.02 | 2.37 | 13.99 | 3.79 | 0.00 | -1.93 | 0.63 |
|  | m.18551 | alpha-N-arabinofuranosidase | Hemicellulose: xylan | Clostridium lentocellum DSM 5427 | Yes | No | 78.19 | 79.55 | 19.36 | 29.47 | 75.33 | 2.04 | 0.00 | 0.61 | 1.96 |
|  | m.17508 | beta-xylosidase | Hemicellulose: xylan | Ruminococcus sp. CAG:379 | Yes | No | 77.12 | 18.88 | 1.78 | 2.39 | 2.77 | 3.41 | 0.00 | 0.43 | 0.64 |
|  | m.516 | alpha-N-arabinofuranosidase | Hemicellulose: xylan | Ruminococcus flavefaciens | Yes | Yes | 39.17 | 13.09 | 3.12 | 2.09 | 15.03 | 2.07 | 0.00 | -0.58 | 2.27 |
|  | m.4908 | alpha-N-arabinofuranosidase | Hemicellulose: xylan | Rhodanobacter fulvus | Yes | No | 34.12 | 6.12 | 3.83 | 3.85 | 4.77 | 0.68 | 0.00 | 0.01 | 0.32 |
|  | m.21475 | xylosidase/arabinofuranosidase | Hemicellulose: xylan | Butyrivibrio proteoclasticus B316 | Yes | No | 20.29 | 22.23 | 30.53 | 28.75 | 30.43 | -0.46 | 0.00 | -0.09 | 0.00 |
|  | m.18557 | alpha-N-arabinofuranosidase | Hemicellulose: xylan | Clostridium lentocellum DSM 5427 | Yes | No | 17.83 | 39.46 | 21.79 | 12.26 | 33.71 | 0.86 | 0.00 | -0.83 | 0.63 |
|  | m.22260 | Beta-xylosidase | Hemicellulose: xylan | Spirochaeta thermophila DSM 6192 | Yes | No | 10.40 | 7.20 | 1.04 | 1.85 | 3.38 | 2.79 | 0.00 | 0.83 | 1.70 |
|  | m.12905 | Beta-xylosidase | Hemicellulose: xylan | Spirochaeta thermophila DSM 6192 | Yes | No | 9.33 | 41.12 | 14.13 | 11.14 | 8.97 | 1.54 | 0.00 | -0.34 | -0.66 |
|  | m.18553 | endo-1,4-beta-xylanase | Hemicellulose: xylan | Cellulosilyticum ruminicola | Yes | No | 9.33 | 17.12 | 19.06 | 10.43 | 46.55 | -0.16 | 0.00 | -0.87 | 1.29 |
|  | m.22256 | Beta-xylosidase | Hemicellulose: xylan | Spirochaeta thermophila DSM 6192 | Yes | No | 8.54 | 6.97 | 1.13 | 2.58 | 4.34 | 2.62 | 0.00 | 1.19 | 1.94 |
|  | m.18549 | alpha-N-arabinofuranosidase | Hemicellulose: xylan | Clostridium lentocellum DSM 5427 | Yes | Yes | 5.44 | 28.57 | 11.39 | 17.37 | 16.96 | 1.33 | 0.00 | 0.61 | 0.58 |
|  | m.18546 | alpha-N-arabinofuranosidase | Hemicellulose: xylan | Clostridium lentocellum DSM 5427 | Yes | No | 4.98 | 24.56 | 7.61 | 7.16 | 22.91 | 1.69 | 0.00 | -0.09 | 1.59 |
|  | m.11616 | β-xylosidase | Hemicellulose: xylan | Firmicutes bacterium CAG:882 | Yes | No | 4.94 | 11.76 | 6.09 | 0.48 | 2.70 | 0.95 | 0.00 | -3.65 | -1.17 |
|  | m.18550 | endo-1,4-beta-xylanase | Hemicellulose: xylan | Cellulosilyticum ruminicola | Yes | No | 3.96 | 5.74 | 5.82 | 6.70 | 16.90 | -0.02 | 0.00 | 0.20 | 1.54 |
|  | m.15103 | endo-1,4-beta-xylanase | Hemicellulose: xylan | Cellulosilyticum ruminicola | Yes | No | 3.70 | 4.51 | 0.17 | 0.37 | 0.18 | 4.77 | 0.00 | 1.15 | 0.13 |
|  | m.19934 | Cellbiohydrolase | Hemicellulose: xylan | Butyrivibrio fibrisolvens 16/4 | Yes | No | 3.50 | 9.02 | 11.97 | 10.60 | 6.77 | -0.41 | 0.00 | -0.17 | -0.82 |
|  | m.17511 | beta-xylosidase | Hemicellulose: xylan | Ruminococcus sp. CAG:379 | Yes | No | 2.78 | 4.50 | 2.81 | 3.50 | 3.91 | 0.68 | 0.00 | 0.32 | 0.48 |
|  | m.19922 | endo-1,4-beta-xylanase | Hemicellulose: xylan | Cellulosilyticum ruminicola | Yes | No | 2.09 | 5.04 | 5.98 | 6.57 | 3.68 | -0.25 | 0.00 | 0.14 | -0.70 |
|  | m.11615 | β-xylosidase | Hemicellulose: xylan | Firmicutes bacterium CAG:882 | Yes | No | 2.03 | 0.00 | 0.00 | 2.90 | 12.12 | 0.00 | 0.00 | 21.47 | 23.53 |
|  | m.5568 | EndoXylanase | Hemicellulose: xylan | Ruminococcus sp. CAG:403 | Yes | No | 1.92 | 0.68 | 1.58 | 1.87 | 1.67 | -1.22 | 0.00 | 0.24 | 0.08 |
|  | m.19926 | Endoglucanase | Hemicellulose: xylan | Lachnobacterium bovis | Yes | No | 1.79 | 4.69 | 4.65 | 14.49 | 9.02 | 0.01 | 0.00 | 1.64 | 0.95 |
|  | m.19935 | Endoglucanase | Hemicellulose: xylan | Lachnobacterium bovis | Yes | No | 1.76 | 7.75 | 11.44 | 6.74 | 4.86 | -0.56 | 0.00 | -0.76 | -1.24 |
|  | m.18548 | alpha-N-arabinofuranosidase | Hemicellulose: xylan | Ruminococcus albus | Yes | NO | 1.70 | 1.20 | 1.18 | 5.61 | 4.64 | 0.03 | 0.00 | 2.25 | 1.98 |
|  | m.21471 | xylosidase/arabinofuranosidase | Hemicellulose: xylan | Butyrivibrio proteoclasticus B316 | Yes | No | 0.80 | 0.88 | 1.33 | 0.00 | 8.16 | -0.60 | 0.00 | -20.34 | 2.62 |
|  | m.22254 | Beta-xylosidase | Hemicellulose: xylan | Spirochaeta thermophila DSM 6192 | Yes | No | 0.57 | 1.46 | 0.30 | 0.45 | 0.72 | 2.27 | 0.00 | 0.57 | 1.24 |
|  | m.18552 | alpha-N-arabinofuranosidase | Hemicellulose: xylan | Clostridium lentocellum DSM 5427 | Yes | No | 0.55 | 1.44 | 2.18 | 2.05 | 1.70 | -0.60 | 0.00 | -0.09 | -0.36 |
|  | m.22258 | Beta-xylosidase | Hemicellulose: xylan | Spirochaeta thermophila DSM 6192 | Yes | No | 0.47 | 2.19 | 2.32 | 1.55 | 1.26 | -0.08 | 0.00 | -0.59 | -0.88 |
|  | m.13009 | alpha-N-arabinofuranosidase | Hemicellulose: xylan | Ruminococcus flavefaciens | Yes | No | 0.06 | 1.58 | 1.02 | 1.17 | 0.70 | 0.62 | 0.00 | 0.19 | -0.54 |
|  | m.21472 | xylosidase/arabinofuranosidase | Hemicellulose: xylan | Butyrivibrio proteoclasticus B316 | Yes | No | 0.00 | 2.13 | 1.89 | 1.07 | 8.21 | 0.17 | 0.00 | -0.82 | 2.12 |
|  |  |  |  |  |  |  |  |  |  |  |  |  |  |  |  |
| GH45 | m.9718 | Endoglucanase | Cellulose | Setosphaeria turcica Et28A | NO | No | 761.65 | 514.04 | 262.59 | 127.97 | 190.13 | 0.97 | 0.00 | -1.04 | -0.47 |
|  | m.23474 | Endoglucanase | Cellulose | Syncephalastrum racemosum | NO | No | 748.02 | 185.68 | 83.52 | 55.38 | 313.20 | 1.15 | 0.00 | -0.59 | 1.91 |
|  | m.23473 | Endoglucanase | Cellulose | Syncephalastrum racemosum | NO | No | 656.75 | 301.06 | 487.21 | 431.45 | 711.11 | -0.69 | 0.00 | -0.18 | 0.55 |
|  | m.23469 | Endoglucanase | Cellulose | Rhizopus delemar RA 99-880 | NO | Yes | 283.07 | 113.25 | 82.83 | 71.01 | 142.76 | 0.45 | 0.00 | -0.22 | 0.79 |
|  | m.9724 | Endoglucanase | Cellulose | Humicola Insolens | NO | No | 244.75 | 230.94 | 156.12 | 73.28 | 134.77 | 0.56 | 0.00 | -1.09 | -0.21 |
|  | m.22176 | EndoGlucanse | Cellulose | Rhizopus oryzae | NO | NO | 167.58 | 260.93 | 140.46 | 89.53 | 86.47 | 0.89 | 0.00 | -0.65 | -0.70 |
|  | m.9712 | EndoGlucanse | Cellulose | Humicola Insolens | NO | Yes | 154.09 | 195.47 | 121.42 | 78.15 | 99.47 | 0.69 | 0.00 | -0.64 | -0.29 |
|  | m.23475 | Endoglucanase | Cellulose | Rhizopus delemar RA 99-880 | NO | Yes | 142.77 | 22.52 | 34.19 | 66.85 | 55.20 | -0.60 | 0.00 | 0.97 | 0.69 |
|  | m.22179 | Endoglucanase | Cellulose | Macrophomina phaseolina MS6 | NO | No | 96.03 | 111.94 | 93.90 | 161.02 | 135.63 | 0.25 | 0.00 | 0.78 | 0.53 |
|  | m.20884 | EndoGlucanase | Cellulose | Rhodosporidium toruloides NP11 | Yes | Yes | 85.37 | 51.16 | 84.15 | 142.88 | 64.12 | -0.72 | 0.00 | 0.76 | -0.39 |
|  | m.23467 | Endoglucanase | Cellulose | Pyrenophora tritici-repentis Pt-1C-BFP | NO | No | 72.47 | 50.86 | 59.25 | 88.24 | 46.33 | -0.22 | 0.00 | 0.57 | -0.35 |
|  | m.23471 | Endoglucanase | Cellulose | Syncephalastrum racemosum | NO | No | 66.55 | 15.79 | 38.54 | 49.48 | 60.09 | -1.29 | 0.00 | 0.36 | 0.64 |
|  | m.22178 | Endoglucanase | Cellulose | Rhizopus delemar RA 99-880 | NO | No | 58.42 | 473.62 | 208.90 | 75.51 | 117.12 | 1.18 | 0.00 | -1.47 | -0.83 |
|  | m.15365 | Endoglucanase | Cellulose | Rhizopus oryzae | NO | No | 14.03 | 43.15 | 1.15 | 2.63 | 21.76 | 5.23 | 0.00 | 1.19 | 4.24 |
|  | m.15366 | Endoglucanase | Cellulose | Bursaphelenchus xylophilus | NO | No | 5.29 | 54.77 | 28.94 | 9.72 | 4.85 | 0.92 | 0.00 | -1.57 | -2.58 |
|  | m.1948 | Endoglucanase | Cellulose | Rhizopus delemar RA 99-880 | NO | No | 1.15 | 4.90 | 1.52 | 4.68 | 2.42 | 1.69 | 0.00 | 1.62 | 0.67 |
|  |  |  |  |  |  |  |  |  |  |  |  |  |  |  |  |
| GH47 | m.25576 | α-mannosidase | Other: Anabolic | Saccoglossus kowalevskii | NO | NO | 14.65 | 4.26 | 27.31 | 14.84 | 12.35 | -2.68 | 0.00 | -0.88 | -1.14 |
|  | m.10198 | EndoPlasmic Reticulium Enzmye | Other: Anabolic | Mucor circinelloides f. circinelloides 1006PhL | NO | NO | 0.42 | 1.08 | 2.71 | 1.03 | 0.99 | -1.32 | 0.00 | -1.40 | -1.45 |
|  | m.1724 | EndoPlasmic Reticulium Enzmye | Other: Anabolic | Mucor circinelloides f. circinelloides 1006PhL | NO | NO | 0.41 | 0.00 | 1.09 | 2.12 | 0.88 | -20.05 | 0.00 | 0.96 | -0.31 |
|  |  |  |  |  |  |  |  |  |  |  |  |  |  |  |  |
| GH48 | m.19942 | Processive Cellulase | Cellulose | Clostridium cellobioparum | Yes | Yes | 2866.17 | 319.23 | 92.59 | 32.17 | 175.04 | 1.79 | 0.00 | -1.53 | 0.92 |
|  | m.19936 | Processive Cellulase | Cellulose | Streptomyces | Yes | No | 800.33 | 92.34 | 35.95 | 21.73 | 99.39 | 1.36 | 0.00 | -0.73 | 1.47 |
|  | m.24017 | Processive Cellulase | Cellulose | Cystobacter fuscus | Yes | Yes | 164.33 | 313.89 | 795.17 | 1336.17 | 573.63 | -1.34 | 0.00 | 0.75 | -0.47 |
|  | m.24007 | Processive Cellulase | Cellulose | Cystobacter fuscus | Yes | Yes | 150.90 | 29.41 | 192.54 | 753.09 | 387.79 | -2.71 | 0.00 | 1.97 | 1.01 |
|  | m.20869 | Processive Cellulase | Cellulose | Clostridium cellobioparum | Yes | Yes | 124.06 | 640.19 | 445.42 | 217.02 | 149.37 | 0.52 | 0.00 | -1.04 | -1.58 |
|  | m.9821 | Processive Cellulase | Cellulose | Clostridium cellobioparum | Yes | No | 96.72 | 19.46 | 29.11 | 45.03 | 71.59 | -0.58 | 0.00 | 0.63 | 1.30 |
|  | m.24012 | Processive Cellulase | Cellulose | Cystobacter fuscus | Yes | Yes | 45.57 | 135.80 | 661.50 | 1117.54 | 298.47 | -2.28 | 0.00 | 0.76 | -1.15 |
|  | m.16454 | Processive Cellulase | Cellulose | Acidothermus cellulolyticus | Yes | No | 44.30 | 41.96 | 33.17 | 44.80 | 42.41 | 0.34 | 0.00 | 0.43 | 0.35 |
|  | m.20878 | Processive Cellulase | Cellulose | Clostridium cellobioparum | Yes | Yes | 31.15 | 176.49 | 88.14 | 32.40 | 25.14 | 1.00 | 0.00 | -1.44 | -1.81 |
|  | m.16453 | Processive Cellulase | Cellulose | Acidothermus cellulolyticus | Yes | No | 18.65 | 10.34 | 18.14 | 32.10 | 41.78 | -0.81 | 0.00 | 0.82 | 1.20 |
|  | m.20888 | Processive Cellulase | Cellulose | Clostridium cellobioparum | Yes | Yes | 13.27 | 66.31 | 38.36 | 13.15 | 10.28 | 0.79 | 0.00 | -1.54 | -1.90 |
|  | m.15105 | Processive Cellulase | Cellulose | Caldicellulosiruptor bescii DSM 6725 | Yes | Yes | 5.57 | 0.00 | 0.00 | 0.31 | 0.00 | 0.00 | 0.00 | 18.26 | 0.00 |
|  | m.15107 | Processive Cellulase | Cellulose | Caldicellulosiruptor bescii DSM 6725 | Yes | Yes | 3.47 | 2.48 | 4.97 | 1.36 | 0.87 | -1.00 | 0.00 | -1.87 | -2.51 |
|  | m.19944 | Processive Cellulase | Cellulose | Cystobacter fuscus | Yes | Yes | 3.45 | 21.69 | 13.77 | 5.15 | 54.90 | 0.66 | 0.00 | -1.42 | 2.00 |
|  | m.19952 | Processive Cellulase | Cellulose | Cystobacter fuscus | Yes | Yes | 2.93 | 30.58 | 15.04 | 28.99 | 31.89 | 1.02 | 0.00 | 0.95 | 1.08 |
|  | m.15108 | Processive Cellulase | Cellulose | Caldicellulosiruptor bescii DSM 6725 | Yes | Yes | 0.49 | 0.00 | 1.32 | 0.21 | 0.50 | -20.33 | 0.00 | -2.67 | -1.40 |
|  | m.8113 | Processive Cellulase | Cellulose | Streptomyces | Yes | No | 0.25 | 0.33 | 1.28 | 1.81 | 0.72 | -1.98 | 0.00 | 0.50 | -0.83 |
|  |  |  |  |  |  |  |  |  |  |  |  |  |  |  |  |
| GH53 | m.17920 | endo-β-1,4-galactanase | Pectin | Streptococcus henryi | Yes | NO | 20.28 | 20.08 | 3.68 | 5.07 | 2.74 | 2.45 | 0.00 | 0.46 | -0.43 |
|  | m.25438 | arabinogalactan endo-1 4-beta-galactosidase | Pectin | Ruminococcus flavefaciens | Yes | No | 10.23 | 10.73 | 12.96 | 20.66 | 13.74 | -0.27 | 0.00 | 0.67 | 0.08 |
|  |  |  |  |  |  |  |  |  |  |  |  |  |  |  |  |
| GH55 | m.25294 | beta 1-3 endo/exo glucanase? | Others: β-1,3-glucan (laminarin) | Eubacterium ventriosum | Yes | No | 8.30 | 17.69 | 10.04 | 8.36 | 14.81 | 0.82 | 0.00 | -0.26 | 0.56 |
|  |  |  |  |  |  |  |  |  |  |  |  |  |  |  |  |
| GH57 | m.16044 | Amylase | Starch | Mucor circinelloides f. circinelloides 1006PhL | NO | NO | 113.66 | 307.96 | 188.86 | 190.36 | 191.90 | 0.71 | 0.00 | 0.01 | 0.02 |
|  | m.24342 | Amylase | Starch | Colletotrichum gloeosporioides Nara gc5 | NO | NO | 55.47 | 218.28 | 500.28 | 173.43 | 141.84 | -1.20 | 0.00 | -1.53 | -1.82 |
|  | m.18227 | Amylase | Starch | Clostridium bolteae | Yes | NO | 15.19 | 3.86 | 4.65 | 1.49 | 7.18 | -0.27 | 0.00 | -1.64 | 0.63 |
|  | m.26365 | Amylase | Starch | Rhizopus delemar RA 99-880 | NO | NO | 3.15 | 11.34 | 14.53 | 6.48 | 6.78 | -0.36 | 0.00 | -1.17 | -1.10 |
|  | m.5882 | Amylase | Starch | Dacryopinax sp. DJM-731 SS1 | NO | NO | 1.70 | 0.51 | 0.67 | 5.87 | 2.70 | -0.38 | 0.00 | 3.13 | 2.01 |
|  | m.25791 | Amylase | Starch | Mucor circinelloides f. circinelloides 1006PhL | NO | NO | 1.19 | 0.50 | 4.63 | 5.71 | 7.40 | -3.22 | 0.00 | 0.30 | 0.68 |
|  | m.4169 | Amylase | Starch | Alkaliphilus metalliredigens QYMF | Yes | NO | 0.33 | 1.58 | 0.00 | 0.00 | 0.00 | 20.59 | 0.00 | 0.00 | 0.00 |
|  |  |  |  |  |  |  |  |  |  |  |  |  |  |  |  |
| GH64 | m.14266 | β-1,3-glucanase | Others: β-1,3-glucan (laminarin) | Eubacterium sp. CAG:192 | Yes | NO | 5.93 | 7.72 | 1.91 | 1.30 | 1.01 | 2.01 | 0.00 | -0.56 | -0.92 |
|  | m.14265 | β-1,3-glucanase | Others: β-1,3-glucan (laminarin) | Eubacterium sp. CAG:192 | Yes | NO | 0.67 | 9.73 | 3.99 | 1.67 | 1.81 | 1.29 | 0.00 | -1.25 | -1.14 |
|  |  |  |  |  |  |  |  |  |  |  |  |  |  |  |  |
| GH67 | m.15698 | α-glucuronidase | Hemicellulose: xylan | Bacteroides sp. CAG:1060 | Yes | No | 1.21 | 6.31 | 1.52 | 1.97 | 2.76 | 2.06 | 0.00 | 0.38 | 0.86 |
|  |  |  |  |  |  |  |  |  |  |  |  |  |  |  |  |
| GH74 | m.19667 | xyloglucanase | Hemicellulose: xyloglucan | Amycolatopsis balhimycina | Yes | Yes | 41.51 | 43.21 | 28.14 | 16.45 | 5.23 | 0.62 | 0.00 | -0.77 | -2.43 |
|  | m.19666 | xyloglucanase | Hemicellulose: xyloglucan | Amycolatopsis balhimycina | Yes | Yes | 10.65 | 16.68 | 13.08 | 24.17 | 12.04 | 0.35 | 0.00 | 0.89 | -0.12 |
|  | m.4856 | xyloglucanase | Hemicellulose: xyloglucan | Thermobispora bispora DSM 43833 | Yes | Yes | 5.93 | 28.62 | 12.33 | 10.06 | 7.18 | 1.21 | 0.00 | -0.29 | -0.78 |
|  | m.3912 | Xyloglucanase | Hemicellulose: xyloglucan | Mucor circinelloides f. circinelloides 1006PhL | NO | No | 3.06 | 4.13 | 8.87 | 8.69 | 5.43 | -1.10 | 0.00 | -0.03 | -0.71 |
|  |  |  |  |  |  |  |  |  |  |  |  |  |  |  |  |
| GH78 | m.26096 | alfa-L-rhamnosidase | Pectin | Clostridium sp. CAG:413 | Yes | NO | 18.30 | 17.74 | 4.01 | 2.33 | 3.79 | 2.15 | 0.00 | -0.78 | -0.08 |
|  |  |  |  |  |  |  |  |  |  |  |  |  |  |  |  |
| GH95 | m.4438 | alpha l fucosidase | Hemicellulose: xyloglucan | Bacteroides sp. CAG:633 | Yes | Yes | 44.09 | 38.34 | 20.53 | 15.13 | 35.22 | 0.90 | 0.00 | -0.44 | 0.78 |
|  |  |  |  |  |  |  |  |  |  |  |  |  |  |  |  |
| GH97 | m.2374 | Alpha Glucosidase | Hemicellulose: xyloglucan | Aedes aegypti | NO | NO | 8.09 | 0.56 | 2.11 | 0.60 | 4.31 | -1.91 | 0.00 | -1.81 | 1.03 |
|  | m.20593 | Alpha Glucosidase | Hemicellulose: xyloglucan | Trichomonas vaginalis G3 | NO | NO | 0.31 | 1.36 | 2.61 | 0.35 | 0.54 | -0.95 | 0.00 | -2.92 | -2.27 |
|  |  |  |  |  |  |  |  |  |  |  |  |  |  |  |  |
| GH105 | m.3937 | unsaturated rhamnogalacturonyl hydrolase | Pectin | Clostridium sp. CAG:307 | Yes | NO | 39.08 | 46.91 | 21.24 | 13.53 | 28.87 | 1.14 | 0.00 | -0.65 | 0.44 |
|  |  |  |  |  |  |  |  |  |  |  |  |  |  |  |  |
| GH109 | m.25478 | α-N-acetylgalactosaminidase | Other (polygalactosamine) | Mucor circinelloides f. circinelloides 1006PhL | NO | NO | 4.41 | 6.25 | 18.91 | 14.98 | 11.76 | -1.60 | 0.00 | -0.34 | -0.68 |
|  | m.27785 | α-N-acetylgalactosaminidase | Other (polygalactosamine) | Paenibacillus vortex | Yes | NO | 3.29 | 2.42 | 2.44 | 2.83 | 1.99 | -0.02 | 0.00 | 0.21 | -0.30 |
|  | m.27690 | α-N-acetylgalactosaminidase | Other (polygalactosamine) | Paenibacillus barengoltzii | Yes | NO | 0.26 | 1.94 | 1.02 | 0.14 | 0.18 | 0.93 | 0.00 | -2.88 | -2.50 |
|  |  |  |  |  |  |  |  |  |  |  |  |  |  |  |  |
| GH114 | m.25001 | endo alpha-1,4 polygalactosaminidase | Other (polygalactosamine) | Hahella ganghwensis | Yes | NO | 36.88 | 52.07 | 34.30 | 20.67 | 18.15 | 0.60 | 0.00 | -0.73 | -0.92 |
|  | m.2602 | endo alpha-1,4 polygalactosaminidase | Other (polygalactosamine) | Marinobacter nanhaiticus | Yes | NO | 12.22 | 184.06 | 127.38 | 39.45 | 59.37 | 0.53 | 0.00 | -1.69 | -1.10 |
|  | m.2133 | endo alpha-1,4 polygalactosaminidase | Other (polygalactosamine) | Marinobacter nanhaiticus | Yes | NO | 11.05 | 1.56 | 0.52 | 0.26 | 0.44 | 1.58 | 0.00 | -1.01 | -0.25 |
|  | m.26281 | endo alpha-1,4 polygalactosaminidase | Other (polygalactosamine) | uncultured bacterium | Yes | NO | 6.89 | 50.43 | 5.42 | 2.23 | 1.93 | 3.22 | 0.00 | -1.28 | -1.49 |
|  | m.17212 | endo alpha-1,4 polygalactosaminidase | Other (polygalactosamine) | uncultured bacterium | Yes | NO | 3.44 | 16.79 | 6.84 | 8.42 | 10.80 | 1.30 | 0.00 | 0.30 | 0.66 |
|  | m.17209 | endo alpha-1,4 polygalactosaminidase | Other (polygalactosamine) | Idiomarina loihiensis L2TR | Yes | NO | 3.10 | 14.58 | 5.26 | 7.29 | 6.50 | 1.47 | 0.00 | 0.47 | 0.31 |
|  | m.2134 | endo alpha-1,4 polygalactosaminidase | Other (polygalactosamine) | Idiomarina loihiensis L2TR | Yes | NO | 2.88 | 0.65 | 0.61 | 0.16 | 0.46 | 0.10 | 0.00 | -1.94 | -0.40 |
|  | m.26295 | endo alpha-1,4 polygalactosaminidase | Other (polygalactosamine) | uncultured bacterium | Yes | NO | 2.35 | 16.90 | 4.71 | 3.07 | 6.73 | 1.84 | 0.00 | -0.62 | 0.52 |
|  | m.803 | endo alpha-1,4 polygalactosaminidase | Other (polygalactosamine) | uncultured bacterium | Yes | NO | 1.84 | 3.16 | 4.65 | 25.70 | 4.07 | -0.56 | 0.00 | 2.47 | -0.19 |
|  | m.10032 | endo alpha-1,4 polygalactosaminidase | Other (polygalactosamine) | None | None | NO | 1.61 | 2.87 | 2.96 | 0.43 | 1.30 | -0.04 | 0.00 | -2.79 | -1.19 |
|  | m.5242 | endo alpha-1,4 polygalactosaminidase | Other (polygalactosamine) | Meyerozyma guilliermondii ATCC 6260 | NO | NO | 0.97 | 1.60 | 1.10 | 1.52 | 0.89 | 0.54 | 0.00 | 0.46 | -0.31 |
|  | m.28062 | endo alpha-1,4 polygalactosaminidase | Other (polygalactosamine) | Idiomarina loihiensis L2TR | Yes | NO | 0.93 | 2.80 | 3.18 | 0.81 | 0.74 | -0.18 | 0.00 | -1.98 | -2.10 |
|  | m.26120 | endo alpha-1,4 polygalactosaminidase | Other (polygalactosamine) | Marinobacter nanhaiticus | Yes | NO | 0.58 | 0.18 | 1.15 | 0.98 | 1.82 | -2.69 | 0.00 | -0.24 | 0.66 |
|  | m.17210 | endo alpha-1,4 polygalactosaminidase | Other (polygalactosamine) | uncultured bacterium | Yes | NO | 0.52 | 7.44 | 3.22 | 0.49 | 0.82 | 1.21 | 0.00 | -2.72 | -1.97 |
|  | m.10031 | endo alpha-1,4 polygalactosaminidase | Other (polygalactosamine) | uncultured bacterium | Yes | NO | 0.00 | 1.22 | 0.90 | 0.10 | 0.95 | 0.44 | 0.00 | -3.16 | 0.08 |
|  | m.8740 | putative endo alpha-1,4 polygalactosaminidase | Other (polygalactosamine) | Endoriftia persephone | Yes | NO | 0.00 | 0.19 | 0.76 | 1.29 | 1.17 | -1.99 | 0.00 | 0.77 | 0.63 |
|  |  |  |  |  |  |  |  |  |  |  |  |  |  |  |  |
| GH115 | m.9664 | xylan α-1,2-glucuronidase | Hemicellulose: xylan accessory | Paenibacillus sp. HW567 | Yes | Yes | 101.91 | 21.24 | 6.08 | 2.14 | 4.65 | 1.80 | 0.00 | -1.51 | -0.39 |
|  | m.11352 | Alpha Glucordianse | Hemicellulose: xylan accessory | Clostridium sp. BNL1100 | Yes | No | 5.47 | 5.01 | 0.42 | 0.11 | 0.48 | 3.59 | 0.00 | -1.86 | 0.19 |
|  | m.19215 | Alpha Glucordianse | Hemicellulose: xylan accessory | Roseburia sp. CAG:309 | Yes | No | 2.18 | 0.12 | 0.22 | 0.08 | 0.29 | -0.84 | 0.00 | -1.45 | 0.37 |
|  | m.15104 | Alpha Glucordianse | Hemicellulose: xylan accessory | Clostridium sp. BNL1100 | Yes | No | 1.61 | 0.30 | 0.17 | 0.07 | 0.30 | 0.82 | 0.00 | -1.36 | 0.82 |
|  |  |  |  |  |  |  |  |  |  |  |  |  |  |  |  |
| GH119 | m.24176 | alpha amylase | Starch | Lactobacillus manihotivorans | Yes | NO | 943.14 | 270.01 | 146.52 | 27.69 | 48.56 | 0.88 | 0.00 | -2.40 | -1.59 |
|  | m.16888 | alpha amylase | Starch | Lactobacillus manihotivorans | Yes | NO | 789.36 | 36.82 | 121.82 | 90.49 | 46.63 | -1.73 | 0.00 | -0.43 | -1.39 |
|  | m.24163 | alpha amylase | Starch | Clostridium sp. CAG:411 | Yes | NO | 539.76 | 69.52 | 148.39 | 229.72 | 85.53 | -1.09 | 0.00 | 0.63 | -0.79 |
|  | m.24304 | alpha amylase | Starch | Butyrivibrio sp. CAG:318 | Yes | NO | 536.76 | 313.02 | 261.29 | 137.32 | 125.99 | 0.26 | 0.00 | -0.93 | -1.05 |
|  | m.6241 | alpha amylase | Starch | Lactobacillus manihotivorans | Yes | NO | 192.43 | 20.46 | 20.64 | 3.98 | 4.36 | -0.01 | 0.00 | -2.38 | -2.24 |
|  | m.5073 | alpha amylase | Starch | Lactobacillus manihotivorans | Yes | NO | 140.76 | 40.65 | 53.19 | 140.42 | 63.97 | -0.39 | 0.00 | 1.40 | 0.27 |
|  | m.25077 | alpha amylase | Starch | Kocuria varians | Yes | NO | 132.33 | 48.11 | 40.68 | 37.44 | 37.40 | 0.24 | 0.00 | -0.12 | -0.12 |
|  | m.22769 | alpha amylase | Starch | Clostridium sp. CAG:411 | Yes | NO | 114.89 | 37.52 | 75.89 | 37.36 | 37.23 | -1.02 | 0.00 | -1.02 | -1.03 |
|  | m.9619 | alpha amylase | Starch | Bacillus halodurans | Yes | NO | 94.01 | 14.66 | 17.59 | 37.63 | 32.12 | -0.26 | 0.00 | 1.10 | 0.87 |
|  | m.24827 | alpha amylase | Starch | Clostridium saccharoperbutylacetonicum N1-4(HMT) | Yes | NO | 75.69 | 25.71 | 13.37 | 0.12 | 0.45 | 0.94 | 0.00 | -6.80 | -4.90 |
|  | m.22773 | alpha amylase | Starch | Clostridium sp. CAG:411 | Yes | NO | 48.97 | 12.29 | 1.68 | 2.43 | 0.55 | 2.87 | 0.00 | 0.53 | -1.62 |
|  | m.19380 | alpha amylase | Starch | Bacillus sp. | Yes | NO | 21.79 | 7.84 | 2.64 | 1.08 | 2.54 | 1.57 | 0.00 | -1.29 | -0.06 |
|  | m.24911 | α-amylase | Starch | Clostridium sp. CAG:411 | Yes | NO | 20.03 | 2.23 | 4.81 | 1.08 | 0.66 | -1.11 | 0.00 | -2.16 | -2.87 |
|  | m.22762 | alpha amylase | Starch | Clostridium sp. CAG:411 | Yes | NO | 16.32 | 4.83 | 0.00 | 0.59 | 0.10 | 22.20 | 0.00 | 19.17 | 16.63 |
|  | m.754 | alpha amylase | Starch | Streptococcus gallolyticus | Yes | NO | 10.84 | 25.00 | 4.21 | 5.31 | 4.66 | 2.57 | 0.00 | 0.33 | 0.14 |
|  | m.5089 | alpha amylase | Starch | Clostridium sp. CAG:411 | Yes | NO | 9.56 | 2.62 | 2.37 | 0.17 | 0.38 | 0.14 | 0.00 | -3.83 | -2.65 |
|  | m.22757 | alpha amylase | Starch | Clostridium sp. CAG:411 | Yes | NO | 5.32 | 1.18 | 0.46 | 0.62 | 0.25 | 1.37 | 0.00 | 0.44 | -0.89 |
|  | m.27983 | alpha amylase | Starch | Clostridium sp. CAG:411 | Yes | NO | 4.92 | 1.59 | 0.65 | 0.00 | 0.09 | 1.29 | 0.00 | -19.31 | -2.77 |
|  | m.3021 | alpha amylase | Starch | Trichomonas vaginalis G3 | NO | NO | 1.33 | 0.47 | 1.62 | 0.62 | 0.61 | -1.78 | 0.00 | -1.39 | -1.42 |
|  | m.9656 | alpha amylase | Starch | Bacillus halodurans C-125 | Yes | NO | 1.04 | 4.53 | 1.99 | 1.94 | 1.15 | 1.19 | 0.00 | -0.03 | -0.79 |
|  |  |  |  |  |  |  |  |  |  |  |  |  |  |  |  |
| GH124 | m.5611 | Endoglucanase | Cellulose | Acetivibrio cellulolyticus | Yes | No | 41.26 | 40.10 | 42.11 | 57.66 | 37.00 | -0.07 | 0.00 | 0.45 | -0.19 |
|  | m.22184 | Endoglucanase | Cellulose | Ruminococcus flavefaciens | Yes | Yes | 38.86 | 41.57 | 56.33 | 84.56 | 45.21 | -0.44 | 0.00 | 0.59 | -0.32 |
|  | m.5613 | Unknown | Cellulose | None | Uknown | No | 4.97 | 6.24 | 13.82 | 21.05 | 15.69 | -1.15 | 0.00 | 0.61 | 0.18 |
|  |  |  |  |  |  |  |  |  |  |  |  |  |  |  |  |
| PL1 | m.5744 |  | Pectin | Rattus norvegicus GN | NO | No | 109.78 | 111.12 | 105.66 | 71.38 | 53.94 | 0.07 | 0.00 | -0.57 | -0.97 |
|  | m.790 |  | Pectin | Emericella nidulans | NO | No | 31.96 | 32.49 | 8.37 | 5.38 | 9.59 | 1.96 | 0.00 | -0.64 | 0.20 |
|  | m.791 |  | Pectin | Neosartorya fischeri | NO | No | 13.42 | 8.40 | 4.33 | 3.21 | 5.77 | 0.96 | 0.00 | -0.43 | 0.41 |
|  | m.10293 |  | Pectin | Mus musculus GN | NO | No | 4.74 | 0.13 | 2.45 | 1.39 | 0.50 | -4.24 | 0.00 | -0.81 | -2.30 |
|  | m.10191 |  | Pectin | Bacillus subtilis (strain 168) | Yes | No | 4.35 | 38.28 | 35.24 | 13.69 | 16.18 | 0.12 | 0.00 | -1.36 | -1.12 |
|  | m.21303 |  | Pectin | Emericella nidulans | Yes | No | 4.15 | 25.43 | 6.05 | 3.92 | 1.09 | 2.07 | 0.00 | -0.63 | -2.47 |
|  | m.21302 |  | Pectin | Emericella nidulans | Yes | No | 3.64 | 17.72 | 1.74 | 2.26 | 0.53 | 3.35 | 0.00 | 0.38 | -1.72 |
|  | m.1842 |  | Pectin | Aspergillus flavus | NO | No | 2.93 | 16.68 | 4.10 | 3.73 | 3.46 | 2.02 | 0.00 | -0.14 | -0.25 |
|  | m.21306 |  | Pectin | Erwinia chrysanthemi GN | Yes | No | 1.81 | 4.87 | 0.93 | 3.29 | 0.61 | 2.38 | 0.00 | 1.82 | -0.61 |
|  | m.21807 |  | Pectin | Bacillus subtilis (strain 168) | Yes | No | 1.70 | 3.21 | 2.95 | 2.60 | 1.30 | 0.13 | 0.00 | -0.18 | -1.18 |
|  | m.9955 |  | Pectin | Emericella nidulans | NO | No | 1.67 | 43.79 | 34.51 | 12.16 | 12.05 | 0.34 | 0.00 | -1.51 | -1.52 |
|  | m.21307 |  | Pectin | Emericella nidulans | NO | No | 1.34 | 1.73 | 2.82 | 1.09 | 0.70 | -0.71 | 0.00 | -1.37 | -2.00 |
|  | m.21310 |  | Pectin | Emericella nidulans | NO | No | 0.98 | 9.14 | 1.95 | 1.60 | 0.50 | 2.23 | 0.00 | -0.29 | -1.96 |
|  | m.10190 |  | Pectin | Bacillus subtilis (strain 168) | Yes | No | 0.57 | 7.78 | 5.43 | 1.33 | 1.74 | 0.52 | 0.00 | -2.03 | -1.64 |
|  | m.21305 |  | Pectin | Emericella nidulans | Yes | No | 0.55 | 4.88 | 1.21 | 1.22 | 0.48 | 2.01 | 0.00 | 0.02 | -1.35 |
|  | m.9954 |  | Pectin | Aspergillus niger | NO | No | 0.46 | 1.76 | 1.35 | 0.22 | 0.46 | 0.38 | 0.00 | -2.62 | -1.56 |
|  | m.1591 |  | Pectin | Bacillus subtilis (strain 168) | Yes | No | 0.13 | 2.72 | 0.89 | 0.02 | 0.06 | 1.60 | 0.00 | -5.88 | -3.99 |
|  | m.21311 |  | Pectin | Emericella nidulans | NO | No | 0.00 | 14.65 | 4.38 | 1.15 | 1.45 | 1.74 | 0.00 | -1.93 | -1.59 |
|  |  |  |  |  |  |  |  |  |  |  |  |  |  |  |  |
| PL2 | m.9251 |  | Pectin | Alicyclobacillus acidocaldarius subsp. Acidocaldarius | Yes | No | 2.67 | 0.65 | 3.88 | 3.58 | 2.46 | -2.58 | 0.00 | -0.12 | -0.66 |
|  |  |  |  |  |  |  |  |  |  |  |  |  |  |  |  |
| PL3 | m.20357 | pectate lyase | Pectin | Neosartorya fumigata | NO | No | 27.06 | 35.20 | 15.60 | 35.15 | 6.21 | 1.17 | 0.00 | 1.17 | -1.33 |
|  | m.20359 | pectate lyase | Pectin | Neosartorya fumigata | NO | No | 14.91 | 14.27 | 1.11 | 0.10 | 2.10 | 3.68 | 0.00 | -3.46 | 0.92 |
|  | m.19796 | pectate lyase | Pectin | Homo sapiens GN | NO | No | 3.38 | 49.65 | 1.85 | 14.72 | 0.88 | 4.75 | 0.00 | 2.99 | -1.06 |
|  | m.25296 | pectate lyase | Pectin | Aspergillus terreus | NO | No | 3.28 | 25.73 | 11.51 | 9.34 | 8.69 | 1.16 | 0.00 | -0.30 | -0.41 |
|  | m.20358 | pectate lyase | Pectin | Homo sapiens GN | NO | No | 2.36 | 5.87 | 1.27 | 1.93 | 2.02 | 2.21 | 0.00 | 0.60 | 0.67 |
|  | m.20355 | pectate lyase | Pectin | Homo sapiens GN | NO | No | 2.26 | 12.02 | 1.88 | 0.18 | 3.84 | 2.68 | 0.00 | -3.36 | 1.03 |
|  | m.20361 | pectate lyase | Pectin | Aspergillus clavatus | NO | No | 2.05 | 13.74 | 5.36 | 6.85 | 1.09 | 1.36 | 0.00 | 0.35 | -2.29 |
|  | m.2275 | pectate lyase | Pectin | Homo sapiens GN | NO | No | 1.71 | 17.26 | 4.51 | 1.09 | 1.52 | 1.94 | 0.00 | -2.05 | -1.57 |
|  | m.2276 | pectate lyase | Pectin | Schizosaccharomyces pombe | NO | No | 0.00 | 2.89 | 1.30 | 0.89 | 1.19 | 1.16 | 0.00 | -0.55 | -0.13 |
|  |  |  |  |  |  |  |  |  |  |  |  |  |  |  |  |
| PL4 | m.20907 | rhamnogalacturonan lyase | Pectin | Aspergillus terreus | NO | Yes | 56.81 | 42.22 | 61.24 | 67.26 | 108.01 | -0.54 | 0.00 | 0.14 | 0.82 |
|  | m.889 | rhamnogalacturonan lyase | Pectin | Emericella nidulans | NO | No | 6.38 | 9.39 | 7.59 | 8.21 | 15.99 | 0.31 | 0.00 | 0.11 | 1.07 |
|  | m.10847 | rhamnogalacturonan lyase | Pectin | Aspergillus terreus | NO | No | 2.33 | 3.06 | 3.64 | 4.87 | 4.22 | -0.25 | 0.00 | 0.42 | 0.21 |
|  | m.10846 | rhamnogalacturonan lyase | Pectin | Aspergillus terreus | NO | No | 1.04 | 0.55 | 1.16 | 1.65 | 1.65 | -1.07 | 0.00 | 0.50 | 0.50 |
|  |  |  |  |  |  |  |  |  |  |  |  |  |  |  |  |
| PL9 | m.836 |  | Pectin | Aspergillus terreus | NO | No | 11.07 | 28.13 | 5.91 | 2.93 | 7.12 | 2.25 | 0.00 | -1.02 | 0.27 |
| PL10 | m.13639 | pectate lyase | Pectin | Aspergillus clavatus | Eukaroytic | No | 10.55 | 15.21 | 18.56 | 19.44 | 11.02 | -0.29 | 0.00 | 0.07 | -0.75 |
|  |  |  |  |  |  |  |  |  |  |  |  |  |  |  |  |
| PL11 | m.4447 |  | Pectin | Aspergillus terreus | NO | No | 1.15 | 0.35 | 1.53 | 1.45 | 1.24 | -2.12 | 0.00 | -0.08 | -0.30 |
|  |  |  |  |  |  |  |  |  |  |  |  |  |  |  |  |
| PL22 | m.25214 | oligogalacturonide lyases | Pectin | Bacillus subtilis | Yes | No | 56.76 | 38.89 | 16.96 | 0.90 | 17.93 | 1.20 | 0.00 | -4.24 | 0.08 |
|  | m.9007 | oligogalacturonide lyases | Pectin | Dickeya dadantii | Yes | No | 25.41 | 1.27 | 7.78 | 9.97 | 5.74 | -2.61 | 0.00 | 0.36 | -0.44 |
|  | m.26768 | oligogalacturonide lyases | Pectin | Dickeya dadantii | Yes | No | 6.43 | 1.14 | 4.02 | 4.12 | 2.44 | -1.82 | 0.00 | 0.04 | -0.72 |
|  | m.15411 | oligogalacturonide lyases | Pectin | Dickeya dadantii | Yes | No | 0.46 | 0.00 | 0.38 | 1.01 | 1.09 | -18.52 | 0.00 | 1.42 | 1.53 |
|  | m.11211 | oligogalacturonide lyases | Pectin | Dickeya dadantii | Yes | No | 0.32 | 0.29 | 2.10 | 0.49 | 0.47 | -2.84 | 0.00 | -2.09 | -2.16 |
|  |  |  |  |  |  |  |  |  |  |  |  |  |  |  |  |

a: Corrected FPKM values normalized by the library size, as calculated using the estimateDispersions function in the R package DESeq.

b: Fold change is shown as Log_2_ expression levels compared to glucose. Color code: Green= significantly over-expressed (a differential expression p-value <0. 1 as calculated by the nbinomTest function in the R package DESeq), red= significantly under-expressed (a differential expression p-value <0.1 as calculated by the nbinomTest function in the R package DESeq).

**Table S2. Transcription levels of all CE transcripts when grown on glucose (Glu) and lignocellulosic biomass (Alfalfa, Alf; Energy Cane, EC; Corn Stover, CS; Sorghum, Sor) substrates.**

| Gene family | Transcript ID | Activity | Phylogeny | Bacterial/HGT | Log_2_ transcription level (log_2_(ratio FPKM biomass: FPKM glucose)^a^ | | | | Average Absolute FPKM (corrected values) ^b^ | | | | |
| --- | --- | --- | --- | --- | --- | --- | --- | --- | --- | --- | --- | --- | --- |
|  |  |  |  |  | Alfalfa | Energy Cane | Corn Stover | Sorghum | Glucose | Alfalfa | Energy Cane | Corn Stover | Sorghum |
| CE1 | m.10376 | Feruloyl Esterase A | Ruminococcus albus SY3 | Yes | 2.02 | -1.21 | -0.39 | -2.30 | 20.79 | 84.16 | 9.00 | 15.92 | 4.22 |
| CE1 | m.10377 | Feruloyl Esterase A | Ruminococcus sp. CAG:624 | Yes | 2.91 | 1.26 | 0.44 | -0.40 | 7.94 | 59.64 | 18.99 | 10.75 | 6.00 |
| CE1 | m.10494 | Feruloyl Esterase A | Clostridium thermocellum | Yes | 4.04 | 2.18 | -18.81 | -3.48 | 0.46 | 7.57 | 2.09 | 0.00 | 0.04 |
| CE1 | m.10495 | Feruloyl Esterase A | Clostridium thermocellum | Yes | 21.69 | 19.38 | 15.41 | 15.20 | 0.00 | 3.39 | 0.68 | 0.04 | 0.04 |
| CE1 | m.10732 | Uknown most likley non Plant cell wall decompostion | Mucor circinelloides f. circinelloides 1006PhL | No | -0.04 | 1.56 | 1.14 | 0.43 | 2.52 | 2.44 | 7.43 | 5.55 | 3.39 |
| CE1 | m.11472 | Feruloyl Esterase A | Clostridium straminisolvens JCM 21531 | Yes | 21.57 | 18.30 | 17.51 | 16.98 | 0.00 | 3.11 | 0.32 | 0.19 | 0.13 |
| CE1 | m.14195 | Alpha/Beta Hydrolase | Ruminococcus flavefaciens 007c | Yes | -1.19 | -0.72 | -0.89 | -0.26 | 63.43 | 27.88 | 38.38 | 34.30 | 53.07 |
| CE1 | m.14365 | Unknown | Eubacterium plexicaudatum | Yes | 2.94 | 2.21 | 0.72 | 0.96 | 6.48 | 49.81 | 29.94 | 10.70 | 12.64 |
| CE1 | m.14696 | Unknown | Bacillus subtilis (strain 168) | Yes | -4.23 | -0.09 | -2.54 | -1.88 | 1.58 | 0.08 | 1.48 | 0.27 | 0.43 |
| CE1 | m.1547 | Feruloyl Esterase A | Clostridium thermocellum | Yes | 2.59 | 3.77 | 1.58 | 2.90 | 0.10 | 0.59 | 1.33 | 0.29 | 0.73 |
| CE1 | m.16513 | Unknown | Eubacterium plexicaudatum | Yes | -1.42 | -1.68 | -2.52 | -0.96 | 12.73 | 4.76 | 3.97 | 2.22 | 6.56 |
| CE1 | m.1699 | Feruloyl Esterase A | Anaeromyces mucronatus | Yes | 21.03 | 19.19 | 16.61 | 15.46 | 0.00 | 2.14 | 0.60 | 0.10 | 0.04 |
| CE1 | m.17658 | Alpha/Beta Hydrolase | Ruminococcus flavefaciens 007c | Yes | -5.28 | -6.97 | -6.66 | -7.72 | 4.41 | 0.11 | 0.04 | 0.04 | 0.02 |
| CE1 | m.18232 | Unknown Esterase/Lipase GH10 | Salinimonas chungwhensis | Yes | 3.05 | 1.25 | -4.45 | -2.53 | 0.58 | 4.83 | 1.38 | 0.03 | 0.10 |
| CE1 | m.18401 | Feruloyl Esterase A | Clostridium thermocellum | Yes | 1.05 | -1.67 | -2.61 | -0.85 | 41.17 | 85.45 | 12.93 | 6.72 | 22.85 |
| CE1 | m.18404 | Feruloyl Esterase A | Clostridium thermocellum | Yes | -0.04 | 0.27 | -0.50 | 1.44 | 18.89 | 18.37 | 22.76 | 13.35 | 51.39 |
| CE1 | m.18561 | Ferulyoyl Esterase | Fibrobacter succinogenes subsp. succinogenes S85 | Yes | 0.20 | -5.28 | -24.65 | -1.00 | 26.25 | 30.10 | 0.67 | 0.00 | 13.13 |
| CE1 | m.18564 | Feruloyl Esterase C | Fibrobacter succinogenes subsp. succinogenes S85 | Yes | -1.12 | -3.39 | -4.79 | -2.42 | 45.64 | 20.98 | 4.34 | 1.65 | 8.50 |
| CE1 | m.18566 | Feruloyl Esterase C | Fibrobacter succinogenes subsp. succinogenes S85 | Yes | -2.05 | -3.63 | -7.51 | -2.75 | 79.42 | 19.20 | 6.42 | 0.44 | 11.78 |
| CE1 | m.18570 | Feruloyl Esterase C | Fibrobacter succinogenes subsp. succinogenes S85 | Yes | 2.66 | 2.74 | -0.30 | 2.70 | 0.37 | 2.37 | 2.50 | 0.30 | 2.43 |
| CE1 | m.18574 | Ferulyoyl Esterase | Fibrobacter succinogenes subsp. succinogenes S85 | Yes | -0.72 | -5.96 | -4.64 | -3.28 | 209.37 | 127.28 | 3.37 | 8.37 | 21.53 |
| CE1 | m.18577 | Ferulyoyl Esterase | Fibrobacter succinogenes subsp. succinogenes S85 | Yes | 0.94 | -0.40 | -0.94 | -0.74 | 79.59 | 152.86 | 60.19 | 41.35 | 47.49 |
| CE1 | m.19386 | PhosphoLipase | Escherichia coli (strain K12) | Yes | 2.43 | 2.35 | 1.75 | 0.98 | 10.64 | 57.45 | 54.09 | 35.88 | 21.02 |
| CE1 | m.20174 | Ferulyoyl Esterase | Piromyces equi | No | 2.39 | 3.35 | 3.91 | 5.16 | 1.45 | 7.62 | 14.76 | 21.79 | 51.80 |
| CE1 | m.2046 | Feruloyl Esterase A | Clostridium thermocellum | Yes | -3.78 | -0.16 | -2.05 | 2.19 | 4.19 | 0.30 | 3.76 | 1.01 | 19.12 |
| CE1 | m.2075 | Feruloyl Esterase A | Clostridium thermocellum | Yes | 4.82 | 4.25 | 2.80 | 3.92 | 0.16 | 4.58 | 3.08 | 1.13 | 2.47 |
| CE1 | m.21406 | Feruloyl Esterase A | Clostridium thermocellum | Yes | -3.14 | -1.94 | -1.05 | -2.52 | 12.45 | 1.41 | 3.23 | 6.02 | 2.17 |
| CE1 | m.21544 | Feruloyl Esterase C | Fibrobacter succinogenes subsp. succinogenes S85 | Yes | 5.41 | 2.15 | 3.22 | 4.38 | 0.34 | 14.42 | 1.50 | 3.16 | 7.03 |
| CE1 | m.23609 | Lipase | Streptococcus gallolyticus UCN34 | Yes | 2.78 | 1.94 | 2.51 | 2.27 | 4.67 | 32.13 | 17.93 | 26.67 | 22.50 |
| CE1 | m.23613 | Lipase | Streptococcus gallolyticus subsp. gallolyticus ATCC BAA-2069 | Yes | 3.22 | -20.00 | -20.00 | -20.00 | 1.05 | 9.81 | 0.00 | 0.00 | 0.00 |
| CE1 | m.24524 | Uknown Alpha/Beta Hydrolase | Ruminococcus flavefaciens 007c | Yes | -1.45 | -0.65 | 0.42 | -0.14 | 84.85 | 30.96 | 54.26 | 113.67 | 76.84 |
| CE1 | m.25168 | Uknown Alpha/Beta Hydrolase | Eubacterium ramulus | Yes | -0.96 | -1.50 | -1.85 | -1.31 | 50.62 | 26.05 | 17.84 | 14.03 | 20.39 |
| CE1 | m.25177 | Ferulyoyl Esterase | Fibrobacter succinogenes subsp. succinogenes S85 | Yes | 4.81 | 0.92 | 1.26 | 2.53 | 4.02 | 113.00 | 7.61 | 9.63 | 23.24 |
| CE1 | m.25204 | Alpha/Beta Hydrolase | Lachnobacterium bovis | Yes | -4.32 | -2.55 | -1.29 | -2.11 | 127.58 | 6.40 | 21.77 | 52.02 | 29.62 |
| CE1 | m.26011 | Uknown Alpha/Beta Hydrolase | Clostridiales | Yes | 0.95 | 1.60 | 0.15 | 2.79 | 3.72 | 7.19 | 11.29 | 4.11 | 25.68 |
| CE1 | m.26077 | Uknown most likley non Plant cell wall decompostion | Rhizophagus irregularis DAOM 181602 | No | -4.62 | -0.95 | 2.65 | 3.04 | 2.02 | 0.08 | 1.05 | 12.70 | 16.61 |
| CE1 | m.26490 | Feruloyl Esterase A | Fibrobacter succinogenes subsp. succinogenes S85 | Yes | -0.78 | 0.59 | -0.96 | 2.31 | 3.57 | 2.08 | 5.39 | 1.84 | 17.74 |
| CE1 | m.26532 | Uknown | Dialister succinatiphilus | Yes | -1.86 | -2.06 | -2.31 | -3.36 | 12.97 | 3.58 | 3.11 | 2.61 | 1.26 |
| CE1 | m.26813 | Lipase | Slackia heliotrinireducens DSM 20476 | Yes | -2.09 | -2.45 | -1.83 | -3.36 | 22.36 | 5.25 | 4.10 | 6.27 | 2.18 |
| CE1 | m.27418 | Feruloyl Esterase A | Clostridium thermocellum | Yes | 1.64 | 0.97 | 0.88 | 0.99 | 1.46 | 4.55 | 2.85 | 2.68 | 2.88 |
| CE1 | m.27596 | Alpha/Beta Hydrolase | Ruminococcus flavefaciens 007c | Yes | -5.90 | -3.26 | -2.17 | -3.38 | 6.58 | 0.11 | 0.69 | 1.46 | 0.63 |
| CE1 | m.27634 | Unknown | Eubacterium plexicaudatum | Yes | 0.31 | 1.26 | 1.78 | 2.01 | 0.82 | 1.02 | 1.96 | 2.81 | 3.30 |
| CE1 | m.27862 | Alpha/Beta Hydrolase | Firmicutes bacterium CAG:555 | Yes | -2.75 | -0.98 | -0.38 | -1.66 | 5.12 | 0.76 | 2.60 | 3.93 | 1.62 |
| CE1 | m.3108 | Ferulyoyl Esterase | Piromyces equi | No | 1.13 | 1.58 | 1.82 | 2.26 | 34.47 | 75.27 | 103.09 | 122.04 | 165.46 |
| CE1 | m.3122 | Lipase | Clostridium sp. CAG:265 | Yes | 2.53 | 2.61 | 0.34 | 0.57 | 0.24 | 1.41 | 1.49 | 0.31 | 0.36 |
| CE1 | m.5529 | Feruloyl Esterase A | Clostridium thermocellum | Yes | 3.88 | 0.86 | 0.20 | 1.53 | 1.64 | 24.14 | 2.99 | 1.89 | 4.75 |
| CE1 | m.5531 | Feruloyl Esterase A | Clostridium thermocellum | Yes | 4.30 | 2.59 | 1.86 | 4.19 | 0.19 | 3.69 | 1.13 | 0.68 | 3.42 |
| CE1 | m.5654 | AcetylXylan Esterase | Streptococcus criceti | Yes | -3.32 | -2.48 | -1.64 | -2.83 | 5.03 | 0.50 | 0.90 | 1.61 | 0.71 |
| CE1 | m.6140 | Feruloyl Esterase A | Clostridium thermocellum | Yes | 4.82 | 4.14 | 3.45 | 3.09 | 0.81 | 22.95 | 14.28 | 8.83 | 6.88 |
| CE1 | m.7837 | Lipase | Psychrobacter immobilis | Yes | -21.13 | -5.93 | -21.13 | -6.52 | 2.30 | 0.00 | 0.04 | 0.00 | 0.03 |
| CE1 | m.9698 | Feruloyl Esterase A | Clostridium thermocellum | Yes | 0.97 | -1.02 | -3.48 | -3.69 | 13.85 | 27.21 | 6.84 | 1.24 | 1.07 |
| CE1 | m.9699 | Feruloyl Esterase A | Clostridium thermocellum | Yes | 3.73 | 3.18 | 1.61 | 1.10 | 0.21 | 2.83 | 1.93 | 0.65 | 0.46 |
| CE1 | m.9700 | Feruloyl Esterase A | Clostridium thermocellum | Yes | 2.32 | 1.85 | -0.22 | -0.23 | 4.08 | 20.36 | 14.69 | 3.50 | 3.47 |
| CE1 | m.9713 | Ferulyoyl Esterase | Piromyces equi | No | 2.33 | 3.25 | -22.55 | 6.57 | 6.15 | 30.91 | 58.32 | 0.00 | 582.59 |
| CE1 | m.9722 | Ferulyoyl Esterase | Piromyces equi | No | 2.59 | 4.51 | 4.40 | 6.20 | 6.86 | 41.36 | 156.16 | 144.54 | 503.99 |
| CE1 | m.10734 | Unknown Most likely not Plant Cell Wall decompostion | Rhizophagus irregularis DAOM 197198w | No | -0.45 | 0.15 | -0.24 | -0.50 | 4.49 | 3.28 | 5.00 | 3.81 | 3.17 |
| CE1 | m.11062 | Feruloyl Esterase A | Clostridium thermocellum | Yes | 2.25 | 2.29 | -2.00 | 2.67 | 0.16 | 0.76 | 0.78 | 0.04 | 1.02 |
| CE1 | m.1437 | PhosphoLipase | Schizosaccharomyces pombe | No | -0.30 | -0.42 | 0.14 | -0.05 | 1.25 | 1.02 | 0.94 | 1.37 | 1.21 |
| CE1 | m.16514 | Unknown | Eubacterium plexicaudatum | Yes | 0.56 | 0.21 | 0.09 | 0.72 | 7.04 | 10.41 | 8.14 | 7.49 | 11.58 |
| CE1 | m.1682 | Unknown | Rhizophagus irregularis DAOM 197198w | No | -0.79 | 0.80 | 0.96 | 0.07 | 3.52 | 2.03 | 6.12 | 6.86 | 3.69 |
| CE1 | m.17653 | Uknown Alpha/Beta Hydrolase | Lachnospiraceae bacterium A4 | Yes | -0.51 | 0.58 | -0.68 | 1.04 | 2.77 | 1.94 | 4.14 | 1.73 | 5.68 |
| CE1 | m.20913 | Feruloyl Esterase A | Clostridium thermocellum | Yes | -1.29 | -0.07 | -0.02 | -0.94 | 62.99 | 25.79 | 60.06 | 62.24 | 32.87 |
| CE1 | m.23610 | Lipase | Streptococcus gallolyticus UCN34 | Yes | 0.02 | 0.81 | 0.15 | 0.56 | 9.97 | 10.11 | 17.45 | 11.07 | 14.71 |
| CE1 | m.24395 | Uknown Alpha/Beta Hydrolase | Lachnospiraceae bacterium A4 | Yes | -0.08 | 0.31 | -0.68 | 0.59 | 66.12 | 62.71 | 82.13 | 41.38 | 99.78 |
| CE1 | m.27776 | Uknown | Oceanimonas smirnovii | No | -0.61 | 0.67 | -1.08 | -1.23 | 2.90 | 1.90 | 4.61 | 1.37 | 1.24 |
| CE1 | m.28175 | carboxymethylenebutenolidase | Methanobrevibacter ruminantium M1 | No | 0.85 | 1.44 | 0.89 | 1.08 | 1.01 | 1.82 | 2.74 | 1.87 | 2.12 |
| CE1 | m.5197 | Unknown | Shuttleworthia satelles | Yes | 0.23 | 0.59 | -0.25 | 0.29 | 0.84 | 0.99 | 1.27 | 0.71 | 1.03 |
|  |  |  |  |  |  |  |  |  |  |  |  |  |  |
| CE2 | m.15026 | AcetylXylan Esterase | Clostridium thermocellum | Yes | 5.97 | 5.10 | 2.98 | 3.37 | 0.47 | 29.27 | 16.02 | 3.67 | 4.81 |
| CE2 | m.15038 | AcetylXylan Esterase | Clostridium thermocellum | Yes | 2.23 | 1.92 | -2.61 | -0.93 | 4.92 | 23.05 | 18.62 | 0.80 | 2.58 |
| CE2 | m.15442 | AcetylXylan Esterase | Clostridium thermocellum | Yes | 0.44 | -3.57 | -4.04 | -1.86 | 1.94 | 2.63 | 0.16 | 0.12 | 0.53 |
| CE2 | m.20955 | AcetylXylan Esterase | Clostridium thermocellum | Yes | -3.63 | -0.09 | 2.71 | 1.33 | 13.32 | 1.08 | 12.53 | 87.34 | 33.56 |
|  |  |  |  |  |  |  |  |  |  |  |  |  |  |
| CE3 | m.20195 | AcetylXylan Esterase | Treponema primitia | Yes | 0.00 | 19.59 | 21.80 | 21.40 | 0.00 | 0.00 | 0.79 | 3.64 | 2.77 |
| CE3 | m.20202 | AcetylXylan Esterase | Treponema primitia | Yes | -4.89 | -3.78 | -3.62 | -3.32 | 6.96 | 0.23 | 0.51 | 0.57 | 0.70 |
| CE3 | m.20204 | AcetylXylan Esterase | Treponema primitia | Yes | -1.74 | -0.63 | 0.59 | 0.49 | 50.15 | 14.97 | 32.41 | 75.23 | 70.38 |
| CE3 | m.21935 | AcetylXylan Esterase | Clostridium thermocellum | Yes | 17.12 | 20.03 | 18.33 | 20.03 | 0.00 | 0.14 | 1.07 | 0.33 | 1.07 |
| CE3 | m.2599 | AcetylXylan Esterase | Neocallimastix patriciarum | No | 1.28 | 1.51 | 0.91 | 1.90 | 51.92 | 125.84 | 148.06 | 97.80 | 194.34 |
| CE3 | m.5004 | AcetylXylan Esterase | Clostridium papyrosolvens | Yes | 3.39 | 4.09 | 4.11 | 3.34 | 0.22 | 2.27 | 3.69 | 3.73 | 2.19 |
| CE3 | m.5464 | AcetylXylan Esterase | Clostridium papyrosolvens | Yes | -1.30 | -3.10 | -5.00 | -0.83 | 39.24 | 15.91 | 4.57 | 1.23 | 22.13 |
| CE3 | m.8522 | AcetylXylan Esterase | Rhodothermus marinus SG0.5JP17-172 | Yes | -1.10 | -3.33 | -4.30 | -1.52 | 56.24 | 26.22 | 5.59 | 2.85 | 19.68 |
| CE3 | m.8523 | AcetylXylan Esterase | Zunongwangia profunda SM-A87 | Yes | -1.57 | -5.16 | -6.44 | -3.22 | 17.22 | 5.79 | 0.48 | 0.20 | 1.85 |
|  |  |  |  |  |  |  |  |  |  |  |  |  |  |
| CE4 | m.10471 | Chitin DeAcetlyase | Pyronema omphalodes CBS 100304 | No | -2.39 | -1.82 | 0.14 | -0.66 | 22.85 | 4.36 | 6.49 | 25.24 | 14.42 |
| CE4 | m.12624 | Chitin DeAcetlyase | Rhizophagus irregularis DAOM 181602 | No | -2.49 | -1.47 | -1.30 | -1.73 | 3.55 | 0.63 | 1.28 | 1.44 | 1.07 |
| CE4 | m.13916 | Chitin DeAcetlyase | Rhizopus delemar RA 99-880 | No | -0.50 | -0.33 | 0.99 | 0.11 | 155.83 | 110.40 | 123.76 | 308.79 | 167.89 |
| CE4 | m.13917 | Uknown Most Likely FE | Rhizopus delemar RA 99-880 | No | -25.04 | -5.75 | -25.04 | -8.53 | 34.55 | 0.00 | 0.64 | 0.00 | 0.09 |
| CE4 | m.13920 | Chitin DeAcetlyase | Rhizopus delemar RA 99-880 | No | -2.09 | -1.27 | 0.81 | -0.06 | 219.85 | 51.61 | 91.00 | 385.35 | 210.37 |
| CE4 | m.14535 | Chitin DeAcetlyase | Rhizoctonia solani AG-3 Rhs1AP | No | -1.63 | -1.55 | -1.36 | -1.22 | 220.52 | 71.39 | 75.18 | 85.97 | 94.97 |
| CE4 | m.14536 | Chitin DeAcetlyase | Colletotrichum fioriniae PJ7 | No | -2.11 | -0.54 | 0.91 | 0.29 | 245.41 | 56.74 | 168.36 | 461.14 | 300.09 |
| CE4 | m.15505 | Uknown Conserved ExtraecellularSerine Rich protein | Cyphellophora europaea CBS 101466 | No | 0.31 | 0.81 | 2.39 | 1.63 | 0.78 | 0.96 | 1.36 | 4.07 | 2.40 |
| CE4 | m.16043 | Uknown Most Likely FE | Rhizopus delemar RA 99-880 | No | -1.36 | -1.81 | -0.39 | -1.19 | 627.67 | 244.00 | 178.54 | 479.47 | 275.03 |
| CE4 | m.16044 | Chitin DeAcetlyase | Mucor circinelloides f. circinelloides 1006PhL | No | 1.41 | 0.49 | 1.05 | 0.93 | 108.27 | 287.18 | 151.95 | 224.14 | 205.58 |
| CE4 | m.1623 | Uknown Most Likely FE | Rhizopus delemar RA 99-880 | No | 0.21 | -2.32 | -1.53 | -0.70 | 7.78 | 8.99 | 1.56 | 2.70 | 4.80 |
| CE4 | m.1737 | Chitin DeAcetlyase | Aspergillus oryzae RIB40 | No | -0.56 | -3.73 | -1.53 | -4.56 | 16.91 | 11.43 | 1.27 | 5.85 | 0.72 |
| CE4 | m.18227 | Chitin DeAcetlyase | Clostridium bolteae | Yes | -1.85 | -1.99 | -3.08 | -0.92 | 14.51 | 4.02 | 3.66 | 1.72 | 7.68 |
| CE4 | m.20876 | Polysaccraide DeAcetlyase | Fibrobacter succinogenes subsp. succinogenes S85 | Yes | -1.06 | -1.06 | -0.68 | 0.16 | 566.49 | 271.46 | 270.94 | 352.98 | 631.50 |
| CE4 | m.20908 | Polysaccraide DeAcetlyase | Fibrobacter succinogenes subsp. succinogenes S85 | Yes | -2.90 | -2.88 | -2.82 | -1.70 | 3.58 | 0.48 | 0.49 | 0.51 | 1.10 |
| CE4 | m.22451 | Chitin DeAcetlyase | Rhizopus delemar RA 99-880 | No | 1.06 | 3.13 | 3.72 | 3.58 | 1.96 | 4.08 | 17.15 | 25.85 | 23.49 |
| CE4 | m.22456 | Chitin DeAcetlyase | Rhizopus delemar RA 99-880 | No | 2.40 | 3.48 | 0.99 | 0.74 | 0.78 | 4.12 | 8.70 | 1.55 | 1.30 |
| CE4 | m.22463 | Chitin DeAcetlyase | Fusarium verticillioides 7600 | No | 0.65 | 0.80 | 0.99 | 1.01 | 15.40 | 24.11 | 26.73 | 30.62 | 30.99 |
| CE4 | m.22464 | Chitin DeAcetlyase | Rhizopus delemar RA 99-880 | No | -0.07 | 0.57 | -0.98 | -2.16 | 6.18 | 5.90 | 9.14 | 3.13 | 1.39 |
| CE4 | m.22563 | Polysaccraide DeAcetlyase | Rhizophagus irregularis DAOM 197198w | No | -1.77 | 2.10 | 1.68 | 4.27 | 8.70 | 2.54 | 37.23 | 27.82 | 168.26 |
| CE4 | m.22564 | Chitin DeAcetlyase | Tuber melanosporum Mel28 | No | 1.55 | 0.03 | -0.55 | 2.25 | 9.09 | 26.68 | 9.28 | 6.23 | 43.14 |
| CE4 | m.22565 | Chitin DeAcetlyase | Rhizophagus irregularis DAOM 197198w | No | 4.13 | 1.75 | 3.04 | 3.68 | 0.94 | 16.46 | 3.17 | 7.76 | 12.02 |
| CE4 | m.22566 | Chitin DeAcetlyase | Rhizophagus irregularis DAOM 197198w | No | 2.43 | -2.45 | 0.31 | 0.34 | 1.73 | 9.33 | 0.32 | 2.14 | 2.19 |
| CE4 | m.22567 | Chitin DeAcetlyase | Tuber melanosporum Mel28 | No | 0.77 | -1.40 | 0.99 | -0.87 | 20.18 | 34.48 | 7.63 | 40.18 | 11.04 |
| CE4 | m.22569 | Chitin DeAcetlyase | Rhizophagus irregularis DAOM 197198w | No | 22.27 | 21.46 | 17.06 | 0.00 | 0.00 | 5.05 | 2.89 | 0.14 | 0.00 |
| CE4 | m.22570 | Chitin DeAcetlyase | Tuber melanosporum Mel28 | No | 3.87 | 2.25 | 2.88 | 3.22 | 2.69 | 39.43 | 12.81 | 19.77 | 25.05 |
| CE4 | m.23021 | Chitin DeAcetlyase | Gaeumannomyces graminis var. tritici R3-111a-1 | No | -2.74 | -1.50 | -0.99 | -1.58 | 52.76 | 7.90 | 18.61 | 26.61 | 17.69 |
| CE4 | m.23023 | Chitin DeAcetlyase | Gaeumannomyces graminis var. tritici R3-111a-1 | No | -4.59 | -2.69 | -0.68 | -0.03 | 83.57 | 3.48 | 12.92 | 52.27 | 81.72 |
| CE4 | m.23025 | Chitin DeAcetlyase | Mucor circinelloides f. circinelloides 1006PhL | No | -1.04 | 0.29 | 1.48 | 1.60 | 19.32 | 9.39 | 23.58 | 54.05 | 58.59 |
| CE4 | m.23030 | Chitin DeAcetlyase | Gaeumannomyces graminis var. tritici R3-111a-1 | No | -3.46 | 0.03 | -0.29 | -2.79 | 86.30 | 7.84 | 88.40 | 70.68 | 12.44 |
| CE4 | m.24165 | Chitin DeAcetlyase | Rhizopus delemar RA 99-880 | No | -1.83 | -0.85 | 0.07 | -1.59 | 643.09 | 181.22 | 357.58 | 676.85 | 213.77 |
| CE4 | m.24342 | Chitin DeAcetlyase | Rhizophagus irregularis DAOM 197198w | No | 1.91 | 2.94 | 1.96 | 1.52 | 52.58 | 197.42 | 402.33 | 205.07 | 150.60 |
| CE4 | m.25494 | Chitin DeAcetlyase | Rhizophagus irregularis DAOM 197198w | No | -1.41 | 0.57 | 2.02 | 1.07 | 16.34 | 6.13 | 24.23 | 66.15 | 34.31 |
| CE4 | m.25621 | Chitin DeAcetlyase | Mucor circinelloides f. circinelloides 1006PhL | No | 3.96 | 4.01 | 3.48 | 3.11 | 1.28 | 19.82 | 20.64 | 14.25 | 11.01 |
| CE4 | m.25791 | Chitin DeAcetlyase | Mucor circinelloides f. circinelloides 1006PhL | No | -1.27 | 1.73 | 2.59 | 2.80 | 1.13 | 0.47 | 3.72 | 6.78 | 7.83 |
| CE4 | m.25991 | Chitin DeAcetlyase | Trachipleistophora hominis | No | 1.85 | 1.61 | 1.40 | 1.28 | 3.28 | 11.83 | 10.06 | 8.69 | 8.00 |
| CE4 | m.26144 | Uknown Most Likely FE | Moniliophthora roreri MCA 2997 | No | 4.28 | 4.80 | 3.66 | 3.84 | 0.41 | 7.89 | 11.30 | 5.13 | 5.81 |
| CE4 | m.26365 | Chitin DeAcetlyase | Rhizoctonia solani AG-3 Rhs1AP | No | 1.82 | 1.98 | 1.36 | 1.27 | 2.98 | 10.52 | 11.78 | 7.67 | 7.21 |
| CE4 | m.27257 | Chitin DeAcetlyase | Rhizopus delemar RA 99-880 | No | -2.61 | -1.70 | -1.25 | -3.04 | 10.90 | 1.79 | 3.35 | 4.57 | 1.33 |
| CE4 | m.27825 | Chitin DeAcetlyase | Coprinopsis cinerea okayama7#130 | No | 3.54 | -0.62 | -0.90 | 0.09 | 0.71 | 8.27 | 0.46 | 0.38 | 0.76 |
| CE4 | m.2856 | Polysaccraide DeAcetlyase | Rhizopus delemar RA 99-880 | No | -1.90 | -1.81 | 0.07 | -1.29 | 569.25 | 152.21 | 162.59 | 596.92 | 233.57 |
| CE4 | m.4169 | Polysaccraide DeAcetlyase | Alkaliphilus metalliredigens QYMF | Yes | 2.30 | -18.29 | -18.29 | -18.29 | 0.32 | 1.57 | 0.00 | 0.00 | 0.00 |
| CE4 | m.4713 | Chitin DeAcetlyase | Batrachochytrium dendrobatidis JAM81 | No | 4.16 | 3.00 | 1.81 | 2.26 | 5.17 | 92.30 | 41.42 | 18.12 | 24.71 |
| CE4 | m.5487 | Chitin DeAcetlyase | Rhizopus delemar RA 99-880 | No | 3.89 | 2.85 | 2.47 | 2.50 | 1.94 | 28.71 | 14.01 | 10.75 | 11.00 |
| CE4 | m.5665 | Chitin DeAcetlyase | Rhizopus delemar RA 99-880 | No | 3.72 | 3.21 | 3.90 | 3.07 | 0.46 | 6.03 | 4.25 | 6.83 | 3.85 |
| CE4 | m.5882 | Chitin DeAcetlyase | Moniliophthora roreri MCA 2997 | No | -1.73 | -1.61 | 2.09 | 0.83 | 1.61 | 0.49 | 0.53 | 6.86 | 2.87 |
| CE4 | m.5883 | Chitin DeAcetlyase | Batrachochytrium dendrobatidis JAM81 | No | -2.26 | -0.94 | 2.73 | 1.22 | 1.84 | 0.38 | 0.96 | 12.23 | 4.29 |
| CE4 | m.5884 | Chitin DeAcetlyase | Dacryopinax sp. DJM-731 SS1 | No | -2.72 | -0.31 | 2.86 | 1.37 | 0.64 | 0.10 | 0.52 | 4.65 | 1.65 |
| CE4 | m.5885 | Chitin DeAcetlyase | Moniliophthora roreri MCA 2997 | No | -20.83 | -2.16 | 2.09 | 0.43 | 1.86 | 0.00 | 0.42 | 7.90 | 2.50 |
| CE4 | m.6185 | Chitin DeAcetlyase | Rhizophagus irregularis DAOM 197198w | No | 3.58 | 2.12 | 0.36 | 0.57 | 3.36 | 40.11 | 14.57 | 4.31 | 4.97 |
| CE4 | m.6270 | Chitin DeAcetlyase | Fusarium graminearum PH-1 | No | -0.98 | -0.34 | 1.21 | 1.01 | 4.89 | 2.48 | 3.86 | 11.30 | 9.83 |
| CE4 | m.8549 | Chitin DeAcetlyase | Enterococcus faecium EnGen0263 | Yes | 2.42 | -0.94 | 1.92 | 1.57 | 0.24 | 1.30 | 0.13 | 0.92 | 0.72 |
| CE4 | m.11041 | Chitin DeAcetlyase | Mucor circinelloides f. circinelloides 1006PhL | No | -0.96 | 0.48 | 0.44 | -0.22 | 2.62 | 1.34 | 3.66 | 3.56 | 2.25 |
| CE4 | m.12625 | Polysaccraide DeAcetlyase | Tuber melanosporum Mel28 | No | -0.24 | -0.88 | 0.96 | -0.28 | 2.06 | 1.75 | 1.12 | 4.01 | 1.70 |
| CE4 | m.16042 | Uknown Most Likely FE | Rhizopus delemar RA 99-880 | No | 0.00 | 0.00 | 17.06 | 0.00 | 0.00 | 0.00 | 0.00 | 0.14 | 0.00 |
| CE4 | m.17134 | Polysaccraide DeAcetlyase | Mucor circinelloides f. circinelloides 1006PhL | No | -0.65 | -0.07 | -0.76 | 0.36 | 66.14 | 42.15 | 63.20 | 39.08 | 84.90 |
| CE4 | m.22453 | Chitin DeAcetlyase | Rhizopus delemar RA 99-880 | No | 1.54 | 0.43 | 0.64 | 0.00 | 3.83 | 11.12 | 5.15 | 5.94 | 3.83 |
| CE4 | m.22454 | Chitin DeAcetlyase | Rhizopus delemar RA 99-880 | No | 1.00 | 0.84 | -0.92 | -2.72 | 0.88 | 1.76 | 1.58 | 0.47 | 0.13 |
| CE4 | m.22459 | Chitin DeAcetlyase | Rhizopus delemar RA 99-880 | No | 2.46 | 1.01 | -0.78 | -2.06 | 3.20 | 17.60 | 6.44 | 1.86 | 0.77 |
| CE4 | m.24158 | Chitin DeAcetlyase | Rhizopus delemar RA 99-880 | No | -0.75 | -0.75 | -0.52 | -0.76 | 1918.75 | 1143.23 | 1137.97 | 1336.77 | 1131.50 |
| CE4 | m.24511 | Chitin DeAcetlyase | Mucor circinelloides f. circinelloides 1006PhL | No | -0.44 | -0.12 | 0.94 | -0.21 | 85.83 | 63.07 | 78.93 | 164.95 | 74.32 |
| CE4 | m.25972 | Chitin DeAcetlyase | Mucor circinelloides f. circinelloides 1006PhL | No | 0.62 | -0.35 | -0.11 | -0.71 | 8.91 | 13.72 | 6.97 | 8.23 | 5.45 |
| CE4 | m.26382 | Chitin DeAcetlyase | Magnaporthe oryzae Y34 | No | 0.11 | -0.78 | -0.11 | 0.36 | 5.81 | 6.30 | 3.38 | 5.40 | 7.45 |
| CE4 | m.8227 | Chitin DeAcetlyase | Agaricus bisporus var. burnettii JB137-S8 | No | -0.42 | 1.71 | 0.35 | -1.44 | 0.56 | 0.41 | 1.82 | 0.71 | 0.21 |
| CE4 | m.8673 | Uknown Most Likely FE | Mucor circinelloides f. circinelloides 1006PhL | No | 1.72 | 0.89 | 1.38 | -0.95 | 0.54 | 1.77 | 0.99 | 1.41 | 0.28 |
|  |  |  |  |  |  |  |  |  |  |  |  |  |  |
| CE6 | m.14828 | AcetylXylan Esterase | Haemophilus somnus | Yes | -1.28 | -1.62 | -0.76 | 0.24 | 53.65 | 22.02 | 17.45 | 31.77 | 63.45 |
| CE6 | m.14831 | AcetylXylan Esterase | Piromyces sp. | No | 1.41 | 0.16 | 1.32 | 1.05 | 8.44 | 22.36 | 9.44 | 21.11 | 17.47 |
| CE6 | m.26027 | AcetylXylan Esterase | Arabidopsis thaliana | No | -1.65 | -2.41 | -1.07 | -0.07 | 36.37 | 11.61 | 6.83 | 17.33 | 34.68 |
| CE6 | m.5607 | AcetylXylan Esterase | Mus musculus | No | 2.82 | 2.46 | 2.36 | 1.52 | 0.28 | 2.01 | 1.57 | 1.46 | 0.81 |
| CE6 | m.6313 | AcetylXylan Esterase | Arabidopsis thaliana | No | -3.43 | -4.50 | -4.60 | -2.43 | 248.78 | 23.07 | 11.03 | 10.27 | 46.10 |
| CE6 | m.6314 | AcetylXylan Esterase | Saccharomyces cerevisiae | No | -3.25 | -4.79 | -5.12 | -2.75 | 632.51 | 66.37 | 22.86 | 18.17 | 94.12 |
| CE6 | m.9589 | AcetylXylan Esterase | Bordetella parapertussis | Yes | -1.15 | 0.88 | 1.26 | 0.80 | 18.46 | 8.35 | 33.97 | 44.24 | 32.13 |
| CE6 | m.9590 | AcetylXylan Esterase | Mus musculus | No | -1.19 | -2.06 | -3.58 | -0.30 | 40.46 | 17.74 | 9.67 | 3.38 | 32.95 |
| CE6 | m.9591 | AcetylXylan Esterase | Drosophila melanogaster | No | -1.32 | -2.32 | -3.38 | -0.38 | 63.23 | 25.37 | 12.70 | 6.06 | 48.54 |
| CE6 | m.27611 | AcetylXylan Esterase | Chloroflexus aurantiacus | Yes | 0.18 | -0.50 | 1.32 | 1.03 | 0.79 | 0.90 | 0.56 | 1.98 | 1.62 |
|  |  |  |  |  |  |  |  |  |  |  |  |  |  |
| CE7 | m.24243 | AcetylXylan Esterase | Ruminococcus sp. CAG:353 | Yes | -1.32 | -0.94 | -0.39 | -0.48 | 436.66 | 175.08 | 228.37 | 333.78 | 313.09 |
| CE7 | m.27221 | AcetylXylan Esterase | Ruminococcus flavefaciens | Yes | 0.98 | -0.52 | -3.12 | -0.86 | 3.65 | 7.19 | 2.54 | 0.42 | 2.01 |
| CE7 | m.28005 | AcetylXylan Esterase | Butyrivibrio fibrisolvens | Yes | 1.14 | 0.39 | -2.09 | -1.76 | 1.54 | 3.40 | 2.01 | 0.36 | 0.45 |
| CE7 | m.24585 | AcetylXylan Esterase | unclassified Erysipelotrichaceae | Yes | -0.68 | -0.01 | 0.56 | -0.03 | 47.80 | 29.92 | 47.57 | 70.46 | 46.94 |
|  |  |  |  |  |  |  |  |  |  |  |  |  |  |
| CE8 | m.14358 | pectin methylesterase | Arthrobotrys oligospora ATCC 24927 | No | 0.42 | 1.25 | 0.98 | 0.58 | 21.11 | 28.20 | 50.17 | 41.69 | 31.51 |
| CE8 | m.7074 | pectin methylesterase | Neofusicoccum parvum UCRNP2 | No | 3.86 | 3.01 | 0.86 | 1.32 | 0.24 | 3.49 | 1.94 | 0.44 | 0.60 |
| CE8 | m.9597 | pectin methylesterase | Oryza sativa Japonica Group | No | 20.65 | 19.99 | 18.48 | 18.06 | 0.00 | 1.64 | 1.04 | 0.37 | 0.27 |
| CE8 | m.14361 | pectin methylesterase | Paenibacillus massiliensis | Yes | 0.54 | 0.69 | 1.32 | 1.17 | 4.15 | 6.04 | 6.68 | 10.34 | 9.35 |
| CE8 | m.14362 | pectin methylesterase | Drechslerella stenobrocha 248 | No | 0.85 | 0.19 | 0.46 | -0.28 | 3.95 | 7.14 | 4.49 | 5.45 | 3.25 |
|  |  |  |  |  |  |  |  |  |  |  |  |  |  |
| CE9 | m.2036 | N-acetylglucosamine 6-phosphate deacetylase | Oscillibacter sp. CAG:241 | Yes | 2.40 | 1.68 | 0.87 | 0.72 | 0.82 | 4.33 | 2.63 | 1.50 | 1.35 |
|  |  |  |  |  |  |  |  |  |  |  |  |  |  |
| CE10 | m.10117 | Non Carbohydrate Substrate | Phytophthora parasitica P1569 | No | 2.17 | 0.22 | 1.86 | 0.99 | 0.54 | 2.42 | 0.63 | 1.96 | 1.07 |
| CE10 | m.10118 | Non Carbohydrate Substrate | Aphanomyces invadans | No | 1.13 | -0.24 | -1.23 | 0.31 | 4.09 | 8.95 | 3.48 | 1.74 | 5.06 |
| CE10 | m.11209 | Non Carbohydrate Substrate | Mucor circinelloides f. circinelloides 1006PhL | No | 0.77 | 1.55 | 1.00 | -0.04 | 5.45 | 9.25 | 15.96 | 10.92 | 5.28 |
| CE10 | m.11210 | Non Carbohydrate Substrate | Caulobacter crescentus CB15 | No | 0.34 | 1.59 | 1.96 | 1.18 | 3.08 | 3.91 | 9.30 | 11.98 | 6.99 |
| CE10 | m.15114 | Non Carbohydrate Substrate | Physcomitrella patens | No | 2.20 | 1.59 | 0.64 | 0.93 | 2.28 | 10.51 | 6.88 | 3.56 | 4.33 |
| CE10 | m.15115 | Non Carbohydrate Substrate | Ahrensia kielensis | No | 22.01 | 20.31 | 18.09 | 14.84 | 0.00 | 4.23 | 1.30 | 0.28 | 0.03 |
| CE10 | m.15117 | Non Carbohydrate Substrate | Croceibacter atlanticus HTCC2559 | No | 2.71 | 3.31 | 1.80 | 2.15 | 0.67 | 4.40 | 6.64 | 2.33 | 2.97 |
| CE10 | m.15119 | Non Carbohydrate Substrate | Saprolegnia diclina VS20 | No | 2.70 | 2.21 | 1.73 | 1.57 | 0.54 | 3.51 | 2.48 | 1.78 | 1.60 |
| CE10 | m.15120 | Non Carbohydrate Substrate | Ahrensia kielensis | Yes | 2.14 | -0.48 | -19.61 | -3.52 | 0.80 | 3.52 | 0.57 | 0.00 | 0.07 |
| CE10 | m.15126 | Non Carbohydrate Substrate | Ahrensia kielensis | Yes | 4.08 | 0.48 | -0.08 | 0.50 | 0.25 | 4.23 | 0.35 | 0.24 | 0.35 |
| CE10 | m.15127 | Non Carbohydrate Substrate | Arabidopsis thaliana | No | 20.96 | 20.96 | 20.75 | 20.45 | 0.00 | 2.03 | 2.03 | 1.76 | 1.44 |
| CE10 | m.15128 | Non Carbohydrate Substrate | Batrachochytrium dendrobatidis JAM81 | No | 2.25 | -1.11 | -19.12 | -1.13 | 0.57 | 2.72 | 0.26 | 0.00 | 0.26 |
| CE10 | m.17084 | Non Carbohydrate Substrate | Aphanomyces invadans | No | -0.32 | -1.98 | -3.56 | -2.33 | 95.01 | 75.92 | 24.12 | 8.06 | 18.93 |
| CE10 | m.1795 | Non Carbohydrate Substrate | Phytophthora parasitica P10297 | No | 6.28 | 5.52 | 2.22 | 2.87 | 0.22 | 16.88 | 9.92 | 1.01 | 1.58 |
| CE10 | m.1796 | Non Carbohydrate Substrate | Lactobacillus rhamnosus MTCC 5462 | No | 20.70 | 20.76 | 16.51 | 19.56 | 0.00 | 1.71 | 1.77 | 0.09 | 0.77 |
| CE10 | m.19813 | Non Carbohydrate Substrate | Rhizophagus irregularis DAOM 181602 | No | -22.12 | -22.12 | -22.12 | -5.51 | 4.56 | 0.00 | 0.00 | 0.00 | 0.10 |
| CE10 | m.19815 | Non Carbohydrate Substrate | Rhizophagus irregularis DAOM 181602 | No | -3.09 | -1.13 | -1.42 | -0.80 | 1.47 | 0.17 | 0.67 | 0.55 | 0.85 |
| CE10 | m.21332 | Non Carbohydrate Substrate | Batrachochytrium dendrobatidis JAM81 | No | 3.46 | 0.98 | 1.97 | -1.34 | 0.24 | 2.67 | 0.48 | 0.95 | 0.10 |
| CE10 | m.2279 | Non Carbohydrate Substrate | Branchiostoma floridae | No | 6.01 | 4.47 | 0.65 | 0.95 | 0.24 | 15.66 | 5.40 | 0.38 | 0.47 |
| CE10 | m.2280 | Non Carbohydrate Substrate | Aphanomyces astaci | No | 21.26 | 20.96 | 18.38 | 17.84 | 0.00 | 2.51 | 2.04 | 0.34 | 0.23 |
| CE10 | m.2432 | Non Carbohydrate Substrate | Peptoniphilus sp. BV3AC2 | Yes | -3.17 | -2.22 | -1.27 | -0.69 | 10.99 | 1.22 | 2.36 | 4.54 | 6.82 |
| CE10 | m.24560 | Non Carbohydrate Substrate | Clostridium acetobutylicum ATCC 824 | Yes | -3.24 | 0.27 | -0.27 | -2.34 | 142.75 | 15.11 | 172.10 | 118.77 | 28.21 |
| CE10 | m.24674 | Non Carbohydrate Substrate | Batrachochytrium dendrobatidis JAM81 | No | -0.78 | -1.08 | -2.14 | -2.09 | 37.05 | 21.58 | 17.47 | 8.39 | 8.73 |
| CE10 | m.24941 | Non Carbohydrate Substrate | Peptoniphilus duerdenii | Yes | -2.15 | -1.87 | 0.39 | -0.61 | 42.10 | 9.49 | 11.52 | 55.13 | 27.65 |
| CE10 | m.24991 | Non Carbohydrate Substrate | Batrachochytrium dendrobatidis JAM81 | No | 0.80 | 1.09 | 2.41 | 1.85 | 15.00 | 26.08 | 31.84 | 79.62 | 54.22 |
| CE10 | m.25053 | Non Carbohydrate Substrate | Firmicutes bacterium CAG:194 | Yes | -4.51 | -0.88 | -2.56 | -0.80 | 51.21 | 2.25 | 27.92 | 8.67 | 29.41 |
| CE10 | m.25214 | Non Carbohydrate Substrate | Mucor circinelloides f. circinelloides 1006PhL | No | -0.49 | -1.98 | -5.68 | -1.49 | 54.14 | 38.53 | 13.69 | 1.06 | 19.22 |
| CE10 | m.25496 | Non Carbohydrate Substrate | Batrachochytrium dendrobatidis JAM81 | No | -1.09 | -0.73 | -1.32 | -1.88 | 24.28 | 11.42 | 14.64 | 9.70 | 6.62 |
| CE10 | m.25594 | Non Carbohydrate Substrate | Saprolegnia diclina VS20 | No | 5.44 | 5.17 | 1.96 | 2.62 | 0.84 | 36.67 | 30.39 | 3.29 | 5.20 |
| CE10 | m.25722 | Non Carbohydrate Substrate | Butyrivibrio fibrisolvens 16/4 | Yes | 4.76 | 3.83 | 2.72 | 2.60 | 1.54 | 41.72 | 21.83 | 10.12 | 9.30 |
| CE10 | m.25809 | Non Carbohydrate Substrate | Ahrensia kielensis | No | 2.06 | 1.21 | -0.06 | 0.08 | 9.07 | 37.83 | 20.98 | 8.70 | 9.56 |
| CE10 | m.25968 | Non Carbohydrate Substrate | Clostridium sp. CAG:230 | No | -1.58 | -1.08 | 0.57 | -0.30 | 18.45 | 6.17 | 8.70 | 27.35 | 14.95 |
| CE10 | m.26535 | Non Carbohydrate Substrate | Piriformospora indica DSM 11827 | Yes | -3.92 | -1.45 | -1.70 | -2.38 | 14.45 | 0.96 | 5.31 | 4.45 | 2.77 |
| CE10 | m.26650 | Non Carbohydrate Substrate | Batrachochytrium dendrobatidis JAM81 | No | -0.36 | 1.90 | 0.67 | -0.95 | 3.13 | 2.44 | 11.64 | 4.96 | 1.61 |
| CE10 | m.26932 | Non Carbohydrate Substrate | Aphanomyces invadans | No | -0.60 | -0.74 | -0.73 | -1.59 | 8.78 | 5.78 | 5.25 | 5.30 | 2.92 |
| CE10 | m.28321 | Non Carbohydrate Substrate | Aphanomyces invadans | No | 21.04 | 19.85 | 16.92 | 17.69 | 0.00 | 2.16 | 0.94 | 0.12 | 0.21 |
| CE10 | m.3259 | Non Carbohydrate Substrate | Roseburia sp. CAG:45 | Yes | -3.74 | -4.92 | -4.59 | -4.36 | 4.25 | 0.32 | 0.14 | 0.18 | 0.21 |
| CE10 | m.3311 | Non Carbohydrate Substrate | Phytophthora parasitica P1569 | No | 1.68 | -0.17 | -0.44 | -0.26 | 10.62 | 33.93 | 9.46 | 7.85 | 8.88 |
| CE10 | m.4649 | Non Carbohydrate Substrate | Roseburia sp. CAG:45 | Yes | 3.45 | 2.76 | 0.40 | 0.83 | 0.20 | 2.16 | 1.33 | 0.26 | 0.35 |
| CE10 | m.5347 | Non Carbohydrate Substrate | Batrachochytrium dendrobatidis JAM81 | No | -1.56 | -1.93 | -2.80 | -4.71 | 5.94 | 2.01 | 1.55 | 0.85 | 0.23 |
| CE10 | m.5936 | Non Carbohydrate Substrate | Rhizophagus irregularis DAOM 197198w | No | -0.01 | 1.16 | 1.76 | 0.53 | 4.52 | 4.49 | 10.15 | 15.29 | 6.53 |
| CE10 | m.6045 | Non Carbohydrate Substrate | Nocardiopsis ganjiahuensis | Yes | -2.52 | -1.44 | 0.23 | 0.01 | 1.11 | 0.19 | 0.41 | 1.30 | 1.11 |
| CE10 | m.7216 | Non Carbohydrate Substrate | Ogataea parapolymorpha DL-1 | No | -5.56 | -5.23 | -21.65 | -21.65 | 3.29 | 0.07 | 0.09 | 0.00 | 0.00 |
| CE10 | m.7217 | Non Carbohydrate Substrate | Aphanomyces astaci | No | -4.09 | -21.76 | -21.76 | -21.76 | 3.55 | 0.21 | 0.00 | 0.00 | 0.00 |
| CE10 | m.8216 | Non Carbohydrate Substrate | Rhizophagus irregularis DAOM 181602 | No | -2.17 | -3.13 | -4.94 | -5.53 | 2.16 | 0.48 | 0.25 | 0.07 | 0.05 |
| CE10 | m.8374 | Non Carbohydrate Substrate | Batrachochytrium dendrobatidis JAM81 | No | 4.28 | 3.88 | 3.81 | 2.51 | 0.14 | 2.78 | 2.09 | 2.00 | 0.81 |
| CE10 | m.9631 | Non Carbohydrate Substrate | Clostridium hathewayi | Yes | -3.83 | -1.87 | -3.34 | -5.26 | 3.73 | 0.26 | 1.02 | 0.37 | 0.10 |
| CE10 | m.9772 | Non Carbohydrate Substrate | Aphanomyces invadans | No | 4.77 | 3.82 | 4.39 | 3.64 | 0.06 | 1.70 | 0.88 | 1.31 | 0.78 |
| CE10 | m.1020 | Non Carbohydrate Substrate | Batrachochytrium dendrobatidis JAM81 | Yes | -0.74 | 0.08 | -0.06 | -0.51 | 23.54 | 14.06 | 24.84 | 22.60 | 16.52 |
| CE10 | m.13822 | Non Carbohydrate Substrate | Batrachochytrium dendrobatidis JAM81 | No | 2.69 | 0.48 | -2.26 | -0.91 | 0.35 | 2.28 | 0.49 | 0.07 | 0.19 |
| CE10 | m.26038 | Non Carbohydrate Substrate | Butyrivibrio proteoclasticus B316 | Yes | 1.14 | 1.15 | -0.69 | -0.73 | 6.63 | 14.60 | 14.73 | 4.12 | 4.00 |
| CE10 | m.27151 | Non Carbohydrate Substrate | Aphanomyces astaci | No | 1.27 | 0.90 | 0.57 | 0.53 | 4.28 | 10.29 | 8.01 | 6.34 | 6.16 |
| CE10 | m.27223 | Non Carbohydrate Substrate | Roseburia sp. CAG:182 | Yes | -2.27 | -2.50 | -2.45 | -2.28 | 11.79 | 2.44 | 2.09 | 2.16 | 2.42 |
| CE10 | m.28431 | Non Carbohydrate Substrate | Verrucomicrobia bacterium SCGC AAA164-E04 | Yes | 2.97 | 1.82 | -0.81 | -1.65 | 0.19 | 1.48 | 0.66 | 0.11 | 0.06 |
| CE10 | m.2945 | Non Carbohydrate Substrate | Clostridium acetobutylicum ATCC 824 | Yes | 0.21 | -0.42 | 0.43 | 0.22 | 0.89 | 1.02 | 0.66 | 1.19 | 1.03 |
| CE10 | m.3309 | Non Carbohydrate Substrate | Aphanomyces astaci | No | 1.31 | 0.62 | 0.05 | 0.13 | 4.24 | 10.53 | 6.52 | 4.37 | 4.63 |
| CE10 | m.4710 | Non Carbohydrate Substrate | Sphingobacterium sp. 21 | Yes | 1.79 | 0.89 | -0.88 | -0.48 | 0.46 | 1.58 | 0.85 | 0.25 | 0.33 |
| CE10 | m.6334 | Non Carbohydrate Substrate | Rhizophagus irregularis DAOM 181602 | Viral | 0.29 | 1.97 | 1.40 | 1.95 | 0.46 | 0.56 | 1.79 | 1.21 | 1.76 |
|  |  |  |  |  |  |  |  |  |  |  |  |  |  |
| CE12 | m.26867 | rhamnogalacturonan acetylesterase | Erinaceus europaeus | No | -1.64 | -0.57 | -0.02 | -0.94 | 6.56 | 2.10 | 4.43 | 6.46 | 3.43 |
| CE12 | m.10844 | rhamnogalacturonan acetylesterase | Leucoagaricus gongylophorus | No | 1.06 | -0.33 | 0.03 | -0.46 | 13.52 | 28.24 | 10.78 | 13.81 | 9.82 |
| CE12 | m.24513 | rhamnogalacturonan acetylesterase | Coprinopsis cinerea okayama7#130 | No | -0.46 | -0.67 | 0.70 | 0.55 | 89.29 | 64.88 | 56.23 | 144.93 | 130.81 |
| CE12 | m.24986 | rhamnogalacturonan acetylesterase | Candidatus Solibacter usitatus Ellin6076 | No | -0.31 | -0.42 | 0.25 | -0.42 | 45.15 | 36.49 | 33.73 | 53.69 | 33.78 |
|  |  |  |  |  |  |  |  |  |  |  |  |  |  |
| CE14 | m.27680 | Non CAZy Gene | Branchiostoma floridae | No | 0.38 | 1.53 | 1.12 | 1.01 | 1.33 | 1.73 | 3.84 | 2.90 | 2.67 |
|  |  |  |  |  |  |  |  |  |  |  |  |  |  |
| CE15 | m.19659 | 4-O-methyl-glucuronoyl methylesterase | Cytophaga hutchinsonii ATCC 33406 | Yes | -0.41 | -1.91 | -2.74 | -0.33 | 303.32 | 228.79 | 80.47 | 45.52 | 241.89 |
| CE15 | m.8475 | 4-O-methyl-glucuronoyl methylesterase | Cytophaga hutchinsonii ATCC 33406 | Yes | -0.20 | -1.73 | -1.92 | -0.14 | 1.55 | 1.35 | 0.47 | 0.41 | 1.40 |
|  |  |  |  |  |  |  |  |  |  |  |  |  |  |
| CE16 | m.1118 | AcetylXylan Esterase | Botryotinia fuckeliana T4 | No | 0.08 | -1.21 | -0.87 | -0.18 | 8.04 | 8.47 | 3.48 | 4.41 | 7.08 |
| CE16 | m.19660 | AcetylXylan Esterase | Heterobasidion irregulare TC 32-1 | No | 1.16 | -0.05 | -0.98 | 1.90 | 15.72 | 35.02 | 15.19 | 7.99 | 58.65 |
| CE16 | m.19661 | GDSL hydroalse cellulose binding | Clostridium thermocellum | Yes | 0.00 | 0.00 | 16.19 | 23.51 | 0.00 | 0.00 | 0.00 | 0.07 | 11.91 |
| CE16 | m.19662 | AcetylXylan Esterase | Heterobasidion irregulare TC 32-1 | No | 0.40 | -1.76 | -2.96 | -0.09 | 60.88 | 80.57 | 18.01 | 7.80 | 57.28 |
| CE16 | m.25740 | GDSL hydroalse cellulose binding | Rhizoctonia solani AG-3 Rhs1AP | No | -2.61 | -0.65 | -1.07 | -1.64 | 22.23 | 3.65 | 14.17 | 10.58 | 7.15 |
| CE16 | m.25801 | GDSL hydroalse cellulose binding | Heterobasidion irregulare TC 32-1 | No | 1.58 | 2.36 | 1.40 | 0.65 | 3.91 | 11.71 | 20.05 | 10.36 | 6.15 |
| CE16 | m.27674 | GDSL hydroalse cellulose binding | Glarea lozoyensis ATCC 20868 | Yes | -4.84 | -2.27 | -3.56 | -3.33 | 14.11 | 0.49 | 2.92 | 1.19 | 1.40 |
| CE16 | m.27852 | GDSL hydroalse cellulose binding | Pseudocercospora fijiensis CIRAD86 | No | -0.54 | -0.75 | -2.84 | -1.81 | 4.58 | 3.15 | 2.73 | 0.64 | 1.30 |
| CE16 | m.4390 | GDSL hydroalse cellulose binding | Fomitopsis pinicola FP-58527 SS1 | Yes | -4.86 | -2.74 | -2.96 | -2.95 | 5.37 | 0.18 | 0.80 | 0.69 | 0.69 |
| CE16 | m.1120 | AcetylXylan Esterase | Dactylellina haptotyla CBS 200.50 | No | 0.83 | -0.22 | -0.06 | -0.13 | 2.75 | 4.87 | 2.37 | 2.65 | 2.51 |
| CE16 | m.13012 | GDSL hydroalse cellulose binding | Arthrobotrys oligospora ATCC 24927 | Yes | -0.31 | 0.96 | -0.09 | -0.36 | 3.49 | 2.82 | 6.81 | 3.28 | 2.72 |
| CE16 | m.13324 | GDSL hydroalse cellulose binding | Aspergillus terreus NIH2624 | No | -0.29 | 0.83 | 0.27 | -0.85 | 2.30 | 1.88 | 4.09 | 2.77 | 1.27 |
|  |  |  |  |  |  |  |  |  |  |  |  |  |  |
| CEX | m.16727 | Feruoyl Esterase | Piromyces equi | No | 18.40 | 19.02 | 20.99 | 23.57 | 0.00 | 0.35 | 0.53 | 2.09 | 12.46 |
| CEX | m.17507 | Carbhydrate Estrase | Ruminococcus flavefaciens | Yes | 1.58 | 0.70 | 0.71 | 0.92 | 6.30 | 18.88 | 10.25 | 10.34 | 11.91 |
| CEX | m.20885 | Acetylxylan Esterase | Neocallimastix patriciarum | No | -1.55 | -1.70 | -0.99 | -0.42 | 1016.00 | 347.88 | 313.69 | 511.15 | 761.30 |
| CEX | m.27981 | Acetylxylan Esterase | Neocallimastix patriciarum | No | 0.78 | -2.05 | -3.86 | -2.17 | 2.80 | 4.82 | 0.68 | 0.19 | 0.62 |
| CEX | m.6300 | Feruoyl Esterase | Piromyces equi | No | -0.74 | -0.41 | 1.81 | 1.17 | 0.73 | 0.43 | 0.55 | 2.55 | 1.63 |

^a^ Fold change is shown as Log_2_ expression levels compared to glucose. Color code: Green= significantly over-expressed (a differential expression p-value <0. 1 as calculated by the nbinomTest function in the R package DESeq), red= significantly under-expressed (a differential expression p-value <0.1 as calculated by the nbinomTest function in the R package DESeq).

b: Corrected FPKM values normalized by the library size, as calculated using the estimateDispersions function in the R package DESeq.

**Table S3. Mechanisms, structures, and preferred targets of key CAZymes identified in C1A transcriptome**

| **Activity** | **GH** | **Mechanism** | **Structure** | **Preferred target** |
| --- | --- | --- | --- | --- |
| Endoglucanases  EC 3.2.1.4 | GH45 | Inverting | Six stranded β barrel to which a seventh strand is appended | Internal β-1,4-glycosidic bonds in cellulose chains |
|  | GH5 | Retaining | (α/β)_8_ TIM barrel |  |
|  | GH9 | Inverting | (α/α )_6_ barrel |  |
|  | GH8 | Inverting | (α/α )_6_ barrel |  |
|  | GH124 | Inverting | Superhelical: multiple α-helices encircling a central helix with the catalytic center |  |
| Cellobiohydrolase  EC 3.2.1.91 | GH6 | Inverting | α/β barrel fold, with seven β-strands forming the central β-barrel | β-1,4-glycosidic bonds at the non-reducing ends of cellulose chains |
|  | GH48 | Retaining | (α/β)_8_ TIM barrel |  |
| β-glucosidase  EC 3.2.1.21 | GH1 | Retaining | (α/β)_8_ TIM barrel | β-1,4-glycosidic bond in cellobiose |
|  | GH3 | Retaining | Unclear |  |
|  |  |  |  |  |
| Xylanase | GH10 | Retaining | (α/β)_8_ TIM barrel | β-1,4 glycosidic bonds in oligoxylans, xylans, and substituted xylans |
|  | GH11 | Retaining | β-jelly roll | β-1,4 glycosidic bonds in xylan backbones |
| Xylosidase | GH39 | Retaining | (α/β)_8_ TIM barrel | β-1,4 glycosidic bonds in xylooligosaccharides |
|  | GH43 | Inverting | 5-fold β-propeller |  |
|  |  |  |  |  |
| Amylase | GH13 | Retaining | (α/β)_8_ TIM barrel | α-1,4 glucosidic bonds in starch |
|  | GH57 | Retaining | (β/α)_7_  barrel |  |
|  | GH119 | Retaining | (β/α)_7_  barrel |  |
|  |  |  |  |  |

**Table S4. Transcription levels of all fungal docekerin domain-containing transcripts when grown on glucose and lignocellulosic biomass substrates.**

| Transcript ID | GH family | Activity | Average Absolute FKPM Corrected | | | | | Log2 transcription level (log2(ratio FKPM biomass: FKPM glucose)^a^ | | | |
| --- | --- | --- | --- | --- | --- | --- | --- | --- | --- | --- | --- |
|  |  |  | **Glucose** | **Alfalfa** | **Energy Cane** | **Corn Stover** | **Sorghum** | **Alfalfa** | **Energy Cane** | **Corn Stover** | **Sorghum** |
| m.10020 | No | Conserved hypothetical protein | 7.68 | 3.33 | 4.29 | 1.12 | 13.40 | -1.21 | -0.84 | -2.77 | 0.80 |
| m.1004 | No | Conserved hypothetical protein | 0.97 | 7.94 | 5.08 | 3.80 | 1.99 | 3.03 | 2.38 | 1.97 | 1.03 |
| m.10379 | No | COTH-Superfamily Protein | 4.85 | 0.07 | 0.07 | 0.06 | 0.08 | -6.04 | -6.16 | -6.35 | -5.99 |
| m.10488 | No | Conserved hypothetical protein | 0.00 | 0.67 | 3.33 | 17.41 | 8.48 | 19.35 | 21.67 | 24.05 | 23.02 |
| m.10544 | No | Hypothetical Unknown | 10.61 | 6.20 | 2.86 | 2.40 | 8.38 | -0.78 | -1.89 | -2.14 | -0.34 |
| m.10579 | No | COTH-Superfamily Protein | 1.08 | 3.96 | 0.66 | 0.85 | 0.77 | 1.87 | -0.72 | -0.35 | -0.49 |
| m.10586 | No | COTH-Superfamily Protein | 1.43 | 0.45 | 2.19 | 9.86 | 2.99 | -1.67 | 0.61 | 2.78 | 1.06 |
| m.10587 | No | Conserved hypothetical protein | 9.74 | 8.30 | 6.10 | 4.22 | 6.04 | -0.23 | -0.67 | -1.21 | -0.69 |
| m.10588 | No | Conserved hypothetical protein | 4.19 | 0.19 | 1.48 | 11.47 | 2.97 | -4.45 | -1.50 | 1.45 | -0.50 |
| m.1059 | No | COTH-Superfamily Protein | 0.63 | 1.85 | 0.16 | 0.05 | 1.19 | 1.56 | -1.98 | -3.64 | 0.92 |
| m.10635 | No | Predicted ser/Thr phosphohydrolase | 13.92 | 8.95 | 7.59 | 1.83 | 21.32 | -0.64 | -0.88 | -2.93 | 0.62 |
| m.1118 | No | CE:Carbhydrate Estrase | 7.71 | 8.87 | 4.32 | 4.39 | 6.26 | 0.20 | -0.84 | -0.81 | -0.30 |
| m.11359 | No | Predicted ser/Thr phosphohydrolase | 0.65 | 0.73 | 1.93 | 4.44 | 3.03 | 0.16 | 1.57 | 2.77 | 2.22 |
| m.11360 | No | Predicted ser/Thr phosphohydrolase | 4.48 | 16.78 | 5.04 | 0.82 | 4.85 | 1.91 | 0.17 | -2.46 | 0.12 |
| m.11386 | No | COTH-Superfamily Protein | 0.00 | 1.19 | 0.18 | 0.00 | 0.06 | 20.19 | 17.49 | 0.00 | 15.96 |
| m.11625 | No | Conserved hypothetical protein | 0.00 | 0.00 | 1.17 | 2.93 | 0.89 | 0.00 | 20.16 | 21.48 | 19.77 |
| m.11779 | No | Predicted ser/Thr phosphohydrolase | 0.67 | 0.27 | 1.06 | 1.36 | 1.31 | -1.29 | 0.67 | 1.02 | 0.97 |
| m.11783 | No | Predicted ser/Thr phosphohydrolase | 2.15 | 3.83 | 4.38 | 11.08 | 2.99 | 0.83 | 1.03 | 2.36 | 0.48 |
| m.11871 | No | COTH-Superfamily Protein | 0.00 | 0.23 | 1.71 | 0.12 | 0.22 | 17.83 | 20.71 | 16.89 | 17.78 |
| m.12430 | No | COTH-Superfamily Protein | 5.82 | 6.98 | 2.47 | 0.58 | 5.58 | 0.26 | -1.23 | -3.33 | -0.06 |
| m.12573 | Yes | GH5: Endoglucanase | 0.30 | 0.15 | 1.37 | 0.18 | 0.49 | -0.99 | 2.17 | -0.75 | 0.70 |
| m.12576 | Yes | GH5: Endoglucanase | 0.00 | 0.50 | 0.89 | 0.20 | 0.17 | 18.93 | 19.77 | 17.61 | 17.40 |
| m.12584 | Yes | GH5: Endoglucanase | 5.51 | 9.26 | 4.60 | 8.04 | 4.53 | 0.75 | -0.26 | 0.55 | -0.28 |
| m.12594 | Yes | GH5: Endoglucanase | 3.73 | 3.02 | 1.84 | 5.31 | 3.28 | -0.30 | -1.02 | 0.51 | -0.19 |
| m.12908 | No | COTH-Superfamily Protein | 0.94 | 3.26 | 1.93 | 2.59 | 1.93 | 1.80 | 1.04 | 1.47 | 1.04 |
| m.12909 | Yes | GH10: endo-1 4-beta-xylanase | 0.72 | 6.06 | 3.83 | 0.63 | 0.34 | 3.07 | 2.41 | -0.21 | -1.09 |
| m.1291 | No | COTH-Superfamily Protein | 71.71 | 3.56 | 11.12 | 10.14 | 8.69 | -4.33 | -2.69 | -2.82 | -3.04 |
| m.13139 | No | COTH-Superfamily Protein | 0.00 | 0.00 | 0.54 | 0.24 | 0.35 | 0.00 | 19.04 | 17.90 | 18.43 |
| m.14104 | No | COTH-Superfamily Protein | 0.00 | 0.00 | 0.27 | 0.12 | 0.84 | 0.00 | 18.06 | 16.86 | 19.69 |
| m.1431 | No | Hypothetical Unknown | 6.84 | 2.40 | 1.36 | 1.89 | 3.87 | -1.51 | -2.33 | -1.85 | -0.82 |
| m.14566 | No | Conserved hypothetical protein | 0.86 | 2.60 | 1.97 | 2.51 | 4.57 | 1.59 | 1.19 | 1.54 | 2.40 |
| m.14831 | Yes | CE:Acetylxylan Esterase | 8.11 | 22.91 | 11.75 | 21.08 | 15.39 | 1.50 | 0.53 | 1.38 | 0.92 |
| m.14997 | No | COTH-Superfamily Protein | 1.13 | 0.35 | 2.65 | 3.76 | 1.44 | -1.69 | 1.24 | 1.74 | 0.36 |
| m.15105 | Yes | GH48: CellobiohydrolaseGH48 | 5.08 | 0.00 | 0.00 | 0.35 | 0.00 | -22.28 | -22.28 | -3.86 | -22.28 |
| m.15107 | Yes | GH48: CellobiohydrolaseGH48 | 3.20 | 2.45 | 4.97 | 1.64 | 0.87 | -0.39 | 0.64 | -0.97 | -1.87 |
| m.15108 | Yes | GH48: CellobiohydrolaseGH48 | 0.44 | 0.00 | 1.30 | 0.23 | 0.45 | -18.73 | 1.57 | -0.92 | 0.05 |
| m.15243 | No | COTH-Superfamily Protein | 0.84 | 3.00 | 2.55 | 0.09 | 0.17 | 1.84 | 1.60 | -3.26 | -2.32 |
| m.15247 | No | COTH-Superfamily Protein | 9.83 | 0.95 | 4.74 | 1.30 | 1.17 | -3.37 | -1.05 | -2.91 | -3.07 |
| m.1578 | No | COTH-Superfamily Protein | 2.10 | 0.13 | 0.21 | 1.70 | 1.30 | -4.07 | -3.31 | -0.31 | -0.70 |
| m.15995 | No | Hypothetical Unknown | 2.70 | 13.90 | 3.38 | 1.33 | 0.59 | 2.36 | 0.32 | -1.02 | -2.20 |
| m.15996 | No | Hypothetical Unknown | 2.16 | 3.66 | 2.73 | 5.74 | 3.96 | 0.76 | 0.34 | 1.41 | 0.87 |
| m.16194 | No | COTH-Superfamily Protein | 0.96 | 0.20 | 0.42 | 0.16 | 3.21 | -2.26 | -1.18 | -2.58 | 1.75 |
| m.1624 | No | COTH-Superfamily Protein | 6.84 | 0.39 | 1.01 | 1.80 | 2.35 | -4.13 | -2.76 | -1.93 | -1.54 |
| m.16583 | No | COTH-Superfamily Protein | 5.64 | 7.32 | 16.28 | 2.05 | 4.51 | 0.38 | 1.53 | -1.46 | -0.32 |
| m.16584 | No | COTH-Superfamily Protein | 3.81 | 10.10 | 14.52 | 1.01 | 2.41 | 1.40 | 1.93 | -1.92 | -0.66 |
| m.1666 | No | COTH-Superfamily Protein | 0.26 | 4.32 | 4.05 | 1.10 | 7.63 | 4.05 | 3.96 | 2.08 | 4.87 |
| m.16727 | No | CE:Feruoyl Esterase | 0.00 | 0.37 | 0.66 | 2.10 | 10.91 | 18.50 | 19.33 | 21.00 | 23.38 |
| m.17073 | No | COTH-Superfamily Protein | 0.44 | 3.03 | 1.73 | 0.55 | 0.35 | 2.79 | 1.99 | 0.33 | -0.31 |
| m.17074 | No | COTH-Superfamily Protein | 7.89 | 59.34 | 18.15 | 5.07 | 7.64 | 2.91 | 1.20 | -0.64 | -0.05 |
| m.17274 | No | COTH-Superfamily Protein | 3.90 | 0.64 | 0.46 | 0.97 | 7.44 | -2.61 | -3.09 | -2.01 | 0.93 |
| m.17280 | No | COTH-Superfamily Protein | 13.93 | 1.00 | 0.48 | 3.58 | 11.40 | -3.80 | -4.85 | -1.96 | -0.29 |
| m.17282 | No | COTH-Superfamily Protein | 1.05 | 2.42 | 4.23 | 3.04 | 8.22 | 1.20 | 2.00 | 1.53 | 2.96 |
| m.17284 | No | COTH-Superfamily Protein | 117.05 | 42.46 | 19.38 | 5.02 | 68.38 | -1.46 | -2.59 | -4.54 | -0.78 |
| m.17286 | No | COTH-Superfamily Protein | 0.25 | 0.85 | 10.67 | 6.00 | 41.76 | 1.78 | 5.43 | 4.60 | 7.40 |
| m.17289 | No | COTH-Superfamily Protein | 0.70 | 0.17 | 0.15 | 3.30 | 0.56 | -2.04 | -2.25 | 2.24 | -0.33 |
| m.17297 | No | COTH-Superfamily Protein | 0.00 | 0.47 | 0.19 | 3.92 | 0.00 | 18.84 | 17.50 | 21.90 | 0.00 |
| m.17428 | No | Alkyl transferase | 0.27 | 0.76 | 32.47 | 6.18 | 20.05 | 1.49 | 6.90 | 4.51 | 6.21 |
| m.17507 | No | CE:Carbhydrate Estrase | 6.02 | 19.09 | 12.78 | 10.32 | 10.55 | 1.66 | 1.08 | 0.78 | 0.81 |
| m.17600 | No | Hypothetical Unknown | 17.72 | 34.42 | 9.58 | 4.64 | 3.87 | 0.96 | -0.89 | -1.93 | -2.20 |
| m.17601 | No | Hypothetical Unknown | 5.05 | 6.70 | 3.93 | 1.45 | 2.14 | 0.41 | -0.36 | -1.80 | -1.24 |
| m.17603 | No | Hypothetical Unknown | 12.60 | 24.10 | 19.25 | 16.97 | 7.46 | 0.94 | 0.61 | 0.43 | -0.76 |
| m.17605 | No | Hypothetical Unknown | 0.00 | 3.17 | 0.00 | 0.00 | 0.00 | 21.60 | 0.00 | 0.00 | 0.00 |
| m.17720 | No | Hypothetical Unknown | 22.64 | 0.00 | 0.16 | 0.13 | 0.12 | -24.43 | -7.12 | -7.42 | -7.59 |
| m.17725 | No | Hypothetical Unknown | 26.63 | 0.16 | 0.72 | 0.25 | 0.37 | -7.34 | -5.21 | -6.72 | -6.17 |
| m.17946 | Yes | GH9: endoglucanase | 149.41 | 195.41 | 185.57 | 218.77 | 128.88 | 0.39 | 0.31 | 0.55 | -0.21 |
| m.17949 | Yes | GH9: endoglucanase | 67.58 | 233.45 | 346.66 | 574.35 | 615.08 | 1.79 | 2.36 | 3.09 | 3.19 |
| m.18232 | No | Hypothetical Unknown | 0.55 | 5.41 | 1.71 | 0.03 | 0.09 | 3.29 | 1.63 | -4.39 | -2.57 |
| m.18404 | Yes | Dual: Feruoyl Esterase and GH 45 | 17.95 | 17.95 | 28.44 | 13.34 | 45.50 | 0.00 | 0.66 | -0.43 | 1.34 |
| m.1853 | Yes | GH45: Endoglucanase | 1.75 | 4.49 | 3.59 | 3.65 | 2.04 | 1.36 | 1.04 | 1.06 | 0.23 |
| m.18549 | Yes | GH43: alpha-N-arabinofuranosidase | 4.95 | 28.23 | 11.42 | 20.25 | 16.05 | 2.51 | 1.21 | 2.03 | 1.70 |
| m.18689 | No | Swollening/Expansion | 457.30 | 271.19 | 202.10 | 248.32 | 376.53 | -0.75 | -1.18 | -0.88 | -0.28 |
| m.18700 | No | COTH-Superfamily Protein | 0.61 | 0.26 | 2.89 | 0.82 | 0.33 | -1.24 | 2.24 | 0.43 | -0.88 |
| m.18765 | No | COTH-Superfamily Protein | 3.88 | 22.11 | 8.73 | 1.63 | 1.70 | 2.51 | 1.17 | -1.25 | -1.19 |
| m.19090 | No | COTH-Superfamily Protein | 2.81 | 13.21 | 174.11 | 173.82 | 371.56 | 2.23 | 5.95 | 5.95 | 7.05 |
| m.19362 | Yes | GH26:beta-mannanase | 3.41 | 5.78 | 4.94 | 7.44 | 5.00 | 0.76 | 0.53 | 1.12 | 0.55 |
| m.19363 | Yes | GH5: Endoglucanase | 28.41 | 26.71 | 22.20 | 28.08 | 9.28 | -0.09 | -0.36 | -0.02 | -1.61 |
| m.19364 | Yes | GH26:beta-mannanase | 0.00 | 0.81 | 3.99 | 6.75 | 1.06 | 19.62 | 21.93 | 22.69 | 20.02 |
| m.19659 | No | COTH-Superfamily Protein | 286.49 | 228.83 | 100.22 | 45.52 | 212.53 | -0.32 | -1.52 | -2.65 | -0.43 |
| m.19660 | Yes | Dual:Carbohydrate Estrase And GH9 | 15.06 | 35.44 | 18.84 | 8.01 | 51.05 | 1.23 | 0.32 | -0.91 | 1.76 |
| m.19661 | No | COTH-Superfamily Protein | 0.00 | 0.00 | 0.00 | 0.08 | 10.44 | 0.00 | 0.00 | 16.20 | 23.32 |
| m.19662 | Yes | Dual:Carbohydrate Estrase And GH9 | 57.73 | 81.51 | 22.40 | 7.81 | 49.95 | 0.50 | -1.37 | -2.89 | -0.21 |
| m.19663 | No | COTH-Superfamily Protein | 9.35 | 3.40 | 0.15 | 1.76 | 5.81 | -1.46 | -6.00 | -2.41 | -0.68 |
| m.19665 | No | COTH-Superfamily Protein | 15.92 | 12.36 | 8.82 | 5.62 | 3.85 | -0.37 | -0.85 | -1.50 | -2.05 |
| m.19666 | Yes | GH:xyloglucanase | 9.71 | 16.49 | 13.11 | 28.23 | 11.50 | 0.76 | 0.43 | 1.54 | 0.24 |
| m.19667 | Yes | GH:xyloglucanase | 37.40 | 42.84 | 28.08 | 19.10 | 4.96 | 0.20 | -0.41 | -0.97 | -2.91 |
| m.19879 | No | COTH-Superfamily Protein | 5.13 | 0.32 | 3.99 | 1.30 | 0.63 | -4.01 | -0.36 | -1.98 | -3.02 |
| m.19881 | No | COTH-Superfamily Protein | 23.01 | 10.27 | 8.54 | 6.84 | 3.67 | -1.16 | -1.43 | -1.75 | -2.65 |
| m.19929 | No | COTH-Superfamily Protein | 9.34 | 25.93 | 19.55 | 18.30 | 7.24 | 1.47 | 1.07 | 0.97 | -0.37 |
| m.19940 | No | COTH-Superfamily Protein | 1.23 | 10.15 | 3.99 | 12.12 | 14.58 | 3.05 | 1.70 | 3.30 | 3.57 |
| m.19942 | Yes | GH48: CellobiohydrolaseGH48 | 2597.78 | 316.08 | 92.74 | 37.72 | 164.74 | -3.04 | -4.81 | -6.11 | -3.98 |
| m.19944 | Yes | GH48: CellobiohydrolaseGH48 | 3.11 | 21.52 | 13.76 | 5.97 | 51.63 | 2.79 | 2.14 | 0.94 | 4.05 |
| m.19946 | No | COTH-Superfamily Protein | 123.91 | 80.14 | 89.50 | 173.62 | 96.62 | -0.63 | -0.47 | 0.49 | -0.36 |
| m.19947 | No | Hypothetical Unknown | 25.51 | 15.00 | 33.99 | 46.62 | 44.14 | -0.77 | 0.41 | 0.87 | 0.79 |
| m.19948 | No | COTH-Superfamily Protein | 380.24 | 169.56 | 200.57 | 161.41 | 132.09 | -1.17 | -0.92 | -1.24 | -1.53 |
| m.19952 | Yes | GH48: CellobiohydrolaseGH48 | 2.63 | 30.16 | 15.05 | 34.00 | 30.16 | 3.52 | 2.52 | 3.69 | 3.52 |
| m.19954 | No | Predicted ser/Thr phosphohydrolase | 11.56 | 3.20 | 57.23 | 121.42 | 28.83 | -1.85 | 2.31 | 3.39 | 1.32 |
| m.19958 | No | Predicted ser/Thr phosphohydrolase | 71.65 | 10.53 | 23.36 | 22.95 | 24.38 | -2.77 | -1.62 | -1.64 | -1.56 |
| m.19961 | No | Predicted ser/Thr phosphohydrolase | 11.91 | 6.99 | 21.67 | 33.13 | 26.82 | -0.77 | 0.86 | 1.48 | 1.17 |
| m.20092 | No | COTH-Superfamily Protein | 3.05 | 2.48 | 0.47 | 1.28 | 0.74 | -0.30 | -2.69 | -1.25 | -2.04 |
| m.20206 | No | COTH-Superfamily Protein | 2.57 | 0.00 | 0.18 | 0.13 | 0.95 | -21.29 | -3.86 | -4.34 | -1.44 |
| m.20268 | No | COTH-Superfamily Protein | 0.41 | 0.41 | 0.94 | 0.20 | 0.91 | -0.02 | 1.19 | -1.06 | 1.14 |
| m.20422 | No | Proteease | 223.21 | 25.00 | 1.94 | 2.61 | 1.77 | -3.16 | -6.85 | -6.42 | -6.98 |
| m.20626 | Yes | GH11: endo-1 4-beta-xylanase | 13.47 | 0.74 | 1.42 | 2.37 | 2.78 | -4.18 | -3.24 | -2.51 | -2.28 |
| m.20630 | Yes | GH11: endo-1 4-beta-xylanase | 35.65 | 0.93 | 2.33 | 2.38 | 2.95 | -5.26 | -3.94 | -3.90 | -3.59 |
| m.2075 | No | CE:Feruoyl Esterase | 0.16 | 4.71 | 3.84 | 1.13 | 2.15 | 4.88 | 4.58 | 2.82 | 3.75 |
| m.20858 | Yes | GH26:beta-mannanase | 91.84 | 55.43 | 10.31 | 27.05 | 6.40 | -0.73 | -3.15 | -1.76 | -3.84 |
| m.20862 | Yes | GH26:beta-mannanase | 14.91 | 38.60 | 15.23 | 18.68 | 13.95 | 1.37 | 0.03 | 0.33 | -0.10 |
| m.20866 | Yes | GH10: endo-1 4-beta-xylanase | 504.02 | 283.47 | 78.80 | 90.24 | 197.21 | -0.83 | -2.68 | -2.48 | -1.35 |
| m.20869 | Yes | GH48: CellobiohydrolaseGH48 | 114.39 | 630.97 | 446.50 | 255.06 | 142.49 | 2.46 | 1.96 | 1.16 | 0.32 |
| m.20871 | Yes | GH48: CellobiohydrolaseGH48 | 20.14 | 74.80 | 28.94 | 46.11 | 32.44 | 1.89 | 0.52 | 1.20 | 0.69 |
| m.20872 | Yes | GH10: endo-1 4-beta-xylanase | 0.76 | 0.36 | 0.10 | 0.58 | 1.34 | -1.06 | -2.86 | -0.39 | 0.82 |
| m.20873 | No | Serpin (Protease inhibitor) | 9.37 | 2.11 | 8.02 | 3.76 | 2.46 | -2.15 | -0.22 | -1.32 | -1.93 |
| m.20876 | Yes | Dual: Polysacchride Deacetylase and GH 5 | 541.90 | 273.11 | 338.10 | 351.91 | 560.12 | -0.99 | -0.68 | -0.62 | 0.05 |
| m.20877 | Yes | GH48: CellobiohydrolaseGH48 | 3.53 | 27.64 | 9.70 | 7.77 | 5.00 | 2.97 | 1.46 | 1.14 | 0.50 |
| m.20878 | Yes | GH48: CellobiohydrolaseGH48 | 28.75 | 173.70 | 88.62 | 38.44 | 23.85 | 2.60 | 1.62 | 0.42 | -0.27 |
| m.20879 | No | Serpin (Protease inhibitor) | 40.22 | 16.93 | 40.41 | 38.51 | 46.26 | -1.25 | 0.01 | -0.06 | 0.20 |
| m.20882 | No | COTH-Superfamily Protein | 34.82 | 34.06 | 64.00 | 72.45 | 70.78 | -0.03 | 0.88 | 1.06 | 1.02 |
| m.20883 | No | Serpin (Protease inhibitor) | 156.79 | 82.68 | 127.13 | 81.28 | 76.81 | -0.92 | -0.30 | -0.95 | -1.03 |
| m.20884 | Yes | GH45: Endoglucanase | 77.69 | 50.51 | 84.27 | 166.97 | 61.12 | -0.62 | 0.12 | 1.10 | -0.35 |
| m.20885 | No | CE:Acetylxylan Esterase | 975.72 | 341.01 | 391.33 | 510.55 | 671.25 | -1.52 | -1.32 | -0.93 | -0.54 |
| m.20887 | No | Serpin (Protease inhibitor) | 0.08 | 0.47 | 3.49 | 2.26 | 1.29 | 2.52 | 5.40 | 4.78 | 3.97 |
| m.20888 | Yes | GH48: CellobiohydrolaseGH48 | 12.19 | 65.16 | 38.40 | 15.43 | 9.84 | 2.42 | 1.66 | 0.34 | -0.31 |
| m.20891 | Yes | GH48: CellobiohydrolaseGH48 | 74.90 | 44.20 | 143.17 | 105.32 | 49.03 | -0.76 | 0.93 | 0.49 | -0.61 |
| m.20892 | No | COTH-Superfamily Protein | 11.97 | 65.47 | 15.99 | 16.10 | 10.21 | 2.45 | 0.42 | 0.43 | -0.23 |
| m.20894 | No | COTH-Superfamily Protein | 19.35 | 0.00 | 0.73 | 1.90 | 1.07 | -24.21 | -4.73 | -3.35 | -4.18 |
| m.20897 | No | Serpin (Protease inhibitor) | 10.86 | 2.90 | 4.63 | 14.21 | 14.12 | -1.91 | -1.23 | 0.39 | 0.38 |
| m.20899 | No | COTH-Superfamily Protein | 72.58 | 92.62 | 305.28 | 371.43 | 381.08 | 0.35 | 2.07 | 2.36 | 2.39 |
| m.20902 | No | COTH-Superfamily Protein | 247.96 | 415.40 | 377.59 | 374.92 | 335.35 | 0.74 | 0.61 | 0.60 | 0.44 |
| m.20903 | No | Serpin (Protease inhibitor) | 35.58 | 16.49 | 59.79 | 30.89 | 68.81 | -1.11 | 0.75 | -0.20 | 0.95 |
| m.20904 | No | Serpin (Protease inhibitor) | 41.14 | 24.13 | 41.31 | 21.60 | 16.26 | -0.77 | 0.01 | -0.93 | -1.34 |
| m.20906 | Yes | GH10: endo-1 4-beta-xylanase | 163.67 | 316.90 | 221.53 | 212.19 | 212.03 | 0.95 | 0.44 | 0.37 | 0.37 |
| m.20907 | No | Rhamnogalacturonate lyase | 51.09 | 41.74 | 61.13 | 78.81 | 102.56 | -0.29 | 0.26 | 0.63 | 1.01 |
| m.20908 | No | CE: Polysacchride Deacetylase | 3.46 | 0.43 | 0.60 | 0.51 | 0.99 | -3.00 | -2.53 | -2.77 | -1.81 |
| m.20909 | No | Serpin (Protease inhibitor) | 8.36 | 1.95 | 15.16 | 4.84 | 5.21 | -2.10 | 0.86 | -0.79 | -0.68 |
| m.20910 | Yes | GH48: CellobiohydrolaseGH48 | 0.00 | 2.35 | 3.75 | 11.83 | 4.95 | 21.16 | 21.84 | 23.50 | 22.24 |
| m.20911 | No | Serpin (Protease inhibitor) | 9.46 | 2.59 | 19.46 | 6.98 | 11.56 | -1.87 | 1.04 | -0.44 | 0.29 |
| m.20914 | No | Serpin (Protease inhibitor) | 28.62 | 11.11 | 27.83 | 44.67 | 69.63 | -1.37 | -0.04 | 0.64 | 1.28 |
| m.21122 | Yes | GH11: endo-1 4-beta-xylanase | 6.04 | 3.55 | 0.90 | 1.74 | 4.67 | -0.77 | -2.74 | -1.80 | -0.37 |
| m.21128 | Yes | GH11: endo-1 4-beta-xylanase | 137.08 | 88.92 | 103.74 | 158.77 | 160.71 | -0.62 | -0.40 | 0.21 | 0.23 |
| m.21130 | Yes | GH11: endo-1 4-beta-xylanase | 2.67 | 2.48 | 1.85 | 5.91 | 4.01 | -0.11 | -0.53 | 1.15 | 0.59 |
| m.21473 | Yes | GH: xylosidase/arabinofuranosidase | 76.44 | 123.86 | 9.10 | 2.85 | 13.21 | 0.70 | -3.07 | -4.74 | -2.53 |
| m.21519 | No | COTH-Superfamily Protein | 127.01 | 37.45 | 16.79 | 3.23 | 12.42 | -1.76 | -2.92 | -5.30 | -3.35 |
| m.21522 | No | COTH-Superfamily Protein | 149.15 | 177.29 | 95.68 | 115.75 | 108.58 | 0.25 | -0.64 | -0.37 | -0.46 |
| m.21525 | Yes | GH9: endoglucanase | 57.47 | 44.44 | 87.40 | 212.25 | 94.68 | -0.37 | 0.60 | 1.88 | 0.72 |
| m.21531 | Yes | GH9: endoglucanase | 32.05 | 14.10 | 14.91 | 26.30 | 38.22 | -1.18 | -1.10 | -0.29 | 0.25 |
| m.21541 | Yes | GH9: endoglucanase | 3.84 | 3.49 | 3.88 | 45.63 | 0.84 | -0.14 | 0.02 | 3.57 | -2.19 |
| m.21547 | Yes | GH9: endoglucanase | 30.28 | 38.41 | 48.00 | 46.91 | 53.69 | 0.34 | 0.66 | 0.63 | 0.83 |
| m.21903 | No | COTH-Superfamily Protein | 96.04 | 63.08 | 51.51 | 115.45 | 58.80 | -0.61 | -0.90 | 0.27 | -0.71 |
| m.21905 | No | COTH-Superfamily Protein | 157.40 | 120.43 | 85.29 | 101.96 | 86.94 | -0.39 | -0.88 | -0.63 | -0.86 |
| m.21936 | No | Swollenin/Expansin | 270.84 | 110.26 | 216.87 | 567.06 | 323.45 | -1.30 | -0.32 | 1.07 | 0.26 |
| m.21939 | No | Swollenin/Expansin | 30.23 | 0.00 | 0.00 | 0.00 | 0.00 | -24.85 | -24.85 | -24.85 | -24.85 |
| m.21943 | No | Swollenin/Expansion | 170.11 | 140.67 | 218.65 | 269.19 | 203.41 | -0.27 | 0.36 | 0.66 | 0.26 |
| m.22176 | No | COTH-Superfamily Protein | 151.86 | 255.30 | 140.88 | 105.48 | 82.09 | 0.75 | -0.11 | -0.53 | -0.89 |
| m.22178 | No | COTH-Superfamily Protein | 53.35 | 467.42 | 208.75 | 89.59 | 111.06 | 3.13 | 1.97 | 0.75 | 1.06 |
| m.22179 | No | COTH-Superfamily Protein | 87.38 | 110.17 | 94.19 | 187.95 | 128.84 | 0.33 | 0.11 | 1.10 | 0.56 |
| m.22184 | Yes | GH45: Endoglucanase | 35.19 | 41.20 | 56.30 | 98.51 | 43.06 | 0.23 | 0.68 | 1.49 | 0.29 |
| m.22205 | Yes | GH45: Endoglucanase | 31.65 | 6.93 | 9.79 | 5.16 | 12.86 | -2.19 | -1.69 | -2.62 | -1.30 |
| m.22208 | No | COTH-Superfamily Protein | 187.47 | 59.85 | 30.92 | 13.15 | 34.12 | -1.65 | -2.60 | -3.83 | -2.46 |
| m.22216 | Yes | GH45: Endoglucanase | 9.88 | 11.14 | 4.85 | 8.45 | 8.55 | 0.17 | -1.03 | -0.23 | -0.21 |
| m.22235 | No | COTH-Superfamily Protein | 0.00 | 0.12 | 7.44 | 0.59 | 2.60 | 16.87 | 22.83 | 19.18 | 21.31 |
| m.22707 | No | COTH-Superfamily Protein | 3.27 | 3.88 | 4.43 | 12.77 | 4.08 | 0.25 | 0.44 | 1.96 | 0.32 |
| m.22925 | No | COTH-Superfamily Protein | 3.79 | 6.14 | 9.83 | 16.12 | 25.94 | 0.70 | 1.38 | 2.09 | 2.78 |
| m.22926 | No | Conserved hypothetical protein | 191.74 | 65.39 | 85.65 | 91.56 | 49.81 | -1.55 | -1.16 | -1.07 | -1.94 |
| m.22928 | Yes | GH45: Endoglucanase | 295.19 | 153.20 | 70.06 | 121.85 | 204.77 | -0.95 | -2.07 | -1.28 | -0.53 |
| m.22935 | Yes | GH5: endoglucanase | 4.81 | 0.50 | 8.55 | 13.49 | 0.00 | -3.27 | 0.83 | 1.49 | -22.20 |
| m.22936 | Yes | GH5: endoglucanase | 0.33 | 5.13 | 3.43 | 15.41 | 9.95 | 3.95 | 3.37 | 5.53 | 4.90 |
| m.22939 | Yes | GH26:beta-mannanase | 4.45 | 15.86 | 13.34 | 20.50 | 15.33 | 1.83 | 1.58 | 2.20 | 1.78 |
| m.22941 | Yes | GH45: Endoglucanase | 0.00 | 0.00 | 0.00 | 0.00 | 36.55 | 0.00 | 0.00 | 0.00 | 25.12 |
| m.22945 | No | Conserved hypothetical protein | 84.29 | 87.55 | 17.52 | 8.28 | 82.18 | 0.05 | -2.27 | -3.35 | -0.04 |
| m.22948 | Yes | GH45: Endoglucanase | 0.00 | 0.00 | 0.00 | 0.00 | 5.31 | 0.00 | 0.00 | 0.00 | 22.34 |
| m.22950 | Yes | GH45: Endoglucanase | 108.89 | 126.32 | 297.77 | 361.72 | 165.34 | 0.21 | 1.45 | 1.73 | 0.60 |
| m.22953 | Yes | GH5: endoglucanase | 6.42 | 36.26 | 7.82 | 44.18 | 19.08 | 2.50 | 0.28 | 2.78 | 1.57 |
| m.22956 | No | Conserved hypothetical protein | 168.45 | 99.42 | 218.35 | 261.19 | 300.79 | -0.76 | 0.37 | 0.63 | 0.84 |
| m.22957 | Yes | GH5: endoglucanase | 0.00 | 5.62 | 9.13 | 16.21 | 8.85 | 22.42 | 23.12 | 23.95 | 23.08 |
| m.22959 | Yes | GH5: endoglucanase | 18.18 | 4.89 | 32.77 | 33.75 | 0.00 | -1.89 | 0.85 | 0.89 | -24.12 |
| m.22960 | Yes | GH5: endoglucanase | 0.61 | 2.25 | 1.93 | 7.56 | 7.34 | 1.87 | 1.65 | 3.62 | 3.58 |
| m.22961 | Yes | GH5: endoglucanase | 0.00 | 2.14 | 4.56 | 6.08 | 4.00 | 21.03 | 22.12 | 22.53 | 21.93 |
| m.23163 | No | COTH-Superfamily Protein | 22.75 | 11.58 | 22.83 | 10.99 | 17.25 | -0.97 | 0.01 | -1.05 | -0.40 |
| m.23169 | No | COTH-Superfamily Protein | 0.19 | 0.49 | 5.01 | 0.20 | 1.42 | 1.42 | 4.76 | 0.11 | 2.94 |
| m.23171 | No | COTH-Superfamily Protein | 0.22 | 0.00 | 1.43 | 0.80 | 3.33 | -17.76 | 2.69 | 1.84 | 3.91 |
| m.23176 | No | COTH-Superfamily Protein | 1.99 | 3.68 | 7.40 | 2.63 | 18.84 | 0.89 | 1.90 | 0.40 | 3.24 |
| m.23177 | No | COTH-Superfamily Protein | 5.47 | 3.12 | 3.57 | 5.85 | 5.26 | -0.81 | -0.61 | 0.10 | -0.05 |
| m.23469 | Yes | GH45: Endoglucanase | 256.51 | 111.79 | 82.98 | 83.16 | 135.37 | -1.20 | -1.63 | -1.63 | -0.92 |
| m.23475 | Yes | GH45: Endoglucanase | 128.90 | 22.36 | 34.15 | 78.38 | 52.41 | -2.53 | -1.92 | -0.72 | -1.30 |
| m.24007 | Yes | GH48: CellobiohydrolaseGH48 | 136.12 | 29.34 | 192.35 | 881.24 | 368.57 | -2.21 | 0.50 | 2.69 | 1.44 |
| m.24012 | Yes | GH48: CellobiohydrolaseGH48 | 41.67 | 134.45 | 659.25 | 1322.57 | 285.09 | 1.69 | 3.98 | 4.99 | 2.77 |
| m.24017 | Yes | GH48: CellobiohydrolaseGH48 | 148.67 | 311.34 | 793.05 | 1566.10 | 544.74 | 1.07 | 2.42 | 3.40 | 1.87 |
| m.24197 | Yes | GH48: CellobiohydrolaseGH48 | 133.65 | 115.35 | 288.53 | 434.32 | 362.11 | -0.21 | 1.11 | 1.70 | 1.44 |
| m.24321 | No | COTH-Superfamily Protein | 81.44 | 62.95 | 18.43 | 42.28 | 49.18 | -0.37 | -2.14 | -0.95 | -0.73 |
| m.24439 | No | Conserved hypothetical protein | 237.70 | 96.58 | 124.34 | 222.76 | 108.93 | -1.30 | -0.93 | -0.09 | -1.13 |
| m.24496 | No | COTH-Superfamily Protein | 138.34 | 162.38 | 73.58 | 41.47 | 85.52 | 0.23 | -0.91 | -1.74 | -0.69 |
| m.24508 | No | COTH-Superfamily Protein | 267.17 | 57.80 | 53.08 | 114.81 | 211.22 | -2.21 | -2.33 | -1.22 | -0.34 |
| m.24531 | No | COTH-Superfamily Protein | 464.08 | 79.28 | 106.07 | 104.43 | 57.53 | -2.55 | -2.13 | -2.15 | -3.01 |
| m.24613 | No | COTH-Superfamily Protein | 38.35 | 2.87 | 14.41 | 55.97 | 21.50 | -3.74 | -1.41 | 0.55 | -0.83 |
| m.24633 | No | Hypothetical Unknown | 23.26 | 77.95 | 50.17 | 54.87 | 41.43 | 1.74 | 1.11 | 1.24 | 0.83 |
| m.24703 | No | COTH-Superfamily Protein | 106.81 | 47.95 | 51.00 | 40.00 | 37.01 | -1.16 | -1.07 | -1.42 | -1.53 |
| m.24791 | No | Conserved hypothetical protein | 48.04 | 92.49 | 31.55 | 58.90 | 47.88 | 0.95 | -0.61 | 0.29 | 0.00 |
| m.24863 | No | COTH-Superfamily Protein | 63.87 | 21.24 | 42.85 | 15.02 | 11.79 | -1.59 | -0.58 | -2.09 | -2.44 |
| m.24880 | No | Conserved hypothetical protein | 8.64 | 13.23 | 27.27 | 16.42 | 9.59 | 0.61 | 1.66 | 0.93 | 0.15 |
| m.25060 | No | COTH-Superfamily Protein | 19.40 | 1.93 | 19.03 | 13.08 | 18.81 | -3.33 | -0.03 | -0.57 | -0.04 |
| m.2522 | No | COTH-Superfamily Protein | 3.35 | 8.31 | 3.36 | 0.15 | 0.60 | 1.31 | 0.00 | -4.50 | -2.49 |
| m.25349 | No | Hypothetical Unknown | 20.41 | 22.64 | 11.65 | 15.70 | 19.65 | 0.15 | -0.81 | -0.38 | -0.05 |
| m.25369 | Yes | Conserved hypothetical protein | 146.64 | 20.96 | 7.53 | 30.58 | 5.89 | -2.81 | -4.28 | -2.26 | -4.64 |
| m.25430 | No | COTH-Superfamily Protein | 4.41 | 3.02 | 3.95 | 1.68 | 14.78 | -0.54 | -0.16 | -1.39 | 1.75 |
| m.25559 | No | COTH-Superfamily Protein | 99.61 | 7.78 | 9.72 | 9.97 | 12.99 | -3.68 | -3.36 | -3.32 | -2.94 |
| m.25872 | No | Hypothetical Unknown | 18.25 | 5.76 | 9.93 | 7.71 | 11.62 | -1.66 | -0.88 | -1.24 | -0.65 |
| m.25888 | No | Conserved hypothetical protein | 10.59 | 0.74 | 6.02 | 21.91 | 10.55 | -3.84 | -0.81 | 1.05 | 0.00 |
| m.2599 | No | CE:Acetylxylan Esterase | 49.83 | 128.38 | 184.40 | 97.72 | 171.19 | 1.37 | 1.89 | 0.97 | 1.78 |
| m.26002 | No | COTH-Superfamily Protein | 5.48 | 7.58 | 3.33 | 1.59 | 18.03 | 0.47 | -0.72 | -1.79 | 1.72 |
| m.26017 | No | Conserved hypothetical protein | 4.25 | 7.95 | 7.62 | 8.86 | 7.60 | 0.90 | 0.84 | 1.06 | 0.84 |
| m.26090 | No | COTH-Superfamily Protein | 7.45 | 5.42 | 3.12 | 0.89 | 4.55 | -0.46 | -1.25 | -3.07 | -0.71 |
| m.26101 | No | COTH-Superfamily Protein | 6.51 | 2.03 | 6.80 | 20.47 | 5.25 | -1.68 | 0.06 | 1.65 | -0.31 |
| m.26265 | No | COTH-Superfamily Protein | 46.99 | 5.84 | 2.98 | 2.64 | 23.49 | -3.01 | -3.98 | -4.16 | -1.00 |
| m.26336 | No | COTH-Superfamily Protein | 2.55 | 4.33 | 4.59 | 3.00 | 5.65 | 0.76 | 0.85 | 0.23 | 1.15 |
| m.26344 | No | COTH-Superfamily Protein | 1.09 | 16.32 | 8.01 | 10.27 | 4.71 | 3.91 | 2.88 | 3.24 | 2.11 |
| m.26606 | No | COTH-Superfamily Protein | 9.29 | 2.01 | 2.25 | 6.43 | 2.66 | -2.21 | -2.05 | -0.53 | -1.80 |
| m.26997 | No | COTH-Superfamily Protein | 9.77 | 16.66 | 3.03 | 3.68 | 5.01 | 0.77 | -1.69 | -1.41 | -0.96 |
| m.27030 | No | Predicted ser/Thr phosphohydrolase | 2.56 | 4.75 | 2.68 | 2.69 | 1.85 | 0.89 | 0.06 | 0.07 | -0.47 |
| m.27981 | No | CE:Acetylxylan Esterase | 2.70 | 4.99 | 0.84 | 0.19 | 0.56 | 0.88 | -1.68 | -3.82 | -2.28 |
| m.28055 | No | Conserved hypothetical protein | 0.93 | 2.71 | 1.67 | 0.99 | 0.34 | 1.54 | 0.83 | 0.09 | -1.47 |
| m.28278 | No | COTH-Superfamily Protein | 0.00 | 0.40 | 1.39 | 0.24 | 0.48 | 18.61 | 20.41 | 17.90 | 18.87 |
| m.28331 | No | COTH-Superfamily Protein | 7.20 | 0.61 | 1.34 | 1.93 | 0.96 | -3.57 | -2.43 | -1.90 | -2.91 |
| m.28353 | No | COTH-Superfamily Protein | 0.19 | 4.84 | 0.12 | 0.00 | 0.17 | 4.68 | -0.62 | -17.53 | -0.17 |
| m.28419 | No | COTH-Superfamily Protein | 0.03 | 1.71 | 0.47 | 0.11 | 0.35 | 5.70 | 3.82 | 1.73 | 3.41 |
| m.28443 | No | COTH-Superfamily Protein | 0.54 | 0.78 | 0.68 | 0.05 | 1.08 | 0.53 | 0.32 | -3.44 | 0.99 |
| m.28470 | No | COTH-Superfamily Protein | 0.71 | 0.11 | 0.29 | 0.37 | 1.23 | -2.64 | -1.27 | -0.95 | 0.80 |
| m.28493 | No | COTH-Superfamily Protein | 3.16 | 0.89 | 0.14 | 0.00 | 0.13 | -1.83 | -4.48 | -21.59 | -4.59 |
| m.2922 | No | COTH-Superfamily Protein | 148.34 | 101.12 | 134.09 | 201.98 | 122.60 | -0.55 | -0.15 | 0.45 | -0.27 |
| m.3108 | No | COTH-Superfamily Protein | 33.08 | 77.38 | 128.24 | 121.90 | 146.37 | 1.23 | 1.95 | 1.88 | 2.15 |
| m.3144 | No | COTH-Superfamily Protein | 43.20 | 59.95 | 75.87 | 114.05 | 45.43 | 0.47 | 0.81 | 1.40 | 0.07 |
| m.3145 | Yes | GH43: alpha-N-arabinofuranosidase | 201.96 | 268.89 | 227.48 | 259.77 | 255.01 | 0.41 | 0.17 | 0.36 | 0.34 |
| m.3264 | Yes | GH3:beta-glucosidase | 25.90 | 27.56 | 44.66 | 22.65 | 11.07 | 0.09 | 0.79 | -0.19 | -1.23 |
| m.3269 | Yes | GH3:beta-glucosidase | 289.01 | 214.98 | 307.33 | 629.45 | 435.34 | -0.43 | 0.09 | 1.12 | 0.59 |
| m.3416 | No | COTH-Superfamily Protein | 47.52 | 20.03 | 9.63 | 16.74 | 10.65 | -1.25 | -2.30 | -1.51 | -2.16 |
| m.3534 | No | COTH-Superfamily Protein | 21.70 | 12.61 | 8.24 | 7.31 | 5.34 | -0.78 | -1.40 | -1.57 | -2.02 |
| m.3920 | No | COTH-Superfamily Protein | 9.17 | 23.17 | 11.04 | 13.69 | 21.75 | 1.34 | 0.27 | 0.58 | 1.25 |
| m.4221 | Yes | GH10: endo-1 4-beta-xylanase | 24.46 | 15.88 | 31.72 | 31.77 | 24.71 | -0.62 | 0.38 | 0.38 | 0.01 |
| m.4245 | No | COTH-Superfamily Protein | 6.64 | 0.05 | 0.51 | 0.07 | 0.15 | -7.02 | -3.71 | -6.56 | -5.51 |
| m.4309 | No | COTH-Superfamily Protein | 11.87 | 2.92 | 34.64 | 209.27 | 70.44 | -2.03 | 1.54 | 4.14 | 2.57 |
| m.4359 | No | COTH-Superfamily Protein | 126.76 | 155.39 | 86.29 | 116.67 | 56.99 | 0.29 | -0.55 | -0.12 | -1.15 |
| m.4360 | No | Hypothetical Unknown | 32.06 | 52.53 | 38.82 | 74.69 | 35.30 | 0.71 | 0.28 | 1.22 | 0.14 |
| m.4438 | Yes | GH95: alpha l fucosidase | 39.67 | 37.95 | 20.51 | 17.85 | 33.50 | -0.06 | -0.95 | -1.15 | -0.24 |
| m.4480 | Yes | GH8: Endoglucanase | 113.21 | 30.34 | 30.81 | 14.02 | 99.22 | -1.90 | -1.88 | -3.01 | -0.19 |
| m.4522 | No | COTH-Superfamily Protein | 132.74 | 187.24 | 31.49 | 28.12 | 46.83 | 0.50 | -2.08 | -2.24 | -1.50 |
| m.4600 | No | COTH-Superfamily Protein | 2.45 | 9.24 | 7.11 | 3.73 | 13.75 | 1.91 | 1.54 | 0.61 | 2.49 |
| m.4606 | No | Alkyl transferase | 27.87 | 34.18 | 34.99 | 21.94 | 108.65 | 0.29 | 0.33 | -0.35 | 1.96 |
| m.4753 | No | COTH-Superfamily Protein | 2.21 | 3.58 | 0.14 | 0.35 | 2.74 | 0.70 | -3.97 | -2.66 | 0.31 |
| m.4778 | No | Conserved hypothetical protein | 2.71 | 14.66 | 14.86 | 4.41 | 3.53 | 2.44 | 2.46 | 0.70 | 0.38 |
| m.4856 | Yes | GH:xyloglucanase | 5.41 | 28.32 | 12.35 | 11.75 | 6.83 | 2.39 | 1.19 | 1.12 | 0.34 |
| m.4984 | No | Conserved hypothetical protein | 1.59 | 4.64 | 0.36 | 0.09 | 0.25 | 1.55 | -2.16 | -4.21 | -2.69 |
| m.5143 | No | COTH-Superfamily Protein | 0.98 | 10.90 | 8.66 | 6.31 | 4.67 | 3.48 | 3.15 | 2.69 | 2.26 |
| m.516 | Yes | GH43: alpha-N-arabinofuranosidase | 35.37 | 12.96 | 3.13 | 2.49 | 14.20 | -1.45 | -3.50 | -3.83 | -1.32 |
| m.5185 | No | COTH-Superfamily Protein | 91.41 | 71.41 | 115.84 | 150.11 | 57.39 | -0.36 | 0.34 | 0.72 | -0.67 |
| m.5222 | Yes | GH48: CellobiohydrolaseGH48 | 5.99 | 17.53 | 21.40 | 10.19 | 20.09 | 1.55 | 1.84 | 0.77 | 1.75 |
| m.5263 | Yes | GH10: endo-1 4-beta-xylanase | 148.50 | 49.60 | 26.59 | 54.57 | 61.74 | -1.58 | -2.48 | -1.44 | -1.27 |
| m.5378 | No | Conserved hypothetical protein | 28.92 | 19.35 | 26.41 | 49.75 | 30.24 | -0.58 | -0.13 | 0.78 | 0.06 |
| m.5379 | Yes | GH10: endo-1 4-beta-xylanase | 10.29 | 4.87 | 14.85 | 28.34 | 18.41 | -1.08 | 0.53 | 1.46 | 0.84 |
| m.5380 | No | Conserved hypothetical protein | 85.73 | 71.33 | 82.38 | 99.22 | 56.53 | -0.27 | -0.06 | 0.21 | -0.60 |
| m.5429 | No | COTH-Superfamily Protein | 3.13 | 2.18 | 3.60 | 0.81 | 6.33 | -0.52 | 0.20 | -1.94 | 1.02 |
| m.5519 | No | COTH-Superfamily Protein | 3.44 | 17.35 | 6.86 | 6.45 | 4.64 | 2.33 | 0.99 | 0.91 | 0.43 |
| m.5563 | No | COTH-Superfamily Protein | 0.81 | 0.76 | 0.09 | 0.00 | 1.57 | -0.11 | -3.24 | -19.64 | 0.95 |
| m.5588 | No | COTH-Superfamily Protein | 1.56 | 1.41 | 1.37 | 2.31 | 2.39 | -0.15 | -0.19 | 0.56 | 0.61 |
| m.5608 | No | COTH-Superfamily Protein | 1.14 | 4.84 | 3.02 | 0.52 | 0.69 | 2.09 | 1.41 | -1.11 | -0.72 |
| m.5698 | No | COTH-Superfamily Protein | 0.50 | 1.39 | 2.10 | 2.57 | 2.86 | 1.47 | 2.07 | 2.36 | 2.52 |
| m.5850 | No | COTH-Superfamily Protein | 0.00 | 2.51 | 5.90 | 0.00 | 0.00 | 21.26 | 22.49 | 0.00 | 0.00 |
| m.5886 | No | COTH-Superfamily Protein | 3.16 | 4.89 | 1.50 | 4.19 | 6.71 | 0.63 | -1.08 | 0.41 | 1.08 |
| m.5963 | No | COTH-Superfamily Protein | 15.95 | 5.86 | 4.94 | 9.58 | 5.59 | -1.44 | -1.69 | -0.74 | -1.51 |
| m.6037 | No | COTH-Superfamily Protein | 6.49 | 60.20 | 35.14 | 14.63 | 10.54 | 3.21 | 2.44 | 1.17 | 0.70 |
| m.6127 | No | Hypothetical Unknown | 0.82 | 7.90 | 5.65 | 3.47 | 2.10 | 3.27 | 2.79 | 2.08 | 1.36 |
| m.6214 | No | COTH-Superfamily Protein | 0.32 | 1.64 | 1.21 | 0.67 | 2.33 | 2.37 | 1.92 | 1.07 | 2.87 |
| m.6239 | No | Conserved hypothetical protein | 0.66 | 1.64 | 2.06 | 0.32 | 0.29 | 1.31 | 1.63 | -1.05 | -1.21 |
| m.6300 | No | CE:Feruoyl Esterase | 0.69 | 0.43 | 0.68 | 2.55 | 1.45 | -0.68 | -0.03 | 1.88 | 1.07 |
| m.6433 | No | Hypothetical Unknown | 19.67 | 0.12 | 5.54 | 4.08 | 0.76 | -7.36 | -1.83 | -2.27 | -4.70 |
| m.8475 | No | COTH-Superfamily Protein | 1.47 | 1.43 | 0.58 | 0.41 | 1.23 | -0.04 | -1.34 | -1.84 | -0.25 |
| m.8608 | No | COTH-Superfamily Protein | 0.23 | 0.00 | 0.56 | 0.71 | 0.77 | -17.82 | 1.27 | 1.62 | 1.73 |
| m.8620 | No | COTH-Superfamily Protein | 1.15 | 0.44 | 2.58 | 1.12 | 2.99 | -1.37 | 1.16 | -0.04 | 1.38 |
| m.8660 | No | COTH-Superfamily Protein | 0.51 | 0.00 | 0.38 | 0.72 | 1.31 | -18.97 | -0.43 | 0.48 | 1.35 |
| m.8676 | No | COTH-Superfamily Protein | 0.37 | 1.83 | 0.77 | 0.88 | 1.05 | 2.29 | 1.05 | 1.23 | 1.50 |
| m.8737 | No | Conserved hypothetical protein | 1.70 | 1.81 | 1.88 | 4.05 | 2.42 | 0.09 | 0.14 | 1.25 | 0.51 |
| m.8798 | No | Hypothetical Unknown | 20.37 | 34.41 | 9.27 | 15.03 | 6.79 | 0.76 | -1.14 | -0.44 | -1.59 |
| m.9294 | No | COTH-Superfamily Protein | 4.43 | 2.11 | 5.99 | 6.23 | 5.42 | -1.07 | 0.44 | 0.49 | 0.29 |
| m.9295 | No | COTH-Superfamily Protein | 5.71 | 4.93 | 16.05 | 19.25 | 9.54 | -0.21 | 1.49 | 1.75 | 0.74 |
| m.9329 | Yes | GH2: beta-galactosidase | 6.47 | 8.25 | 1.24 | 1.17 | 3.68 | 0.35 | -2.39 | -2.47 | -0.81 |
| m.9364 | No | COTH-Superfamily Protein | 26.27 | 30.82 | 19.21 | 29.99 | 173.51 | 0.23 | -0.45 | 0.19 | 2.72 |
| m.9610 | Yes | GH10: endo-1 4-beta-xylanase | 340.05 | 422.93 | 542.80 | 851.81 | 645.86 | 0.31 | 0.67 | 1.32 | 0.93 |
| m.9627 | No | COTH-Superfamily Protein | 325.37 | 220.77 | 239.39 | 370.17 | 241.53 | -0.56 | -0.44 | 0.19 | -0.43 |
| m.9664 | Yes | GH: xylan α-1,2-glucuronidase | 91.43 | 21.09 | 6.09 | 2.55 | 4.41 | -2.12 | -3.91 | -5.16 | -4.37 |
| m.9699 | No | CE:Feruoyl Esterase | 0.20 | 2.83 | 2.39 | 0.65 | 0.42 | 3.84 | 3.60 | 1.73 | 1.07 |
| m.9700 | No | CE:Feruoyl Esterase | 3.95 | 21.44 | 18.22 | 3.51 | 3.07 | 2.44 | 2.20 | -0.17 | -0.36 |
| m.9712 | Yes | GH45: EndoGlucanse | 139.71 | 192.68 | 121.30 | 92.21 | 94.65 | 0.46 | -0.20 | -0.60 | -0.56 |
| m.9713 | No | CE:Feruoyl Esterase | 6.03 | 33.09 | 72.78 | 0.00 | 503.73 | 2.46 | 3.59 | -22.52 | 6.38 |
| m.9725 | No | COTH-Superfamily Protein | 4.56 | 9.56 | 8.88 | 5.22 | 5.94 | 1.07 | 0.96 | 0.20 | 0.38 |
| m.9762 | Yes | GH45: Endoglucanase | 234.29 | 95.73 | 131.07 | 132.92 | 48.52 | -1.29 | -0.84 | -0.82 | -2.27 |
| m.9764 | No | COTH-Superfamily Protein | 23.93 | 2.59 | 5.31 | 20.69 | 6.98 | -3.21 | -2.17 | -0.21 | -1.78 |
| m.9765 | No | COTH-Superfamily Protein | 25.58 | 84.13 | 95.86 | 91.22 | 44.62 | 1.72 | 1.91 | 1.83 | 0.80 |
| m.9889 | No | Predicted ser/Thr phosphohydrolase | 1.15 | 0.35 | 1.06 | 4.27 | 1.04 | -1.72 | -0.12 | 1.89 | -0.15 |

^a^ Fold change is shown as Log_2_ expression levels compared to glucose. Color code: Green= significantly over-expressed (a differential expression p-value <0. 1 as calculated by the nbinomTest function in the R package DESeq), red= significantly under-expressed (a differential expression p-value <0.1 as calculated by the nbinomTest function in the R package DESeq).

**Table S5. Cellulose and hemicellulose composition of the plant material used in this study.**

| **Plant substrate** | **% Cellulose** | **% Hemicellulose** |
| --- | --- | --- |
| **Alfalafa** | 42.42 | 30.3 |
| **Energy Cane** | 40.63 | 34.38 |
| **Corn Stover** | 45.46 | 36.36 |
| **Sorghum** | 43.9 | 31.71 |

**Table S6. Trnascriptional levels of all major families of FDD-containing transcripts when grown on glucose and lignocellulosic biomass substrates.**

| Family | Number of transcripts | Normalized FPKM^1^ | | | | | Log2 fold expression^2^ | | | |
| --- | --- | --- | --- | --- | --- | --- | --- | --- | --- | --- |
|  |  | Glucose | Alfalfa | Energy Cane | Corn Stover | Sorghum | Alfalfa | Energy Cane | Corn Stover | Sorghum |
| Hypothetical and conserved hypothetical proteins | 42 | 1320.73 | 1521.46 | 1310.77 | 1227.58 | 1129.88 | 0.20 | -0.01 | -0.11 | -0.23 |
| CotH family proteins | 129 | 5545.80 | 5126.31 | 5855.17 | 5694.44 | 6223.82 | -0.11 | 0.08 | 0.04 | 0.17 |
| GHs and accessory enzymes | 104 | 9108.52 | 5574.69 | 6464.61 | 8957.58 | 6515.76 | -0.71 | -0.49 | -0.02 | -0.48 |
| Proteases, serpins, and protein phosphatases | 21 | 878.70 | 1206.46 | 1080.48 | 1079.50 | 1280.49 | 0.46 | 0.30 | 0.30 | 0.54 |

^1^: Corrected FPKM values normalized by the library size, as calculated using the estimateDispersions function in the R package DESeq [44].

^2^: Fold change is shown as Log_2_ expression levels compared to glucose. Color code: Green= significantly over-expressed (a differential expression p-value <0.01 as calculated by the nbinomTest function in the R package DESeq), red= significantly under-expressed (a differential expression p-value <0.01 as calculated by the nbinomTest function in the R package DESeq [44]
